# Supplementary material for: A Molecular Mechanism of Intrahepatic Cholestasis in Osteo-Oto-Hepato-Enteric Syndrome
Source: Cell Mol Gastroenterol Hepatol. 2026 May 20;20(9):101805. doi: 10.1016/j.jcmgh.2026.101805 (PMC13324508; doi:10.1016/j.jcmgh.2026.101805)
Supplement: Supplementary Table 1 [file mmc1.pdf]

Supplementary table 1. Overview of all O2HE-associated UNC45A variants, predicted effects on amino acid changes, predicted consequences on protein structure and function and experimentally demonstrated consequences. Figure shows only missense and deletion variants.

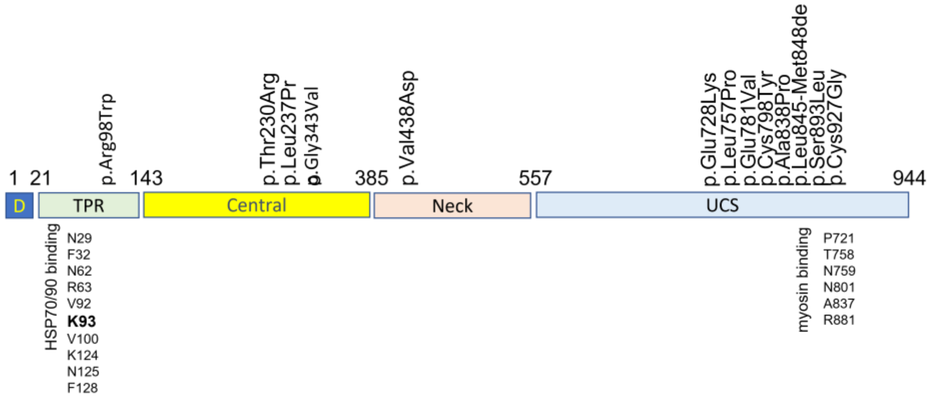

Figure on the left shows missense variants only.

<u>HSP70/90-binding residues are inferred from:</u>

Gazda L, Pokrzywa W, Hellerschmied D, et al. The myosin chaperone UNC-45 is organized in tandem modules to support myofilament formation in C. elegans. Cell. 2013 Jan 17;152(1-2):183-95. doi: 10.1016/j.cell.2012.12.025.

<u>Myosin-binding residues are inferred from:</u>

Vogel A, Arnese R, Gudino Carrillo RM, et al., UNC-45 assisted myosin folding depends on a conserved FX3HY motif implicated in Freeman Sheldon Syndrome. Nat Commun. 2024 Jul 25;15(1):6272. doi: 10.1038/s41467-024-50442-6.

Reference PUBMED ID	Patient ID	Gene variant	Protein prediction	Domain	Predicted consequences on protein structure and function#	Experimentally demonstrated consequences
29429573	P7[1]	c.247C > T	p.Arg83Trp[1]	TPR	See footnote [1]	See footnote [1]
36699472/<br>41861679/<br>29429573[1]	P3/<br>P1/<br>P7[1]	c.292C > T	p.Arg98Trp	TPR	The bulky Trp side chain is not compatible with the neighboring helices positioned within the TPR domain, it might affect the HSP90/70 binding site within the TPR domain	<u>This study:</u><br>- Reduced expression in patient liver tissue (with additional Gln198∗ variant)<br>- Reduced interaction with HSP70<br>- Defective myosin Vb function.<br>- Defective Rab11a-mediated BSEP trafficking<br>- Aberrant BSEP IHC in patient liver tissue (with additional Gln198∗ variant)

41861679	P1	c.592C>T	Gln198*	Central	Protein truncated at the central domain, protein expression is inhibited	- No protein expression.
35575086	P4	c.689C > G	p.Thr230Arg	Central	Mapped in the helix 1 of the 3rd ARM repeat (3H1) of the central domain, introducing the long side chain of an Arg at the position of the Thr might result in steric hindrance with surrounding residues destabilizing the protein. May alter the positioning of the TRP domain and thus protein conformation.	PMID 35575086:<br>- No effect on protein expression when expressed in HEK cells
36587802/ 35575086/ 35575086/ 35575086	P2/ P1/ P5/ P6	c.710 T > C	p.Leu237Pro	Central	Mapped in the helix 1 of the 3rd ARM repeat (3H1) of the central domain, compatible with the structure; however, may result in loss of a key backbone H-bond and in helix destabilization and a local structural rearrangement. May alter the positioning of the TRP domain and thus protein conformation.	PMID 36587802:<br>- protein expression of the UNC45A-Leu237Pro group decreased by 27.4% when expressed in HEK cells<br><br>PMID 35575086:<br>- No effect on protein expression when expressed in HEK cells<br><br>PMID 40125554:<br>-in T lymphoblastic cells and in fibroblasts isolated from skin biopsies from two patients, protein levels are comparable to those of control cells.<br>- Disrupts chaperone-myosin interaction.<br>- Retained chaperone activity.<br>- Prevented myosin aggregation.<br>- Supported NMII filament formation in patient fibroblasts and U2OS cells.<br>- Formed atypically stable oligomers.

						- Prevented chaperone-myosin complex dissociation.<br>- Inhibited NMII functions.<br>- Impaired intracellular trafficking.<br>- considerably lowered the thermal resistance of UNC45A
35575086	P2	c.721C > T	p.Arg241*	Central	Protein truncated within the central domain is predicted to inhibit expression.	PMID 35575086:<br>- No protein expression when expressed in cultured cells
29429573	P4	c.784C > T	p.Arg262*[2]<br>p.Arg277*	Central	The truncation generates a protein with a short, unstable central domain and lacking both the neck and UCS domains.	- No protein expression
29429573	P7	c.983G > T	p.Gly328Val[2]<br>p.Gly343Val	Central	The residue substitution is compatible with the protein's structural integrity.	ND
29429573	P4	c.1268 T > A	p.Val423Asp[2]<br>p.Val438Asp	Neck	In the neck domain, substitution of the hydrophobic valine with the charged aspartate in the hydrophobic core is likely to destabilize the protein.	PMID 35421597:<br>- Unstable protein.<br>- Retained chaperone activity.<br>(Based on Val423Asp)
35575086	P3	c.1452delins GCA	p.Asp484Glu fs* 17	Neck	Truncation in the middle of the central domain is predicted to abolish chaperone activity, inhibit protein expression, and render the protein unstable (35575086).	PMID 35575086:<br>- No protein expression when expressed in cultured cells
35575086	P2	c.2182G > A	p.Glu728Lys	UCS	In highly conserved motifs of 2 different helices (13H2 and 15H3) within the UCS domain. This surface-residue mutation in the UCS domain is compatible with the overall protein structure. Possibly induces electrostatic repulsion and	PMID 35575086:<br>- No effect on protein expression when expressed in cells

					surface charge alterations that may affect interactions between the 2 UCS helices and induce a local structural rearrangement.	
39403551	P1	c.2225 T>C	p.Leu742Pro[2]<br>p.Leu757Pro	UCS	The rigid pyrrolidine ring of proline may cause substantial changes in peptide chain backbone angles destabilize helices. This residue is next to T758 and N759 which are directly involved in myosin motor domain interaction.	ND
39403551	P1	c.2297 A>T	p.Glu766Val[2]<br>p.Glu781Val	UCS	Because Glu is often found on the exterior of proteins interacting with water, and Val is usually hidden in the interior, this substitution can lead to severe protein instability or improper conformation.	ND
39403551	P1	c.2348 G>A	p.Cys783Tyr[2]<br>p.Cys798Tyr	UCS	Because Tyr cannot form disulfide bonds, replacing a critical Cys residue often destroys existing disulfide bridges, and may cause disruptions in the protein's native tertiary structure.	ND
41081434/<br>36587802	P1/<br>P2	c.2455C > T	p.Arg819*	UCS	The truncation results in an incomplete UCS domain, generating an unstable protein and inhibiting protein expression.	PMID 36587802:<br>- No protein expression when expressed in HEK cells
35575086	P3	c.2512G > C	p.Ala838Pro	UCS	In highly conserved motifs of 2 different helices (13H2 and 15H3) within the UCS domain. The proline residue may introduce a kink in the UCS domain helix, thereby destabilizing it.	PMID 35575086:<br>- No effect on protein expression when expressed in cells

36699472	P3	c.2534-2545del	p.Leu845-Met848del	UCS	The deletion of four residues shortens the UCS 15H-helix and also truncates the linker to the following helix, which may compromise the domain’s structural integrity and alter the conformation of the myosin-binding groove.	ND
29429573/<br>29429573	P5/<br>P6	c.2581C > T	p.Gln861*[2]<br>p.Gln876*	UCS	Truncation at position 876 results in a short, likely unstable UCS domain, which would severely destabilize the myosin-binding groove.	- No protein expression (in patient cells carrying in addition p.Ser893Leu and p.Cys927Gly)
29429573/<br>29429573	P5/<br>P6	c.2633C > T	p.Ser878Leu[2]<br>p.Ser893Leu	UCS	The structural consequences are mild; mutation of the polar surface serine to the hydrophobic leucine eliminates some stabilizing interactions and alters the surface properties, rendering it less polar.	- No protein expression (in patient cells carrying in addition p.Gln876* and p.Cys927Gly)
29429573/<br>29429573	P5/<br>P6	c.2734 T > G	p.Cys912Gly[2]<br>p.Cys927Gly	UCS	The substitution of cysteine by glycine introduces increased main-chain flexibility in the helix but remains compatible with the overall protein structure.	- No protein expression (in patient cells carrying in addition p.Gln876* and p.Ser893Leu)

ND: not determined

[#] The predicted consequences on protein structure/function were inferred from structural analysis of the protein residues localization and involvement in intramolecular interactions or in interactions with partner proteins, and have been reported before by: Sun M, Pylypenko O, Zhou Z, Xu M, Li Q, Houdusse A, van IJzendoorn SCD. Uncovering the Relationship Between Genes and Phenotypes Beyond the Gut in Microvillus Inclusion Disease. Cell Mol Gastroenterol Hepatol. 2024;17(6):983-1005. doi: 10.1016/j.jcmgh.2024.01.015, and by: Duclaux-Loras R, Lebreton C, Berthelet J, Charbit-Henrion F, Nicolle O, Revenu des Courtils C, Waich S, Valovka T, Khiat A, Rabant M, Racine C, Guerrera IC, Baptista J, Mahe MM, Hess MW, Durel B, Lefort N, Banal C, Parisot M, Talbotec C, Lacaille F, Ecochard-Dugelay E, Demir AM, Vogel GF, Faivre L, Rodrigues A, Fowler D, Janecke AR, Müller T, Huber LA, Rodrigues-Lima F, Ruemmele FM, Uhlig

HH, Del Bene F, Michaux G, Cerf-Bensussan N, Parlato M. UNC45A deficiency causes microvillus inclusion disease-like phenotype by impairing myosin VB-dependent apical trafficking. J Clin Invest. 2022 May 16;132(10):e154997. doi: 10.1172/JCI154997.

[1]. This variant was originally reported using the amino acid numbering of UNC45A isoform 2 (Q9H3U1-2; NP_001310548.1), which lacks a 15-amino acid segment at the N-terminus compared to the canonical sequence. To ensure consistency with the canonical reference (Q9H3U1-1; NP_061141.2) and the authors' cited reference transcript ENST00000394275.6 (944 aa), we have remapped the position to c.292C>T (p.Arg98Trp). All subsequent mentions of this variant follow the canonical numbering.

[2]. Variants marked with an uppercase [2] and displayed in grey were originally reported in PMID references 29429573 and 39403551 using the amino acid numbering of the shorter isoform Q9H3U1-2 (929 aa). For consistency within this table, positions have been corrected to match the authors' cited reference transcript ENST00000394275.6 (944 aa), resulting in a +15 amino acid offset from the original report.
