# Supplementary figures and images for: A Molecular Mechanism of Intrahepatic Cholestasis in Osteo-Oto-Hepato-Enteric Syndrome
Source: Cell Mol Gastroenterol Hepatol. 2026 May 20;20(9):101805. doi: 10.1016/j.jcmgh.2026.101805 (PMC13324508; doi:10.1016/j.jcmgh.2026.101805)

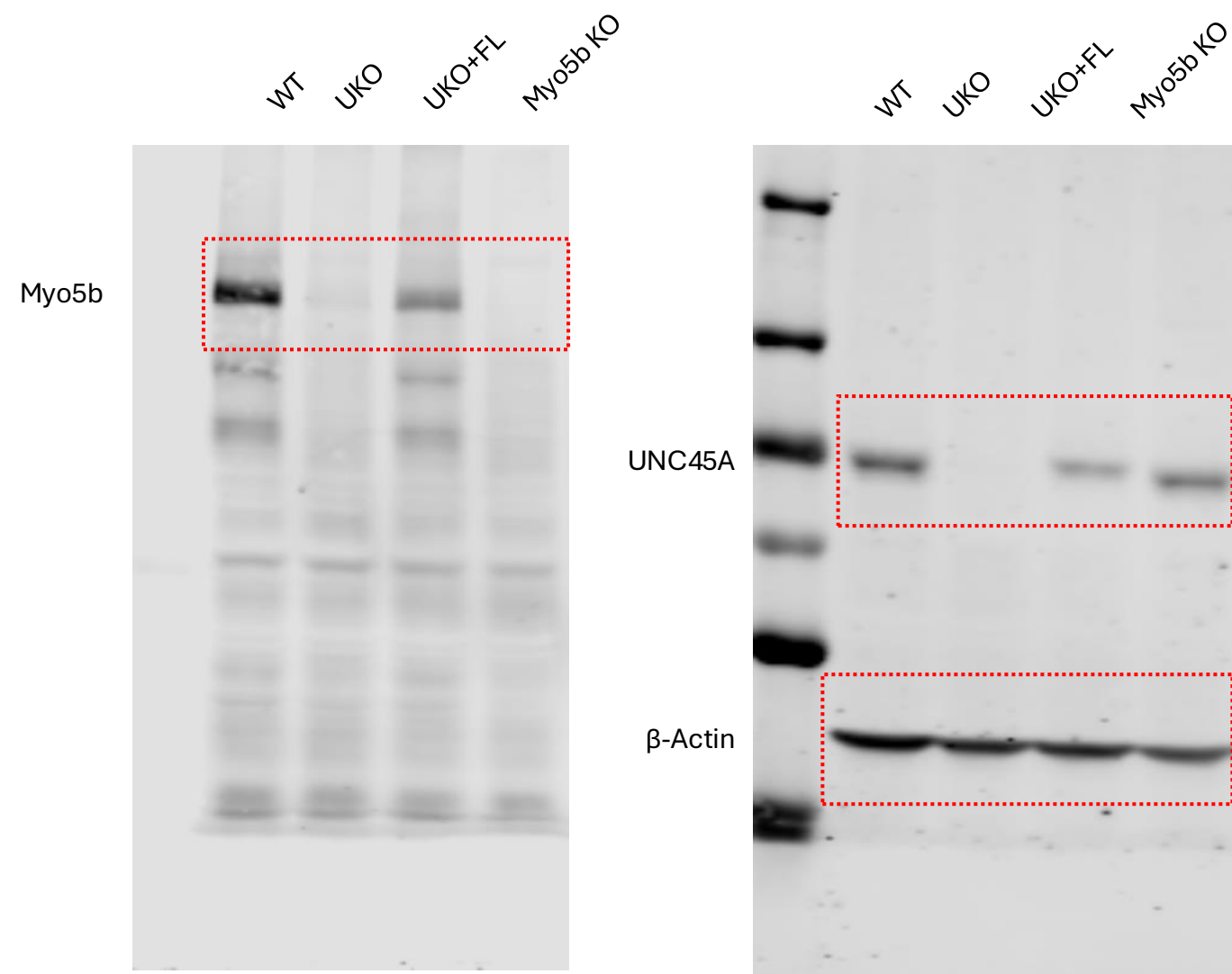

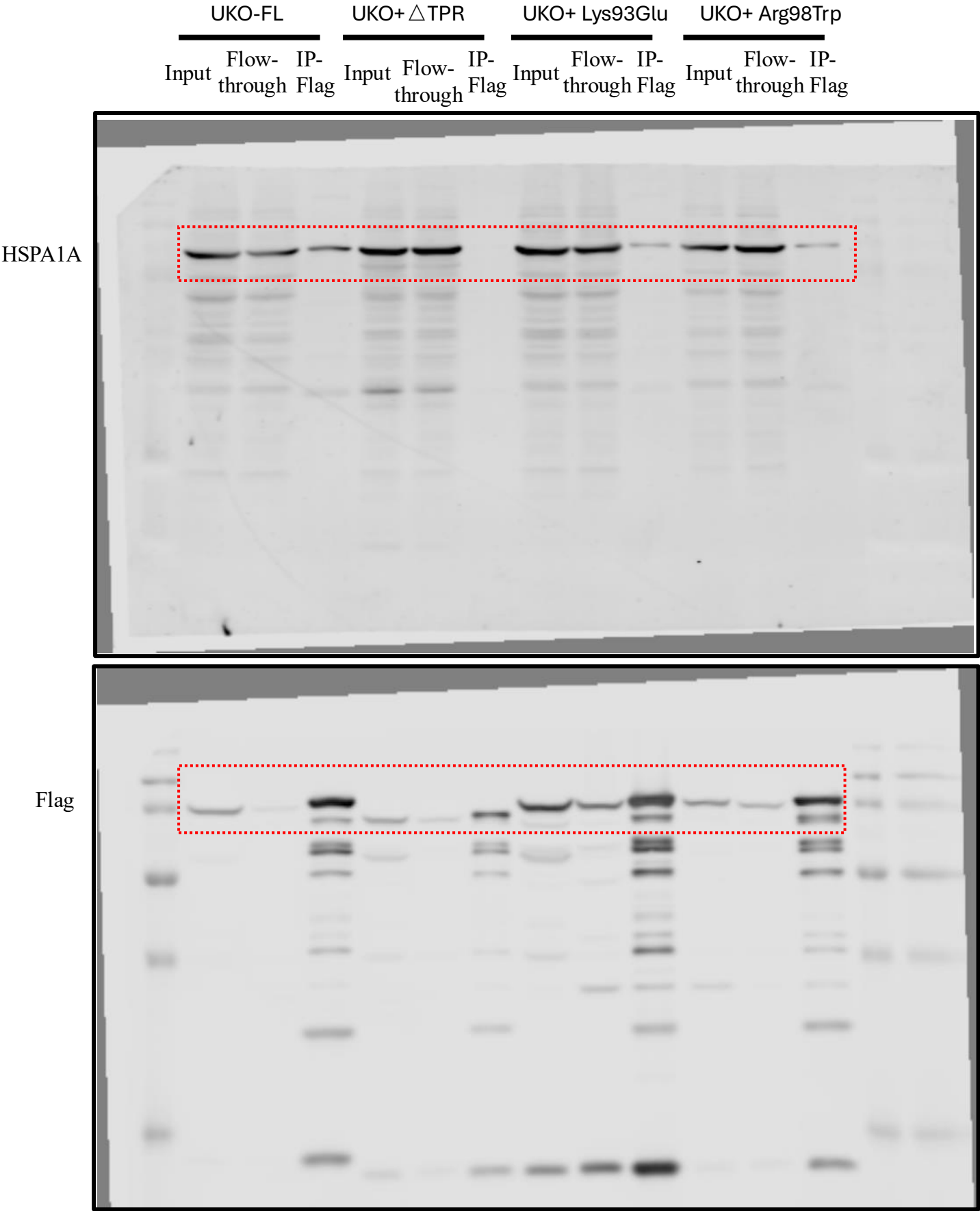

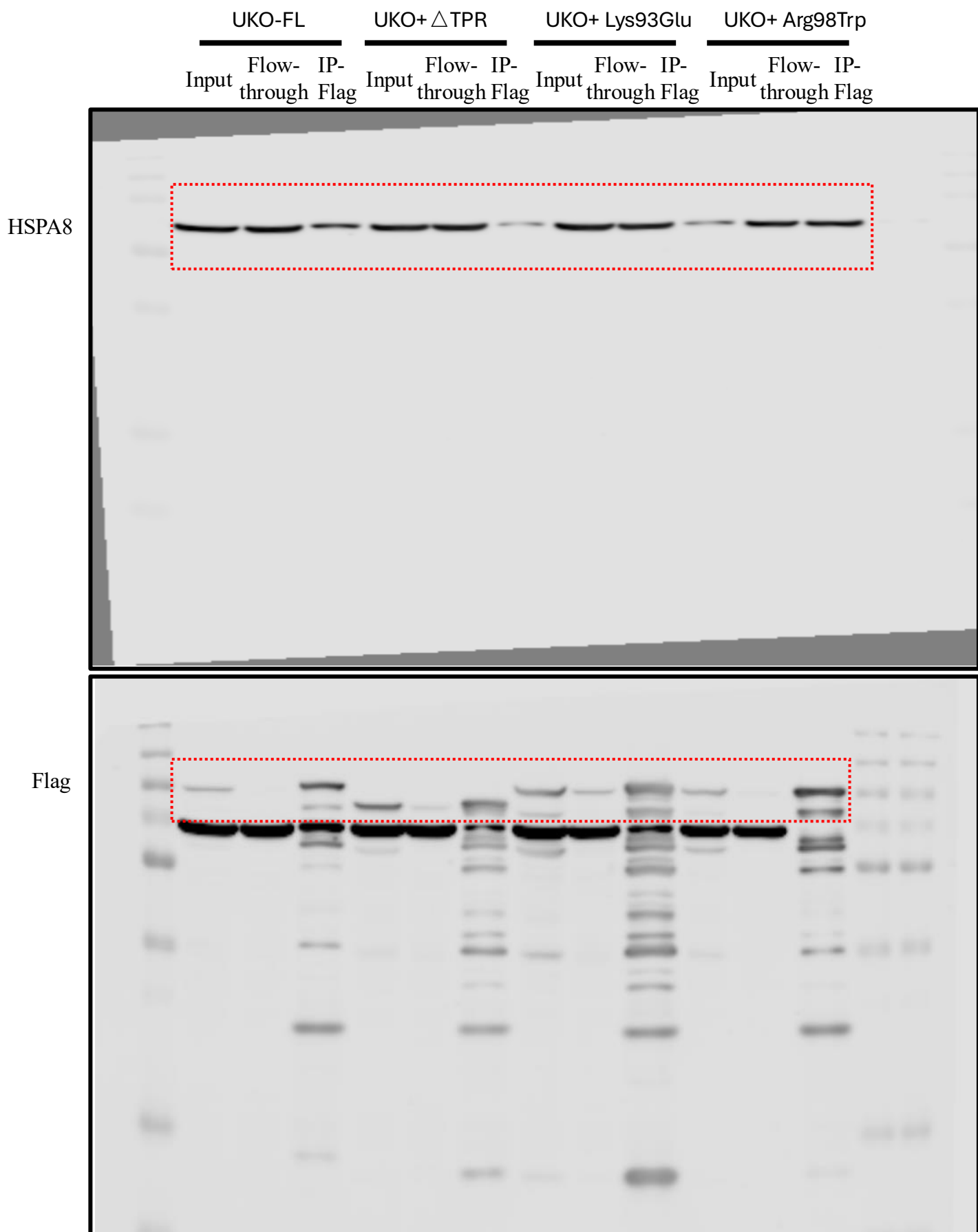

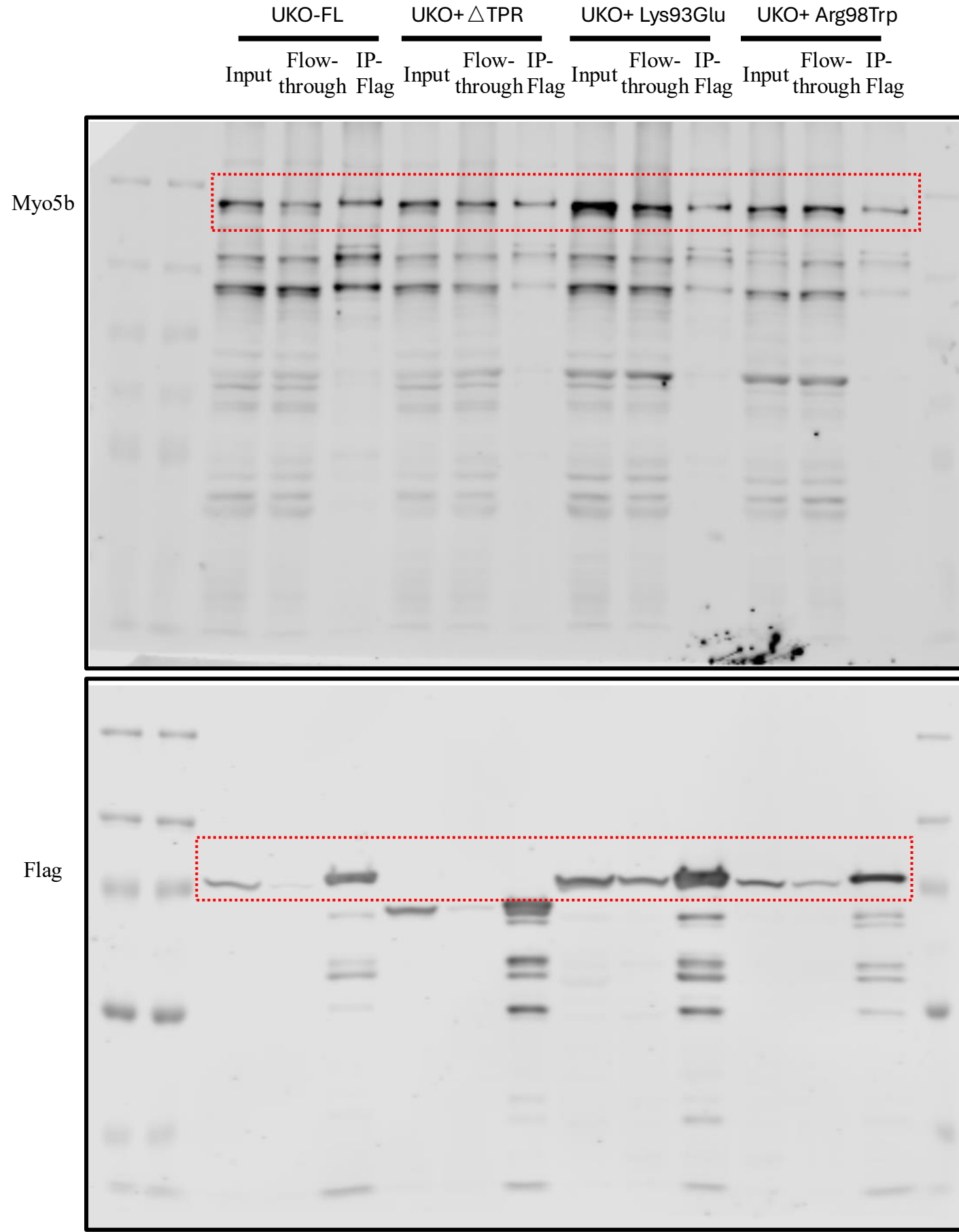

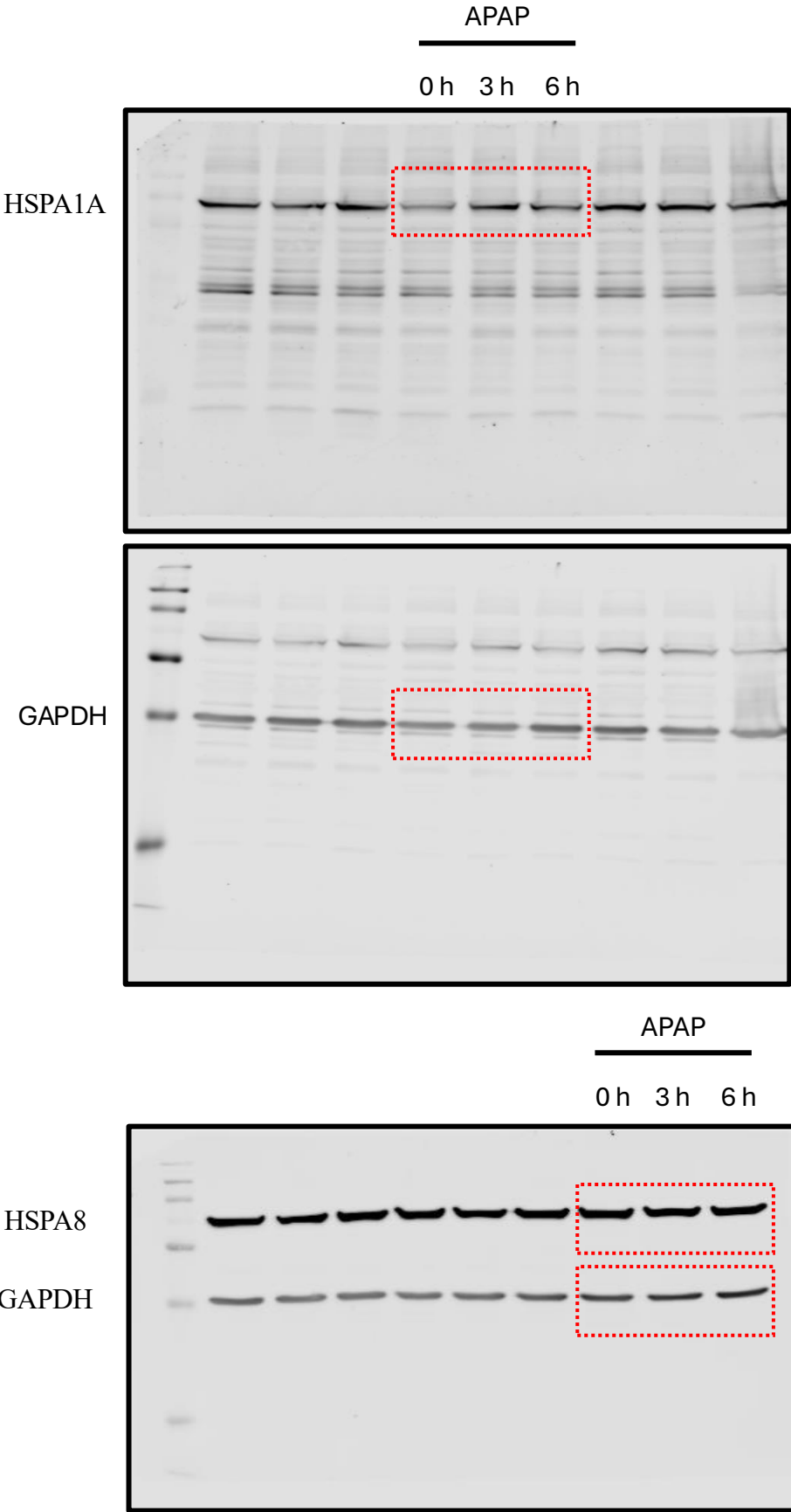

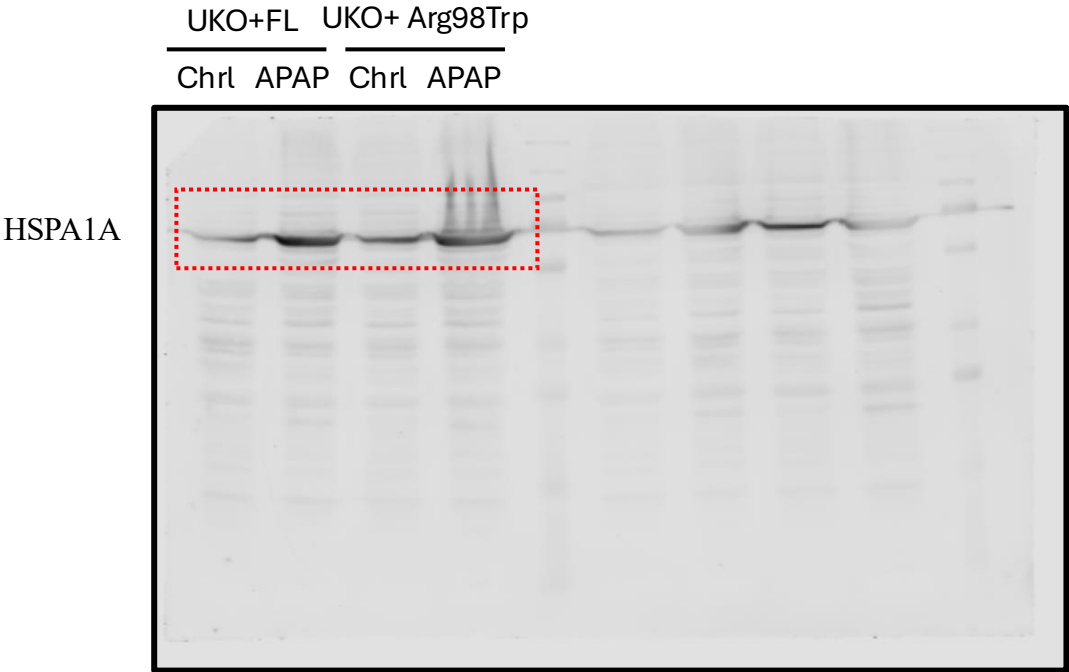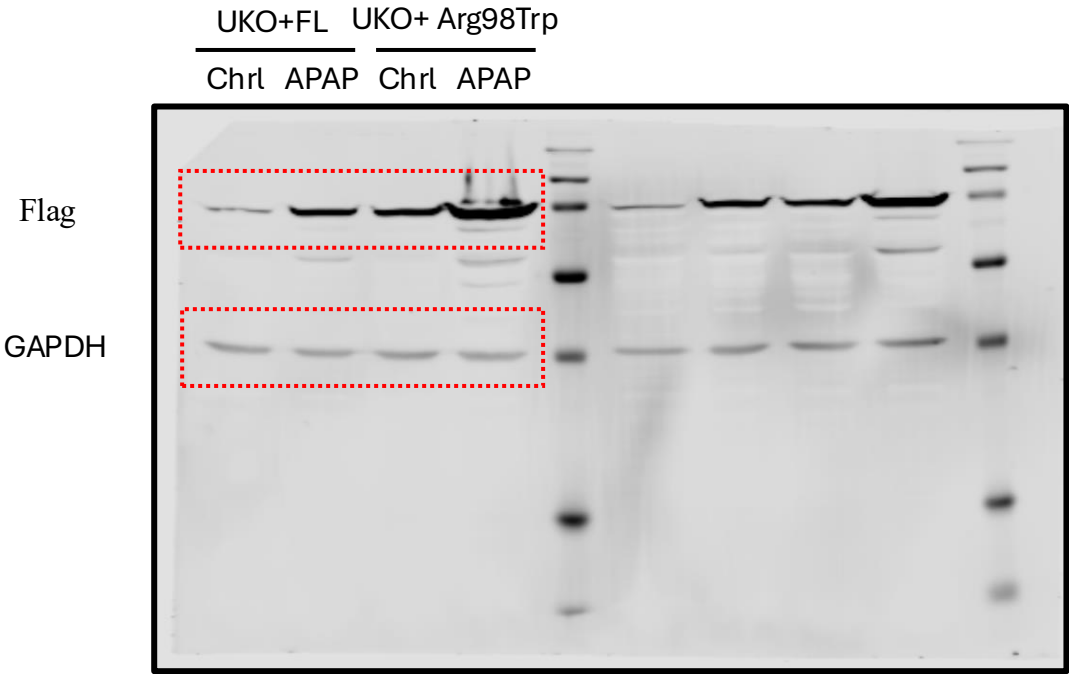

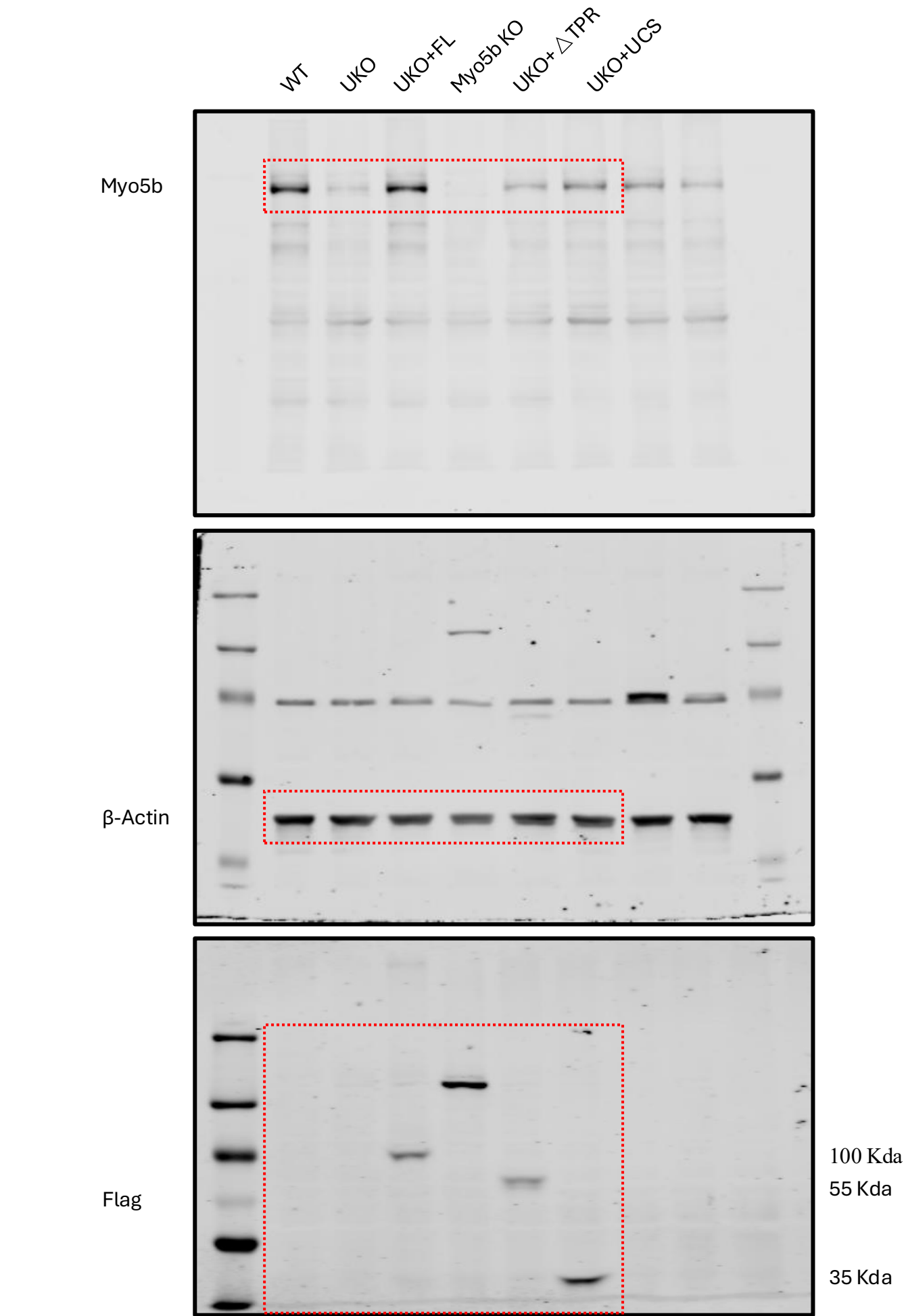

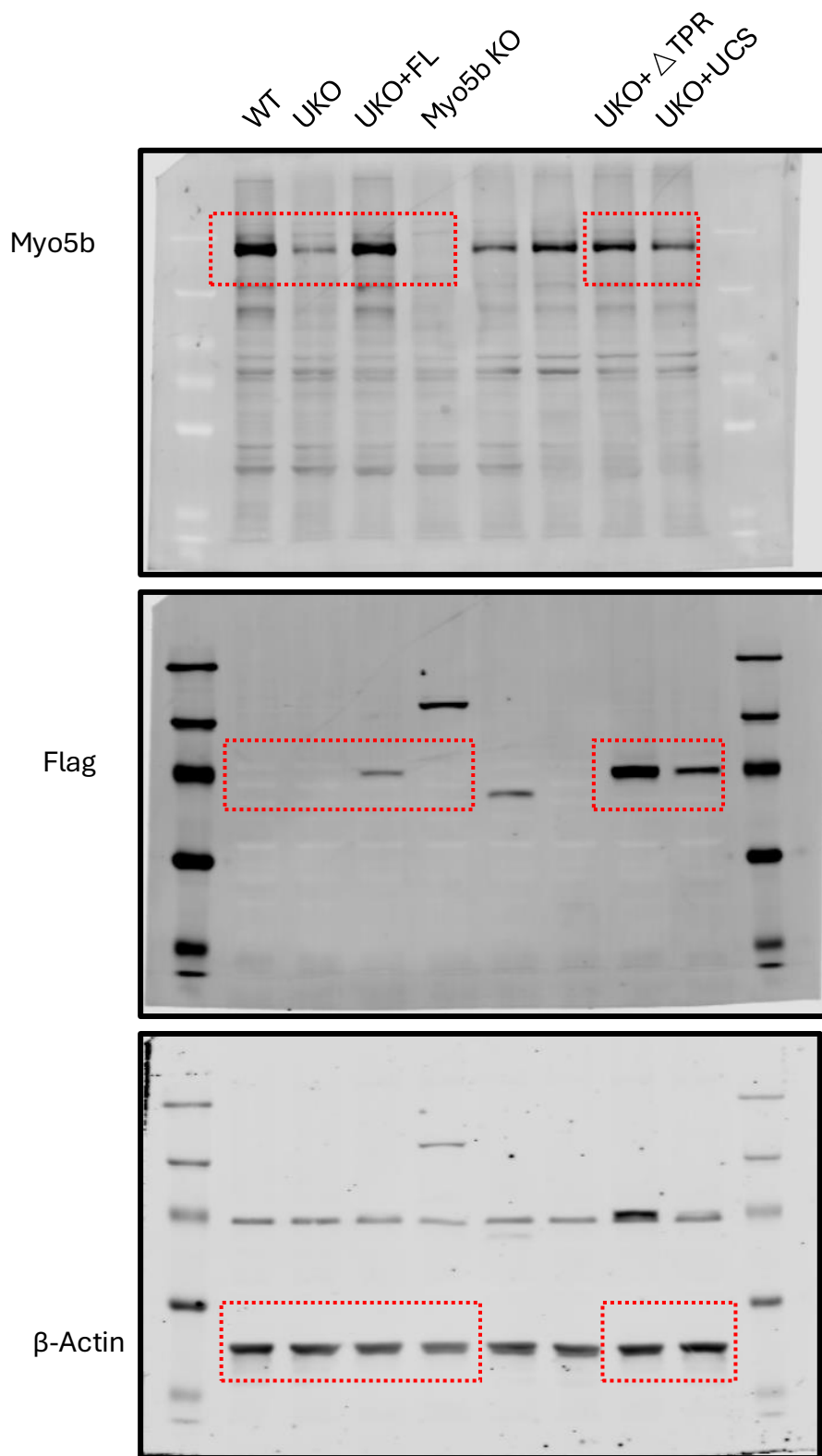

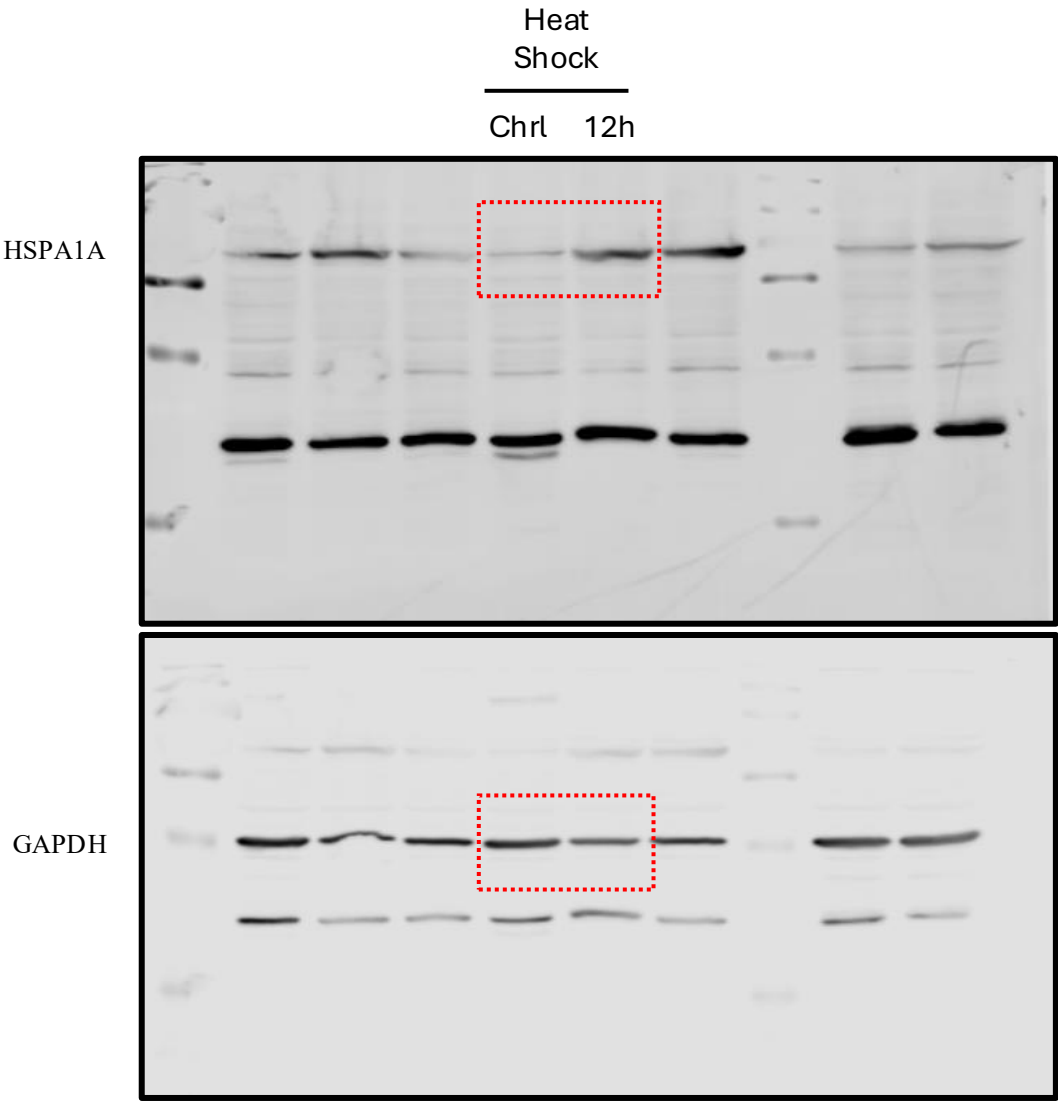

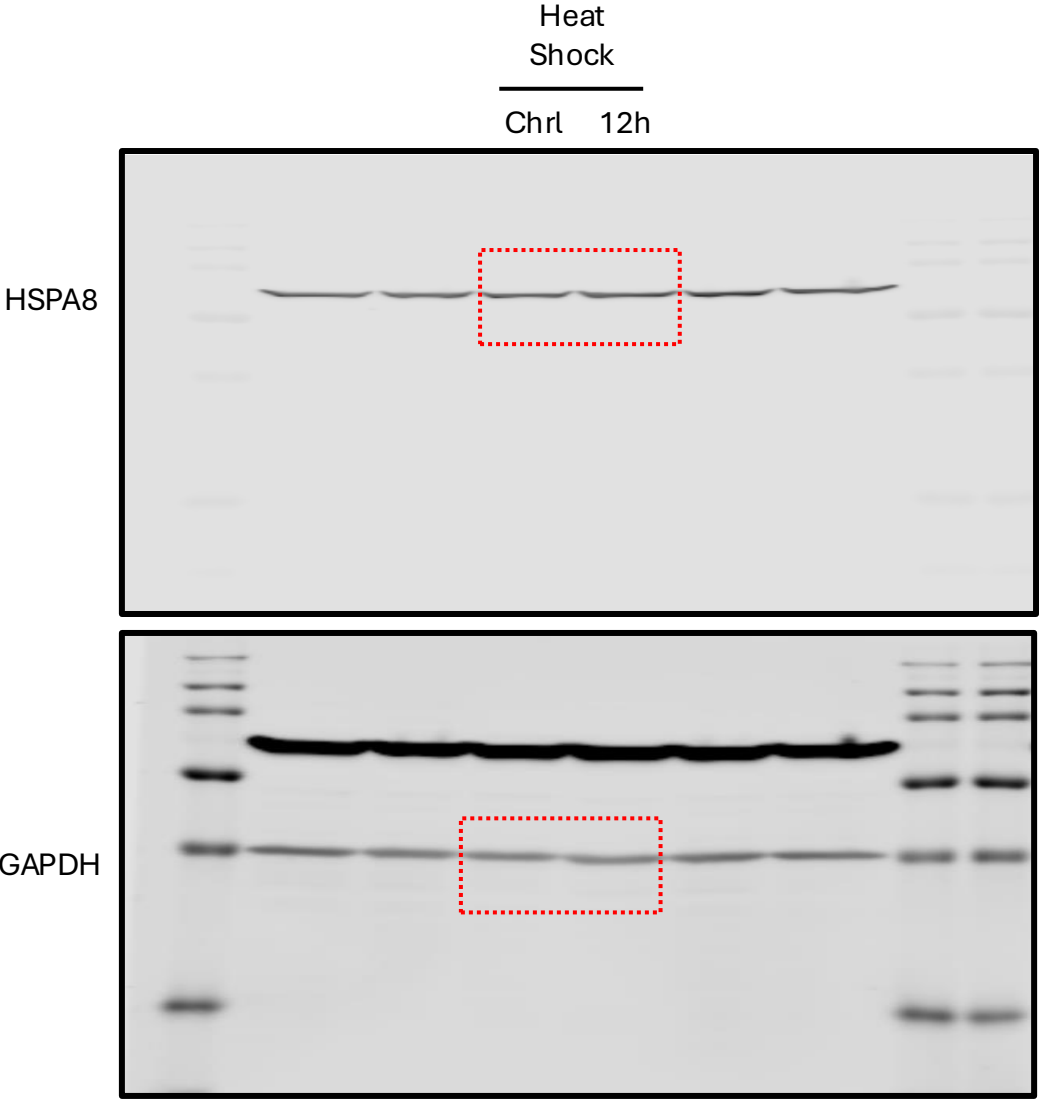

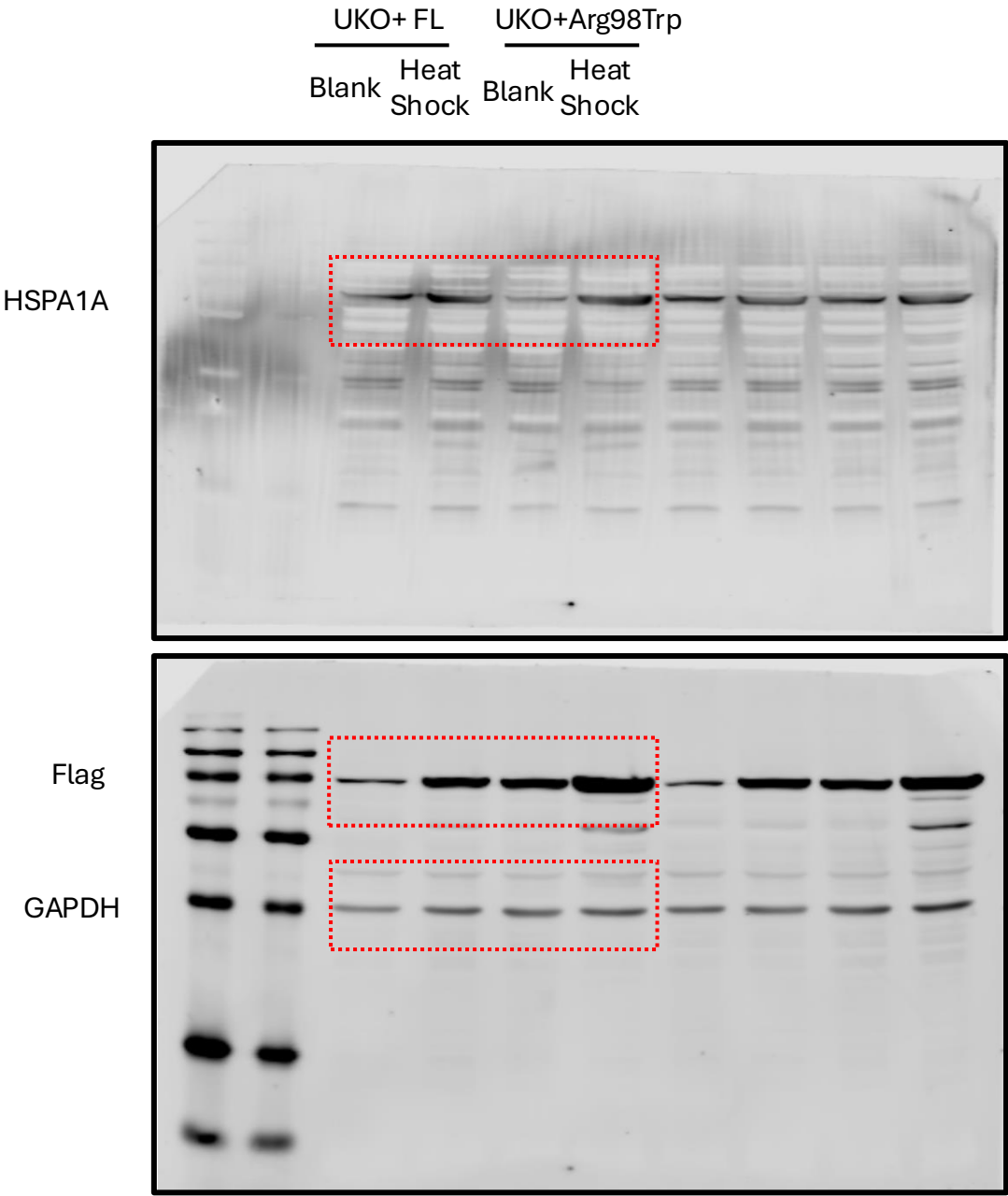

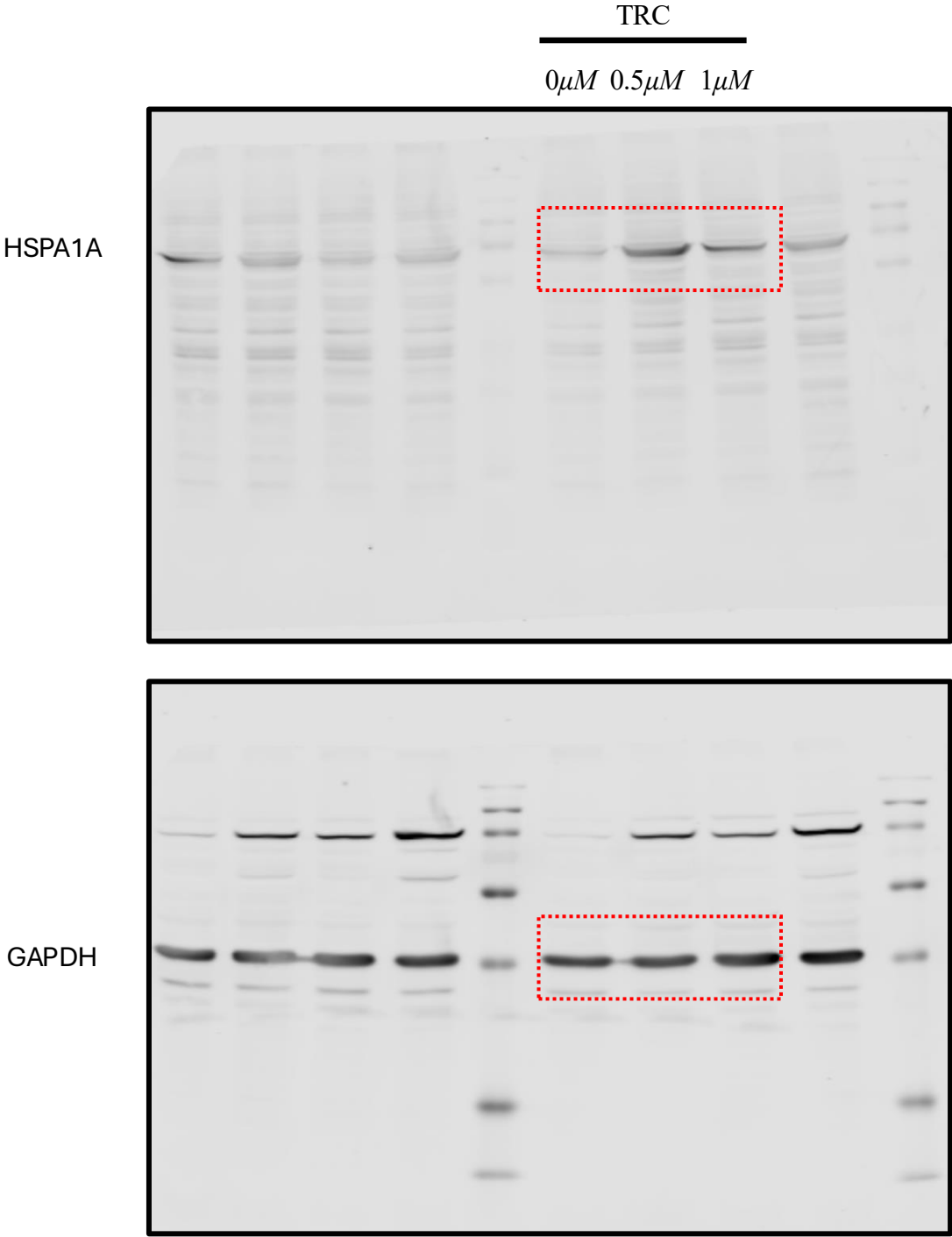

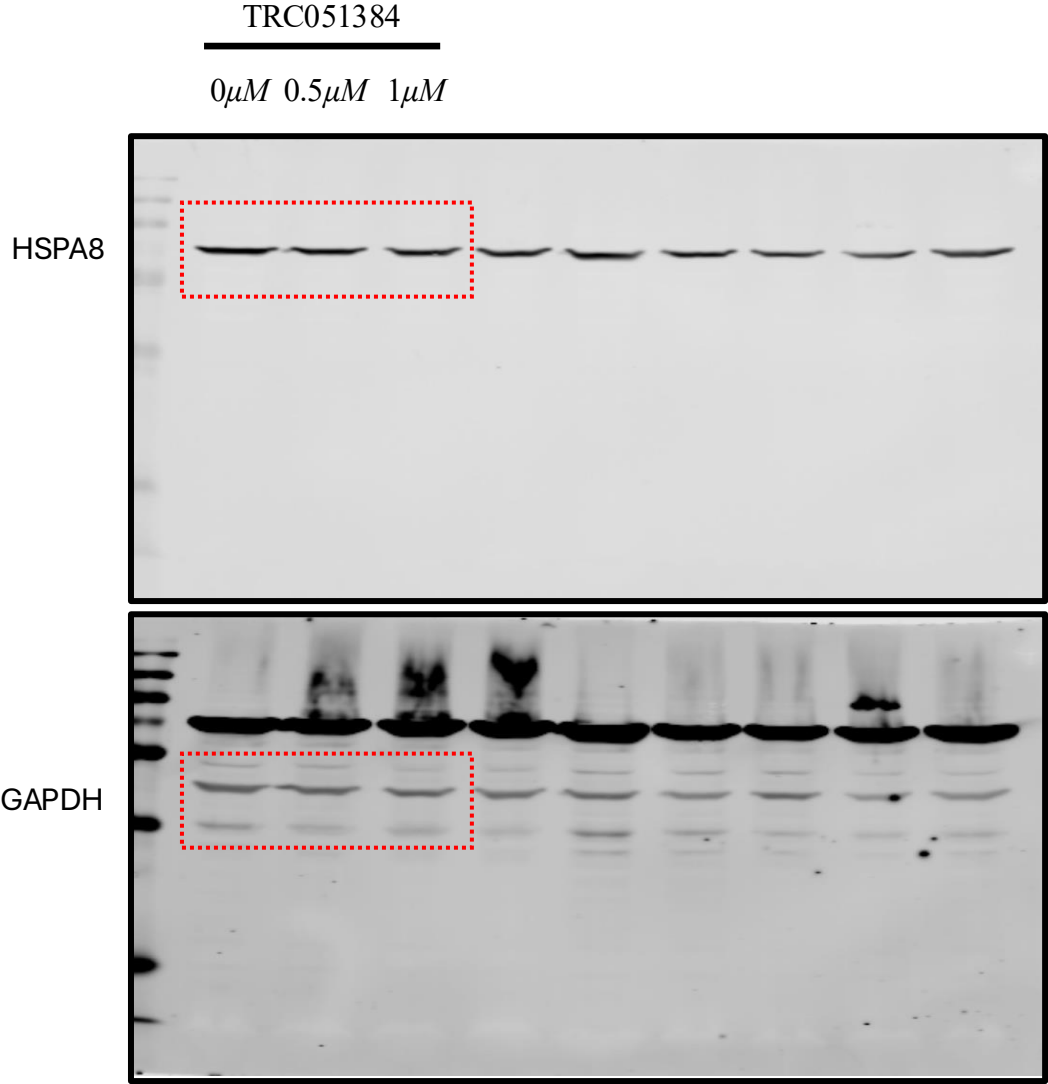

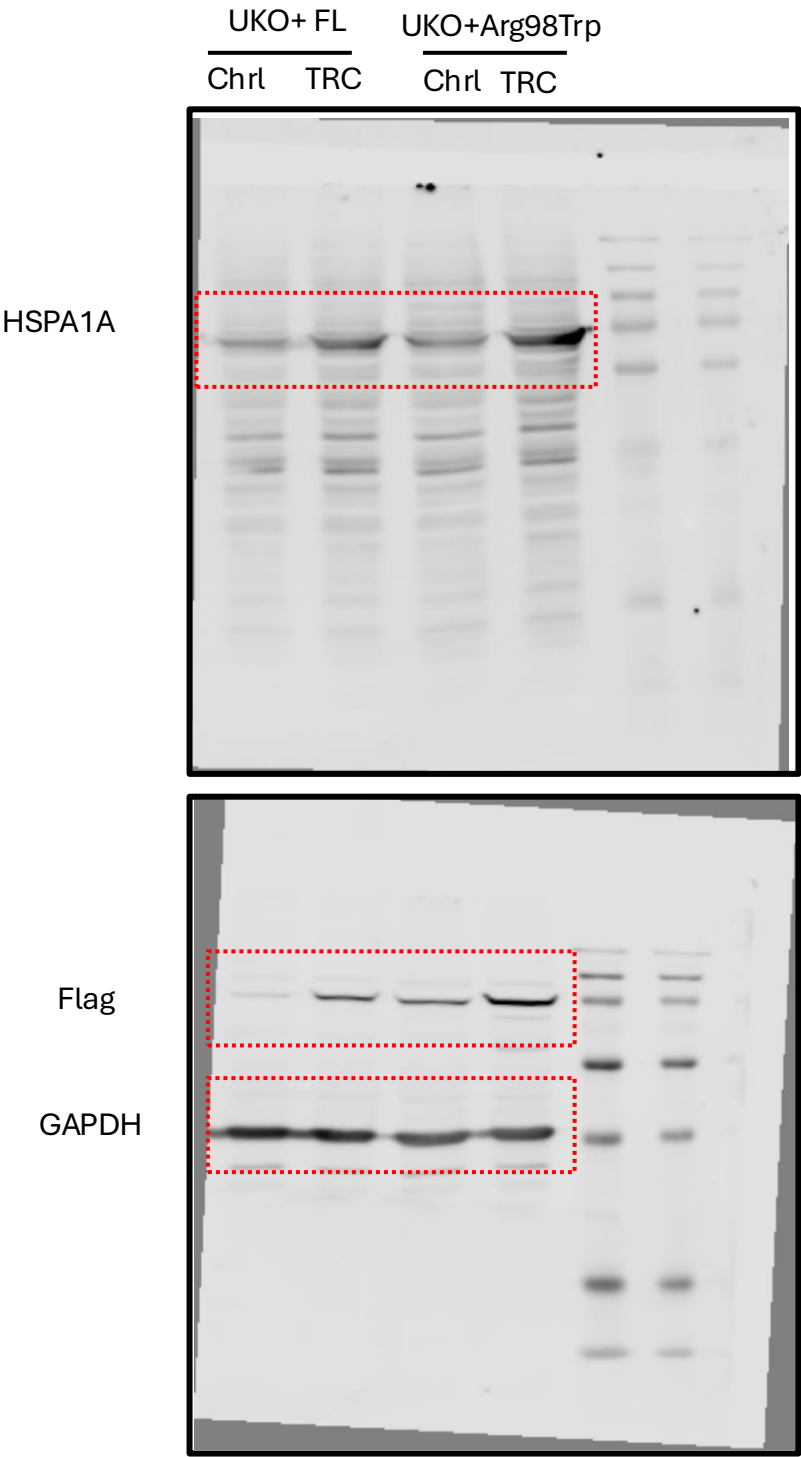

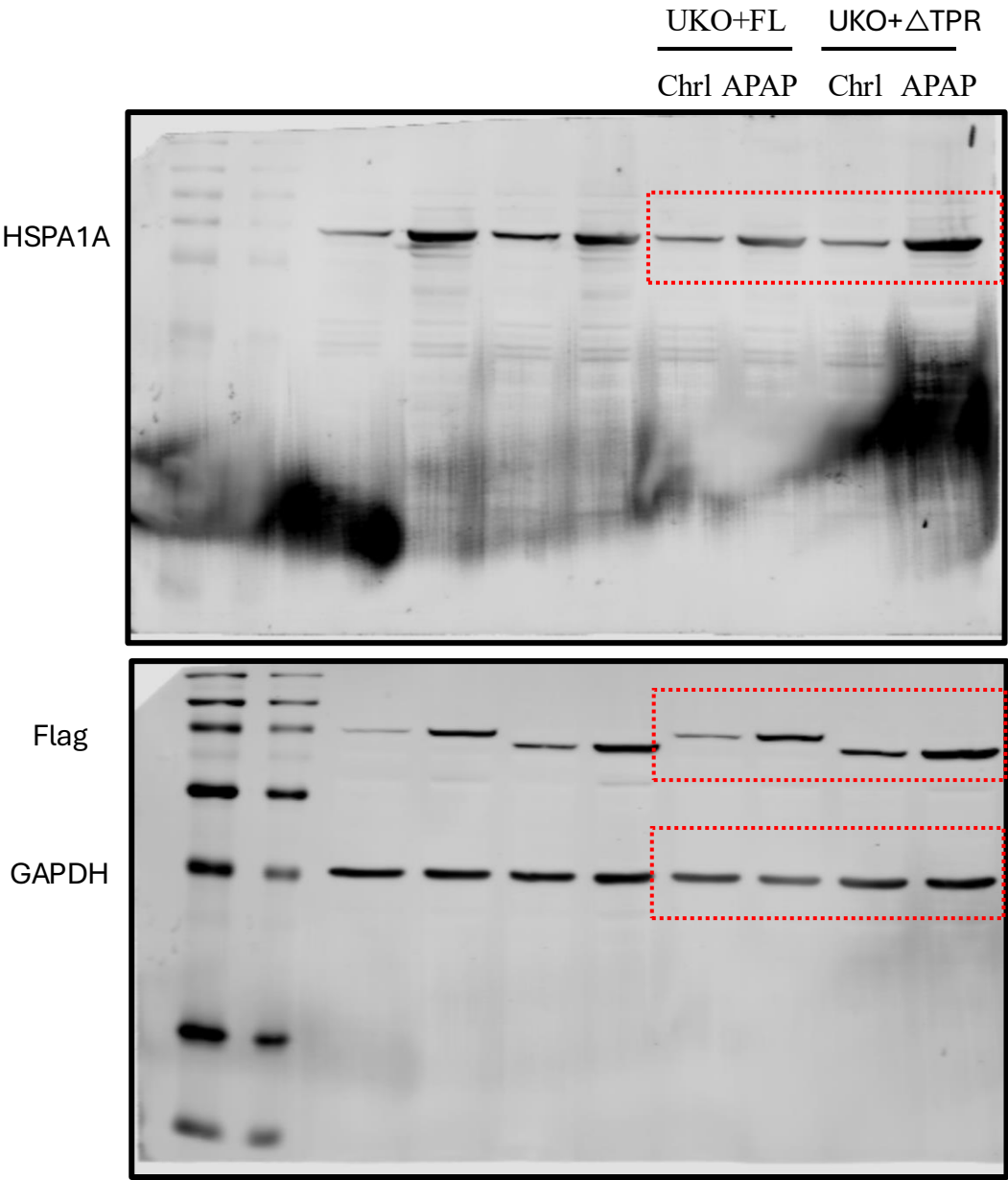

Supplement: WB Original Data [file mmc2.pdf]
