# Supplementary material for: A Molecular Mechanism of Intrahepatic Cholestasis in Osteo-Oto-Hepato-Enteric Syndrome
Source: Cell Mol Gastroenterol Hepatol. 2026 May 20;20(9):101805. doi: 10.1016/j.jcmgh.2026.101805 (PMC13324508; doi:10.1016/j.jcmgh.2026.101805)

cmgh
CELLULAR AND MOLECULAR GASTROENTEROLOGY AND HEPATOLOGY

ORIGINAL RESEARCH

A Molecular Mechanism of Intrahepatic Cholestasis in Osteo-Oto-Hepato-Enteric Syndrome

Zhe Zhou,[1] Yuan Lin,[1] Floris Imhann,[2,3] Henkjan J. Verkade,[4] Qinghong Li,[1,§] and Sven C. D. van IJzendoorn[1,§]

[1]Department of Biomedical Sciences, University of Groningen, University Medical Center Groningen, Groningen, the Netherlands; [2]Department of Gastroenterology and Hepatology, University of Groningen, University Medical Center Groningen, Groningen, the Netherlands; [3]Department of Genetics, University of Groningen, University Medical Center Groningen, Groningen, the Netherlands; and [4]Division of Pediatric Gastroenterology and Hepatology, Department of Pediatrics, University of Groningen, University Medical Center Groningen, Groningen, the Netherlands

A molecular mechanism ofintrahepatic cholestasis in osteo-oto-hepato-enteric syndrome

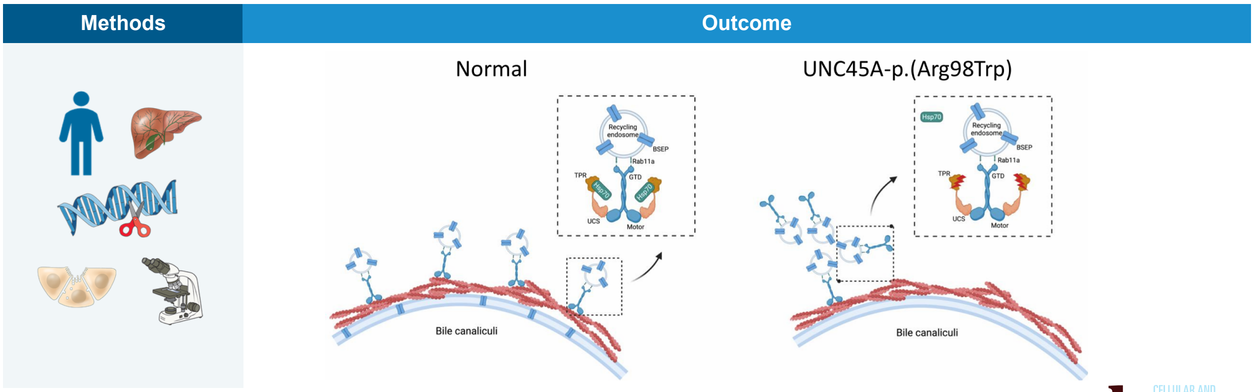

Summary O2HE with MYO5B-associated cholestasis are connected and HSP70-targeted therapies are suggested to restore canalicular BSEP expression in patient with the p.(Arg98Trp) variant.

SUMMARY

This study identifies uncoordinated 45A as a chaperone of MYO5B required for canalicular bile salt export pump trafficking and shows that an osteo-oto-hepato-enteric syndrome–associated uncoordinated 45A variant disrupts this process via impaired heat shock protein 70 interaction, suggesting a therapeutic strategy for cholestasis in this syndrome.

WHAT YOU NEED TO KNOW

Background: Cholestasis is a hallmark of the rare osteo-oto-hepato-enteric syndrome, but its causal relationship with the associated *UNC45A* gene and molecular underpinnings are not known.

Impact: The results connect osteo-oto-hepato-enteric syndrome with MYO5B-associated cholestasis and suggests heat shock protein 70–targeted therapies to restore canalicular bile salt export pump expression.

Future Directions: Development of a therapeutic strategy aimed at upregulating heat shock protein A1A expression in hepatocytes of patients carrying the c.292C>T (p.(Arg98Trp)) variant and explore other variant-specific mechanisms.

BACKGROUND & AIMS: Intrahepatic cholestasis is a hallmark of the rare osteo-oto-hepato-enteric syndrome. Osteo-oto-hepato-enteric syndrome is caused by pathogenic variants in *uncoordinated* (*UNC*) *45A. UNC45A* encodes a cochaperone that coordinates the proper folding and function of target proteins. The molecular basis of cholestasis in osteo-oto-hepato-enteric syndrome has not been demonstrated. Notably, the clinical phenotype overlaps with other familial cholestatic disorders involving impaired localization of the bile salt export pump (*ABCB11*).

METHODS: To investigate a mechanistic link between *UNC45A* dysfunction and bile salt export pump localization, we used immunofluorescence microscopy on liver biopsies from patients, CRISPR-CRISPR-associated protein 9–engineered male hepatocyte cell lines, site-directed mutagenesis, and coimmunoprecipitation assays. We focused on the recurrent c.292C>T/p.(Arg98Trp) variant.

RESULTS: Liver tissue of a patient carrying the UNC45A-c.292C>T/p.(Arg98Trp) variant displayed a zonated canalicular bile salt export pump expression defect. MYO5B, a known regulator of canalicular trafficking, was identified as an

UNC45A client. UNC45A–p.(Arg98Trp) caused bile salt export pump to accumulate in MYO5B+/RAB11A+ endosomes that failed to reach the canalicular membrane. Mutant UNC45A showed a weakened interaction with heat shock protein (HSP) 70 chaperones. Of these, HSPA1A showed a zonated expression in the liver, inversely correlating with the zonated bile salt export pump defect in the patient. Paracetamol-mediated increase in heat shock protein A1A abundance restored MYO5B+/RAB11A+ endosome positioning and canalicular bile salt export pump localization.

CONCLUSIONS: A weakened UNC45A–p.(Arg98Trp)–HSP70 interaction disrupts bile salt export pump trafficking through impaired MYO5B function and zonated HSPA1A availability. These results connect osteo-oto-hepato-enteric syndrome with MYO5B-associated cholestasis and suggests HSP 70–targeted therapies to restore canalicular bile salt export pump expression. *(Cell Mol Gastroenterol Hepatol 2026;20:101805; https://doi.org/10.1016/j.jcmgh.2026.101805)*

Keywords: Chaperone; Hepatocytes; HSPA1A; HSP70; Recycling Endosomes.

Cholestasis is a hallmark of osteo-oto-hepato-enteric (O2HE) syndrome.[1] The cholestasis manifests as neonatal icterus and intractable pruritus, associated with elevated bilirubin, aminotransferases, and serum bile acids,[1] and variable gamma-glutamyltransferase values.[1,2] Patients with O2HE additionally can present bone fragility, hearing loss, and/or diarrhea. Sex and/or gender differences are not expected. Treatment includes relief of cholestasis-associated pruritus by medication and/or surgical intervention.[1]

O2HE syndrome is an autosomal recessive disorder caused by biallelic variants in the *uncoordinated 45A* (*UNC45A*) gene.[1] Causality between *UNC45A* variants and cholestasis has not been demonstrated in animal or cell models but is supported by patients diagnosed with *UNC45A* variant-associated cholestasis-lymphedema syndrome (CLS). Patients with CLS present congenital intrahepatic cholestasis and lower-limb lymphedemas, but no deafness, bone fragility, or diarrhea.[3] CLS is distinct from O2HE syndrome in that the former always includes a variant in the nonprotein-coding 5′untranslated region of *UNC45A*.[3] Patients with O2HE syndrome have biallelic variants in protein-coding *UNC45A* regions.[1] *UNC45A* variants have been correlated with reductions in UNC45A protein levels.[1] The molecular mechanism underlying *UNC45A*-mediated cholestasis is not known.

Cholestasis in patients with O2HE syndrome shows similar clinical presentation and serum biochemistry as cases of familial cholestasis caused by variants in *ABCB11*, which encodes the bile salt export pump (BSEP)[4] or variants in the *MYO5B*-encoded molecular motor protein MYO5B.[5,6] BSEP is the main canalicular efflux system for bile acids,[7] and MYO5B mediates the intracellular trafficking of rat sarcoma virus-like protein from rat brain 11A (RAB11A)+ endosomes that carry BSEP to the canalicular surface.[4,8] UNC45A encodes a cochaperone protein that assists heat shock protein (HSP)70 and HSP90 in folding and stabilizing myosin proteins. We and others have implicated UNC45A as a cochaperone for MYO5B in intestinal cells.[9,10] We can therefore hypothesize that *UNC45A* variants caused cholestasis via disruptive effects on BSEP-carrying endosomes through interference with MYO5B folding. However, this hypothesis remains untested, and the mechanism of UNC45A mutant–mediated MYO5B dysfunction is unknown.

UNC45A contains 4 domains, a central and neck domain that form a backbone and orient 2 functional domains of the protein: the amino-terminal tetratricopeptide repeat (TPR) domain, and the carboxyl-terminal UCS (for UNC-45/Cro1/She4) domain.[11] The evolutionary conserved UCS domain interacts with myosin motor domains.[11,12] The TPR domain interacts with chaperone proteins HSP90/HSP70.[11] HSP70 has been reported to colocalize with BSEP in subcanalicular vesicles in rat hepatocytes.[13] Notably, the pathogenic variant in the TPR domain, p.(arginine [Arg]98tryptophane [Trp]) is close to the HSP90/70 binding site. The p.(Arg98Trp) variant has been reported in several patients, each in compound heterozygosity with a second pathogenic UNC45A allele. In 1 patient, who was treated with ursodeoxycholic acid, fat-soluble vitamins, glycyrrhizin, and ademetionine 1,4-butanedisulfonate, p.(Arg98Trp) was present in trans with an in-frame deletion in the UCS domain.[2] In a second patient, for whom treatment was not specified, p.(Arg98Trp) was compound heterozygous with a missense variant in the central domain.[1] In a third patient receiving odevixibat, p.(Arg98Trp) was identified in trans with a nonsense variant affecting the central domain.[14]

The aim of this study was to determine the mechanism underlying p.(Arg98Trp)-associated cholestasis in O2HE syndrome, focusing on MYO5B and BSEP.

§Authors share co-senior authorship.

Abbreviations used in this paper: APAP, N-acetyl-para-aminophenol; Arg, arginine; ASBT, apical sodium-dependent bile acid transporter; BC, bile canaliculus; BSEP, bile salt export pump; CLS, cholestasis-lymphedema syndrome; co-IP, coimmunoprecipitation; CV, coefficient of variation; EGFP, enhanced green fluorescent protein; HSP, heat shock protein; HSPA1A, heat shock 70 kDa protein 1; IF, immunofluorescence; IP, immunoprecipitation; MRP2, multidrug resistance protein 2; O2HE, osteo-oto-hepato-enteric; PBS, phosphate-buffered saline; PVDF, polyvinylidene difluoride; RAB11A, rat sarcoma virus-like protein from rat brain 11A; ROI, region of interest; SDS-PAGE, sodium dodecyl sulfate–polyacrylamide gel electrophoresis; TPR, tetratricopeptide repeat; Trp, tryptophane; UCS, UNC45/Cro1/She4; UKO, UNC45A knockout; UNC45A, uncoordinated 45A; WB, Western blot; WT, wild-type.

Most current article

© 2026 The Author(s). Published by Elsevier Inc. on behalf of American Gastroenterological Association Institute. This is an open access article under the CC BY license (http://creativecommons.org/licenses/by/4.0/).
2352-345X
https://doi.org/10.1016/j.jcmgh.2026.101805

Results

A Zonated Defect in Bile Salt Export Pump Expression in the Liver of a Patient With Osteo-Oto-Hepato-Enteric Syndrome

We recently reported a patient with O2HE syndrome suffering from recurring episodes of low–gamma-glutamyltransferase cholestasis and pruritus, carrying biallelic genetic variants in *UNC45A*: a missense variant c.292C>T (p.(Arg98Trp)) in the TPR domain) and a nonsense variant c.592C>T p.(Gln198*).[14] Immunolabeling of BSEP in a liver biopsy of this patient with O2HE revealed aberrant BSEP expression only in midacinar hepatocytes (Figure 1*A, G,* and *H*). Boosting the fluorescence signal suggested that BSEP was abnormally localized to punctate structures in the cytoplasm of the hepatocytes (Figure 1*H*). By contrast, BSEP expression and localization appeared normal at the canalicular surface of hepatocytes adjacent to vascular structures (Figure 1*G*). In control liver, BSEP was equally expressed at the canalicular surface of hepatocytes in both midacinar and perivascular regions (Figure 1*E* and *F*), consistent with earlier reports.[15] UNC45A in patient liver, when compared with control liver (Figure 1*A* and *B*), showed reduced expression but was still present in the midacinar region (Figure 1*C* and *D*).

UNC45A Controls MYO5B Expression

In hepatocytes in vivo,[4] as well as in hepatocellular cell lines,[16] BSEP is carried to the bile canalicular membrane in RAB11A+ endosomes under control of MYO5B. Because UNC45A is a myosin-directed cochaperone, we focused on MYO5B as a potential target of UNC45A in hepatocytes, and on RAB11A and BSEP distribution as a functional readout for MYO5B function. We have generated UNC45A knockout HepG2 cells, hereafter referred to as HepG2-UNC45A knockout (UKO).[9] We transfected an enhanced green fluorescent protein (EGFP)-tagged BSEP, which traffics similarly as endogenous BSEP,[12] into these cells. Western blot (WB) analysis demonstrated the complete depletion of the UNC45A protein in HepG2-UKO cells (Figure 2*A* and *B*). Wild-type (WT) HepG2 cells showed an evenly distributed pericanalicular distribution of MYO5B+/RAB11A+ endosomes and a canalicular distribution of BSEP (Figure 2*C*), similar to that seen in human liver tissue.

HepG2-UKO cells showed a similar pericanalicular distribution of RAB11A+ endosomes and canalicular distribution of BSEP (Figure 2*D*). However, HepG2-UKO cells showed a severely reduced expression level of MYO5B, when compared with WT HepG2 cells, as evidenced by WB analysis (Figure 2*A* and *B*) and immunofluorescence (IF) microscopy (Figure 2*D*). The loss of MYO5B expression in HepG2-MYO5B KO cells (Figure 2*A* and *B*) confirmed the specificity of the antibody. Re-expression of full-length UNC45A genetically tagged with a carboxyl-terminal DYKDDDDK (FLAG) tag in HepG2-UKO cells (UNC45A-UKO+FL) preserved MYO5B expression (Figure 2*A* and *B*), which displayed a normal pericanalicular distribution (Figure 2*E*; cf, Figure 2*C*), hence demonstrating that MYO5B is a target of UNC45A.

These data show that the loss of UNC45A causes the loss of MYO5B, without affecting RAB11A+ endosome or BSEP localization. This is in agreement with observations made in cell lines and mice that, in contrast to the expression of dysfunctional myosin variants, the mere loss of MYO5B did not affect BSEP and/or RAB11A+ endosomes and did not cause cholestasis, likely due to unknown compensatory mechanisms.[8,17]

The UNC45A Tetratricopeptide Repeat Domain Is Required for MYO5B Function

We next reintroduced specific domains or truncated forms of UNC45A in HepG2-UKO cells. Expression of the UCS domain in HepG2-UKO cells (HepG2-UKO+UCS) was sufficient to maintain MYO5B expression (Figure 3*A*). However, expression of the UCS domain, in contrast to the expression of the full-length UNC45A (cf, Figure 2*E*), led to the aberrant distribution of MYO5B and of RAB11A+ endosomes throughout the cytoplasm (Figure 3*B*). Similarly, expression of UNC45A without its TPR domain in HepG2-UKO cells (HepG2-UKO+ΔTPR) maintained MYO5B expression but perturbed the pericanalicular distribution of MYO5B and of RAB11A+ endosomes (Figure 3*A* and *B*).

This demonstrates that the UCS domain is sufficient to stabilize MYO5B expression but that the TPR domain of UNC45A is required to maintain MYO5B function towards RAB11A+ endosomes.

UNC45A.p(Arg98Trp) Causes Accumulation of Bile Salt Export Pump in Aberrantly Positioned RAB11A+ Endosomes

Expression of UNC45A-p.(Arg98Trp) (Figure 4*A*) in HepG2-UKO cells preserved the expression of MYO5B but perturbed its pericanalicular distribution. MYO5B accumulated at a discrete region at or close to the canalicular membrane (Figure 4*B*, *arrow*) and failed to acquire a uniform pericanalicular distribution (Figure 4*B*). Consistent with the role of MYO5B as a regulator of RAB11A+ endosome trafficking, RAB11A+ endosomes accumulated with MYO5B at this juxtacanalicular region in UNC45A-p.(Arg98Trp)-expressing cells (Figure 4*C*, *arrow*).

Because variation in MYO5B/RAB11A distribution at the canalicular membrane was observed between individual canaliculi, we developed an objective measure, the coefficient of variation (CV), to quantify the extent of uniform, pericanalicular distribution of MYO5B and RAB11A+ endosomes (see Methods and Figure 5). Expression of UNC45A-p.(Arg98Trp) in HepG2-UKO cells led to a significant increase in CV for both MYO5B and RAB11A fluorescence (Figure 4*D*), indicative for less uniformity in fluorescence distribution along the canalicular perimeter.

Because MYO5B dysfunction has been reported to disrupt the trafficking of BSEP via MYO5B+/RAB11A+ endosomes to the canalicular surface,[11] we investigated the localization of BSEP in UNC45A-p.(Arg98Trp)-expressing cells and found that EGFP-BSEP distribution was altered and colocalized with the aberrantly localized MYO5B+/RAB11A+ endosomes in the juxtacanalicular region

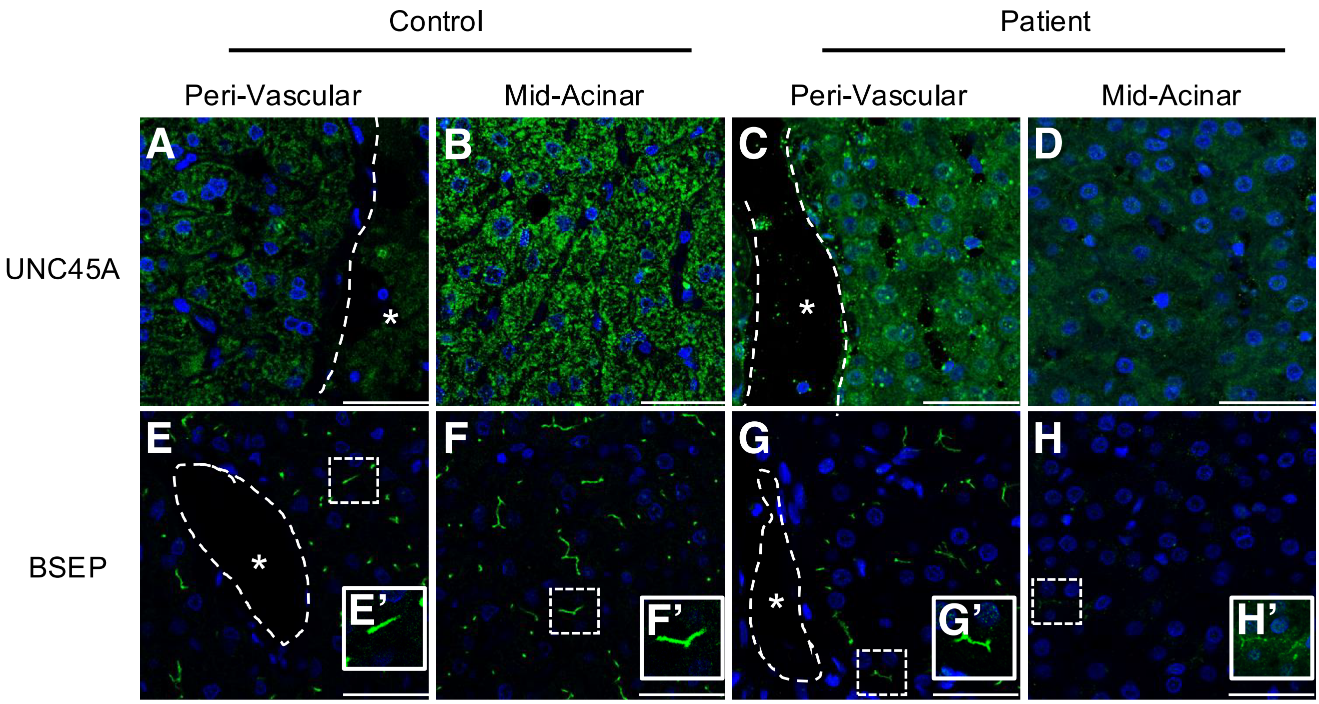

Figure 1. Immunolabeling of UNC45A variant and BSEP in O2HE patient and control liver tissue. *Inserts in the lower panels* show enlargement of the boxed area in the same panel. *Asterisks* mark vasculature. Scale bar, 20 μm.

(Figure 4*E*, *arrow*). The canalicular distribution of multidrug resistance protein 2 (MRP2) appeared unaltered (Figure 6), in agreement with reports of MRP2 immunohistochemistry in liver tissue of patients with O2HE syndrome.[1]

Together, the results indicate that the UNC45A variant led to dysfunctional MYO5B and consequent defects in the canalicular trafficking of BSEP-carrying RAB11A+ endosomes. We next investigated the underlying molecular mechanism.

UNC45A.p(Arg98Trp) Displays a Weakened Interaction With Heat Shock Protein 70

Arg98 locates in the TPR domain, close to the binding site for HSP70/90 chaperones[18] (Figure 7*A*). Our in silico analysis predicted that upon Arg98Trp substitution, the bulky side chain of Trp is likely incompatible with neighboring helices in the TPR domain, possibly affecting the HSP70/90 binding site.[19] We observed significant coimmunoprecipitation (co-IP) of a main HSP70 family member, HSPA1A, with full-length UNC45A-FLAG. As expected, no HSPA1A was coimmunoprecipitated with UNC45A-ΔTPR-FLAG (Figure 7*B*, quantification in Figure 7*C*), demonstrating the validity of the assay. When compared with full length UNC45A-FLAG, significantly less HSPA1A protein was coimmunoprecipitated with UNC45A-p.(Arg98Trp)-FLAG protein (Figure 7*B* and *C*). HSPA8, another HSP70 family member constitutively expressed in hepatocytes, also showed a partially disruptive interaction with UNC45A-p.(Arg98Trp) (Figure 7*B* and *C*). UNC45A-p.(Arg98Trp), when compared with WT UNC45A, also showed reduced interaction with MYO5B (Figure 7*B* and *C*). The lack of specificity towards different HSP70 family members is indicative for alterations in the HSP70-binding pocket.

These results demonstrate that Arg98Trp substitution in UNC45A leads to a weakened interaction with HSP70 and with MYO5B.

The UNC45A.p(Lys93Glu) Mutant and Pharmacologic Heat Shock Protein 70 Inhibition Phenocopy the UNC45A.p(Arg98Trp) Variant

If the reduced interaction of the UNC45A-p.(Arg98Trp) protein with HSP70 was responsible for the aberrant distribution of BSEP and MYO5B+/RAB11A+ endosomes, the expression of another HSP70 binding-disruptive UNC45A mutant in HepG2-UKO cells would be expected to phenocopy the effect of UNC45A-p.(Arg98Trp). Lys82 in the Hsp70 binding groove of the UNC45 TPR domain mediates binding to HSP70/90, as predicted from the crystal structure of the *Caenorhabditis elegans* UNC45/Hsp70 complex[18] and validated in vitro.[20] Lys82 is highly conserved and corresponds to Lys93 in human UNC45A. We generated UNC45A-p.Lys93Glu-FLAG (Figure 8*A*) which showed weakened interaction with HSPA1A when expressed in HepG2-UKO cells (Figure 8*B*). This mutant, contrasting the full-length UNC45A-FLAG but similar to the UNC45A-p.(Arg98Trp) mutant, perturbed the pericanalicular distribution of MYO5B+/RAB11A+ endosomes and resulted in the juxtacanalicular accumulation of MYO5B+/RAB11A+ endosomes (Figure 8*B*).

VER-155008, a specific inhibitor of HSP70 ATPase activity,[21] produced comparable results. Treatment of WT

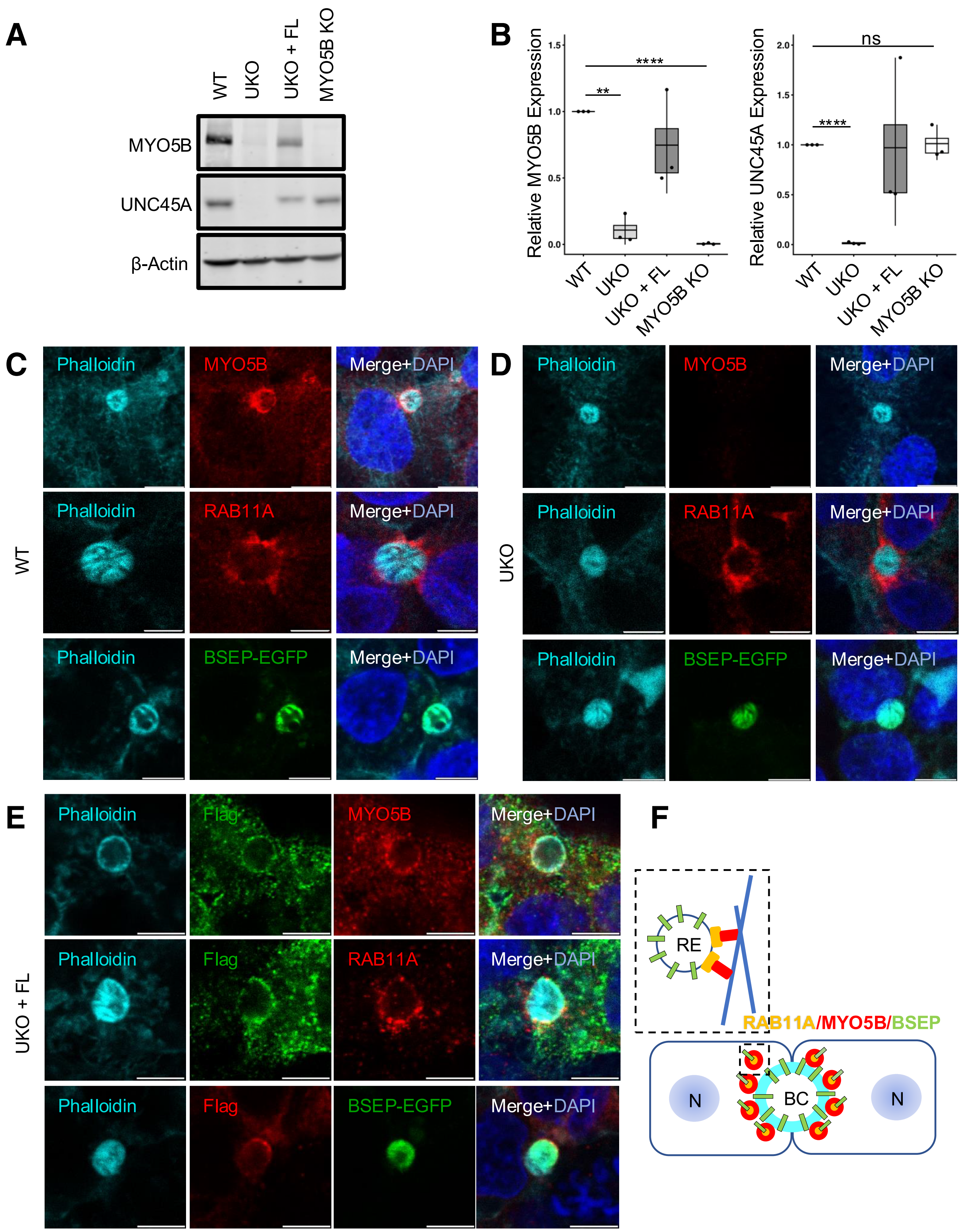
A
WT
UKO
UKO + FL
MYO5B KO
MYO5B
UNC45A
β-Actin
B
Relative MYO5B Expression
Relative UNC45A Expression
**
****
ns
C
D
E
F
WT
UKO
UKO + FL
Phalloidin
MYO5B
RAB11A
BSEP-EGFP
Flag
Merge+DAPI
RE
RAB11A/MYO5B/BSEP
N
BC

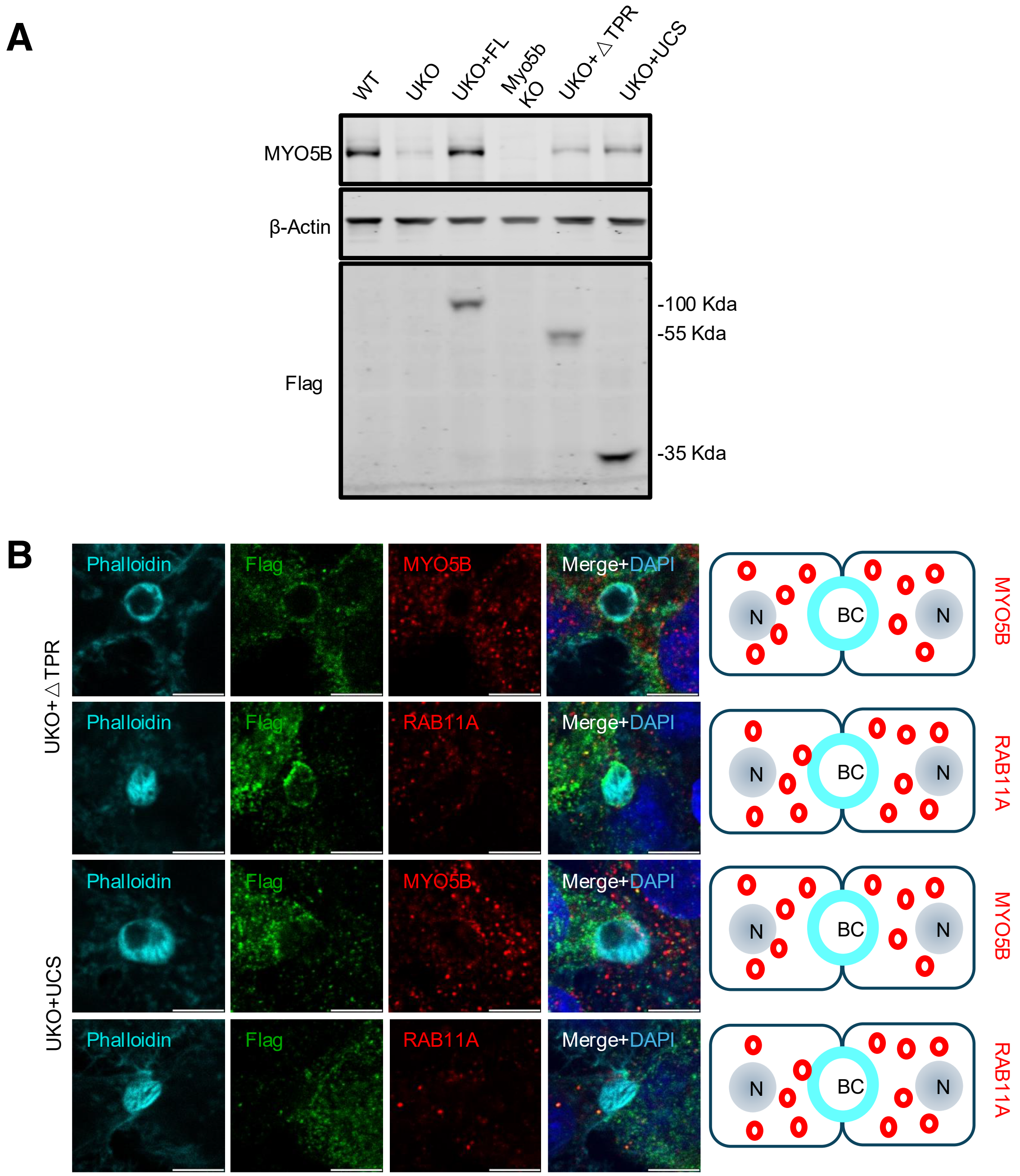

Figure 3. Role of the UNC45A UCS and TPR domains in the expression and localization of MYO5B and RAB11A. (*A*) Representative WB showing the expression of MYO5B and FLAG in HepG2-WT, HepG2-UKO, HepG2- UKO+FL, HepG2-MYO5B KO, HepG2-UKO+ΔTPR, and HepG2-UKO+UCS cells. (*B*) IF images showing the distribution of MYO5B and RAB11A in HepG2-UKO+ΔTPR and HepG2-UKO+UCS cells. Scale bar, 5 μm.

HepG2 cells with VER-155008 changed the distribution of MYO5B+/RAB11A+ endosomes and BSEP as a function of time from a pericanalicular distribution to their accumulation at juxtacanalicular spots (Figure 9*A*–*C*).

The collective genetic and pharmacologic results support that a weakened interaction with active HSP70 mediated the effect of UNC45A.p(Arg98Trp) on MYO5B and BSEP-carrying RAB11A+ endosomes.

Figure 2. (***See previous page***). **Effect of UNC45A deletion on MYO5B and RAB11A expression in HepG2 cells.** (*A*) Representative WBs showing the expression of MYO5B, UNC45A in WT, HepG2-UKO, HepG2-UKO-FL, and MYO5B KO HepG2 cells. (*B*) Quantification of the blots shown in panel (*A*). (*C*–*E*) IF images showing the (sub-) canalicular localization pattern of filamentous actin (phalloidin), MYO5B, RAB11A, and BSEP-EGFP in HepG2-WT (*C*), HepG2-UKO (*D*), and HepG2-UKO+FL (*E*) cells. (*F*) Schematic illustration of the key findings from this figure. Scale bars, 5 μm.

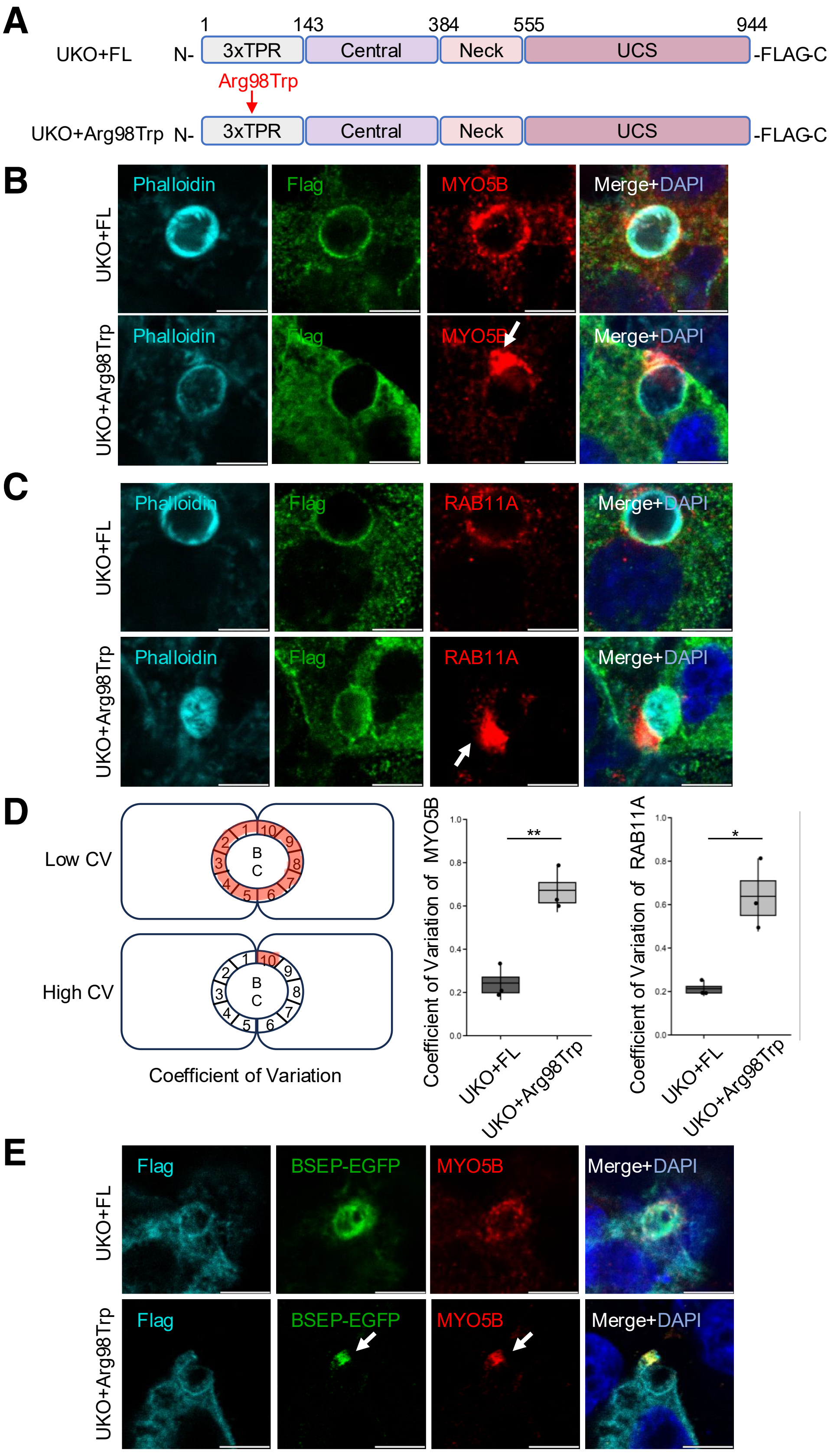

Figure 4. Effect of UNC45A variants on MYO5B and RAB11A expression in HepG2 cells. (*A*) Schematic illustration of the UNC45A constructs used. (*B, C,* and *E*) IF images showing the (sub-)canalicular localization pattern of filamentous actin (phalloidin; all), MYO5B (*B*), RAB11A (*C*), and BSEP-EGFP (*E*) in HepG2-UKO+FL and HepG2-UKO+R98W cells. Scale bars, 5 μm. (*D*) Quantification of the coefficient of variation shown in panels (*B, C,* and *E*). Scale bars, 5 μm.

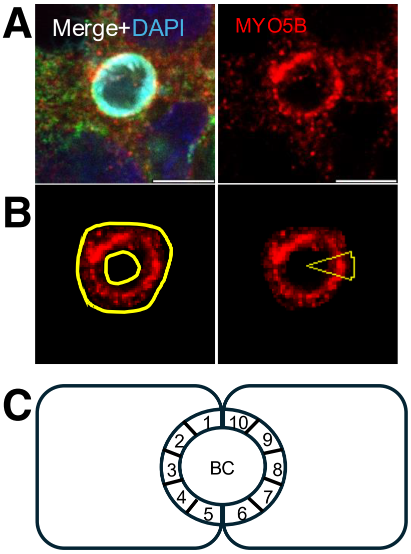

D

$$SD = \sqrt{\frac{1}{n-1}\sum_{k=1}^{n}(I_k - \bar{I})^2}$$

$$CV(\%) = \frac{SD}{\bar{I}} \times 100$$

E

Sector	BC1	BC2	BC3	...
1	X	X	X	...
2	X	X	X	...
...	X	X	X	...
10	X	X	X	...
Mean	X	X	X	...
SD	X	X	X	...
CV(%)	X	X	X	...

Figure 5. Explanation of the analyses of CV. (*A* and *B*) An ROI is drawn at the BC perimeter. (*C*) The ROI is divided into equal sectors, and fluorescence intensity is measured in each sector. (*D*) Formula for calculation of CVs. (*E*) Schematic of table used to calculate CV values.

Increasing HSPA1A Abundance Rescues the Phenotype in UNC45A-p.(Arg98Trp)-Expressing Cells

The main cytosolic HSP70 family members in hepatocytes are HSPA8 and HSPA1A (which is constitutively expressed in normal cells but can be rapidly upregulated). In contrast to HSPA8, HSPA1A displays a zonated expression in the liver parenchyma (www.proteinatlas.org). A relatively low expression of HSPA1A is seen in the mid-acinar region, which is the region in the patient liver where the reduced expression of the mutant UNC45A protein and loss of canalicular BSEP was observed. By contrast, a relatively high expression of HSPA1A is seen in the perivascular areas (Figure 10), which is the region in the patient liver where a higher expression of the mutant UNC45A protein and a normal BSEP staining was observed (cf, Figure 1).

This led us to hypothesize that a lower HSPA1A availability in midacinar hepatocytes expressing the mutant UNC45A-p.(Arg98Trp) may have rendered the cells more susceptible to MYO5B dysfunction and, subsequently, aberrant RAB11A-endosome distribution and defective BSEP delivery to the canalicular surface. By contrast, a higher abundance of HSPA1A in perivascular hepatocytes may have compensated for the weakened interaction of UNC45A-p.(Arg98Trp) with HSP70 by increasing the likelihood of interaction events. We therefore determined whether increasing HSPA1A protein

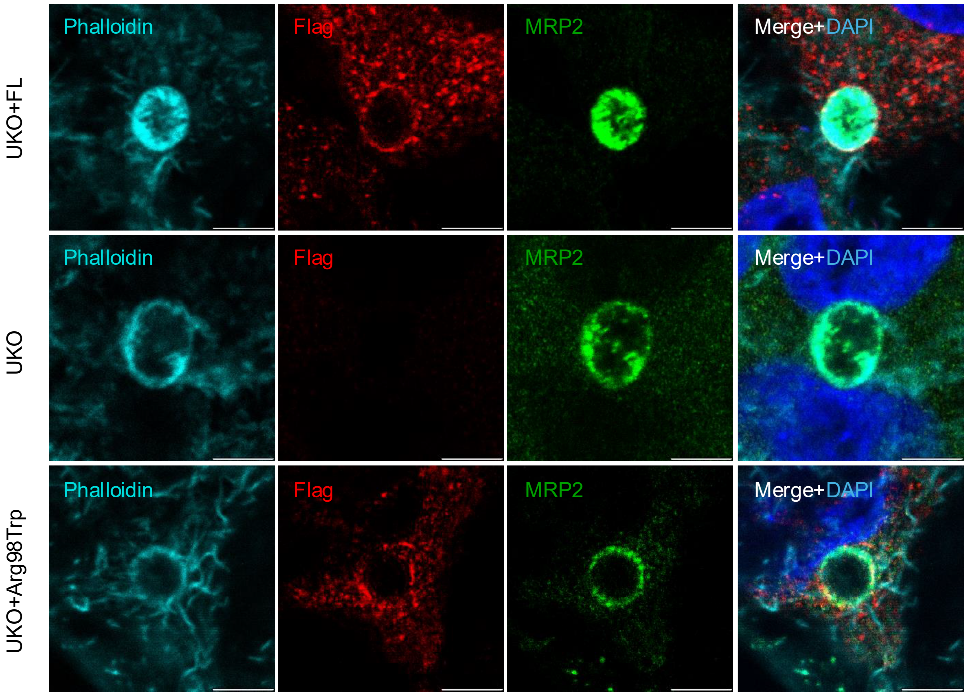

Figure 6. IF images showing the distribution of MRP2 in HepG2-UKO and HepG2-UKO+UKO+Arg98Trp cells. Scale bar, 5 μm.

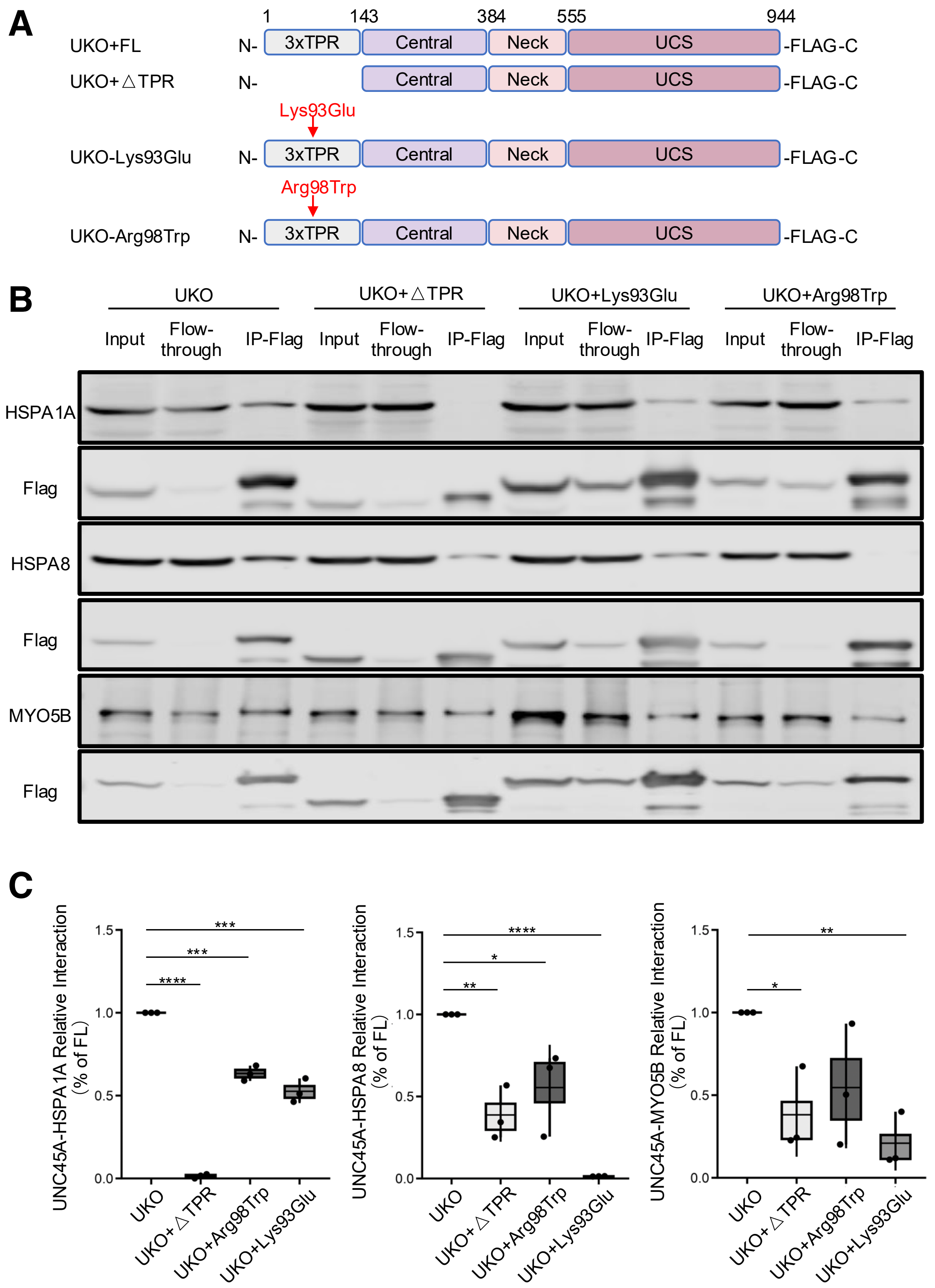

Figure 7. Effect of various UNC45A variants on their interaction with HSPA1A, HSPA8, and MYO5B in HepG2 cells. (*A*) Schematic illustration of the UNC45A constructs used. (*B*) WBs (representative of at least 3 independent experiments) showing the coIP results (see main text for explanation). (*C*) Quantification of the WB results depicted in panel (*B*).

abundance in cells expressing UNC45A-p.(Arg98Trp) could rescue the BSEP and MYO5B+/RAB11A+ endosome phenotype.

The classical way to increase the expression of HSPA1A is heat shock. Exposing HepG2 cells expressing UNC45A-p.(Arg98Trp) to heat shock (see Methods) led to a time-

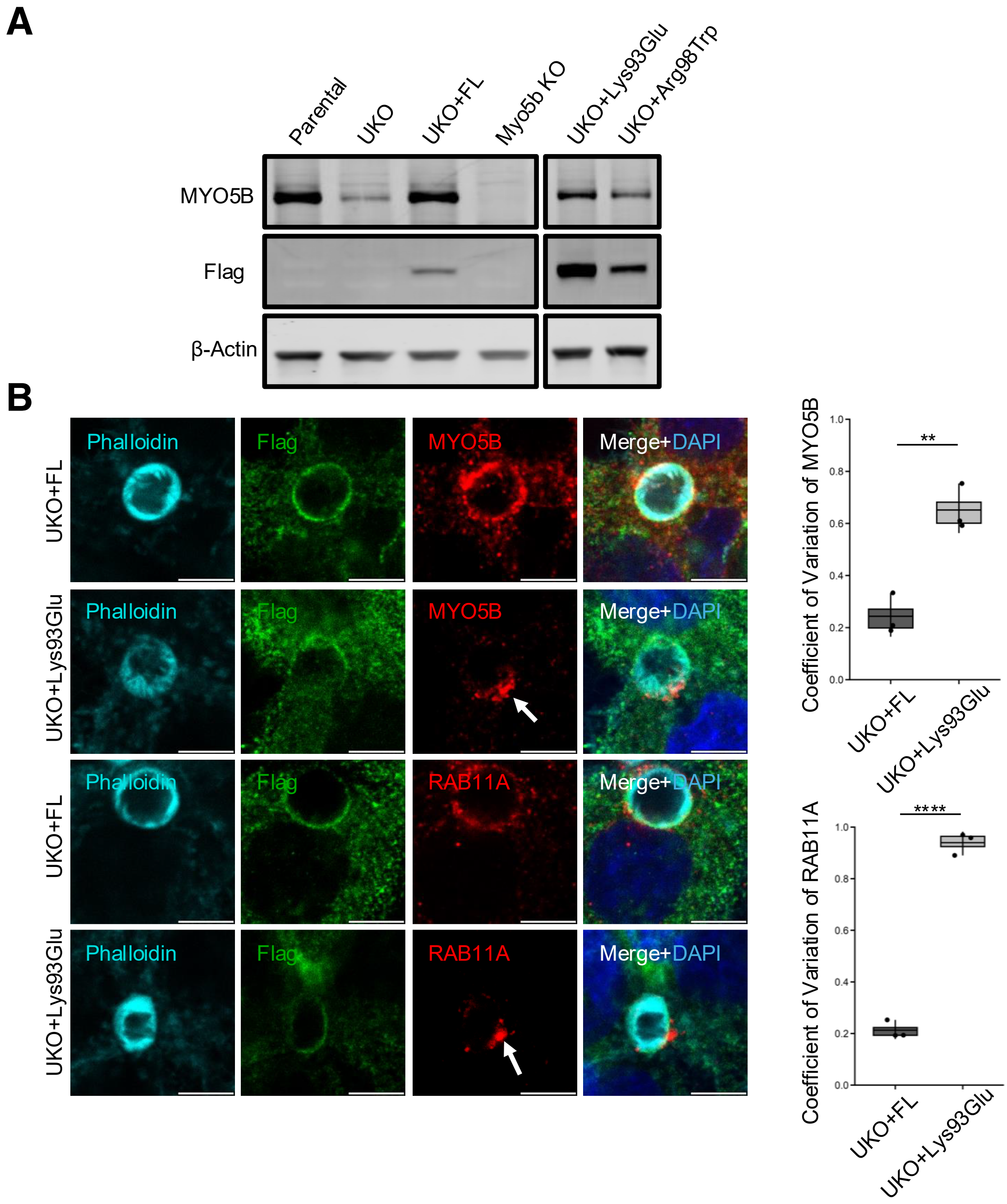

Figure 8. Effect of the UNC45A-K93E mutant on the expression and localization of MYO5B and RAB11A. (*A*) Representative WB showing the expression of MYO5B and FLAG in HepG2-WT, HepG2-UKO, HepG2-UKO+FL, HepG2-MYO5B KO, HepG2-UKO+K93E, and HepG2-UKO+R98W cells. (*B*) IF images showing the distribution of MYO5B and RAB11A in HepG2-UKO+FL and HepG2-UKO+R93W cells. Phalloidin and DAPI are used to stain the BC-enriched F-actin cytoskeleton and nuclei, respectively. Scale bar, 5 μm. Quantification of the CVs of the IF images as shown in (*B*) are shown at the *right side*.

dependent increase in HSPA1A expression (Figure 11*A*). No such effect was observed for HSPA8 (Figure 11*A*). Heat exposure of HepG2 cells expressing UNC45A-p.(Arg98Trp) led to an increase in the expression level of the FLAG-tagged UNC45A-p.(Arg98Trp) variant by WB (Figure 11*B*) and, importantly, a near-complete rescue of the BSEP and MYO5B and RAB11A+ endosome phenotype, yielding uniformly distributed MYO5B and RAB11A+ endosomes and BSEP at the canalicular plasma membrane (Figure 12*A*–*C*).

Also, drugs can increase HSPA1A expression. Treatment of HepG2 cells expressing UNC45A-p.Arg98Trp with TRC051384 ($C_{25}H_{31}N_5O_4$)[22] (Figure 13*A*) or the generic, over-the-counter acetaminophen (APAP; also known as

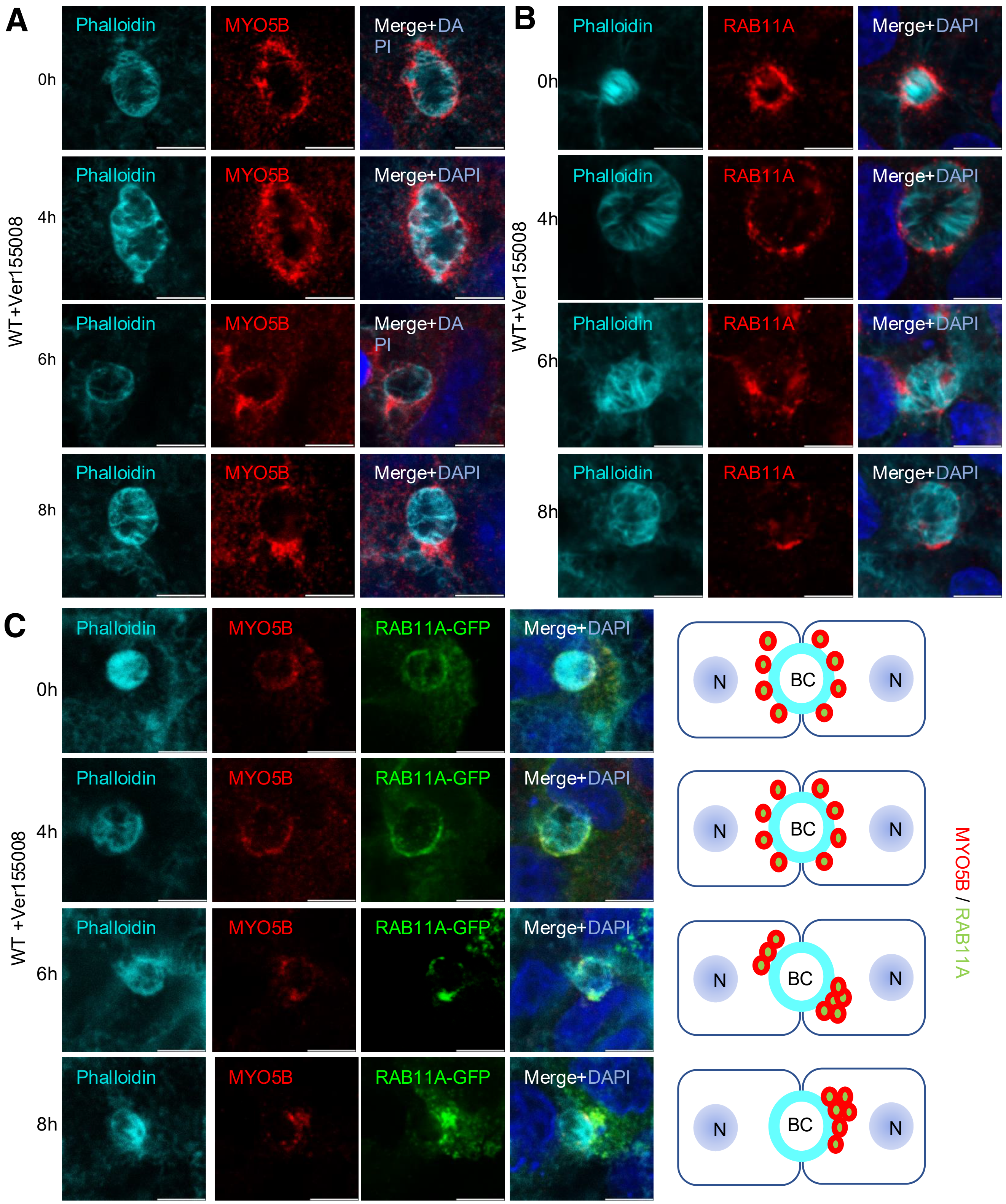

Figure 9. Effect of HSP70 inhibition on MYO5B and RAB11A distribution in HepG2 cells. (*A–C*) Fluorescence microscopy images showing the change in distribution of Myo5b (*A*), Rab11a (*B*), and colocalization of MYO5B and RAB11A (*C*) upon treatment with the HSP70 inhibitor VER-155008 as a function of time. Phalloidin and DAPI are used to stain the BC-enriched F-actin cytoskeleton and nuclei, respectively. Scale bar, 5 μm.

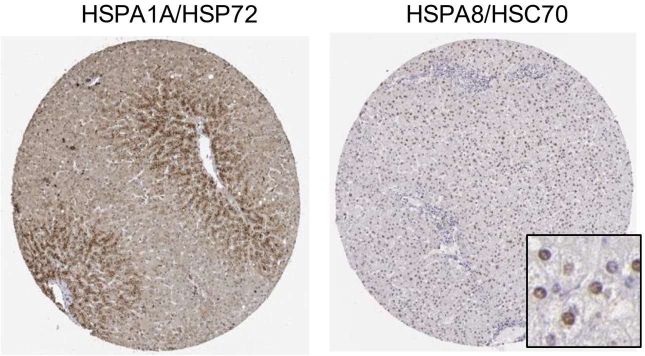

Figure 10. Expression of the different HSP70 family members in human liver tissue. Note the zonated expression of HSPA1A. Images are taken from https://www.proteinatlas.org.

paracetamol or Tylenol)[23] (Figure 14*A*), led to a significant increase in the expression of HSPA1A, but not of HSPA8 (Figure 14*A*). Fluorescence microscopy showed that treatment with either TRC051384 (Figure 15) or APAP (Figure 14*B*) effectively rescued the UNC45A-p.(Arg98Trp)-induced phenotype, resulting in an increase in the expression of the mutant UNC45A protein (Figure 14*B*) and a normal pericanalicular distribution of MYO5B and RAB11A-positive endosomes and BSEP (Figure 16).

To determine the relationship between the expression level of the mutant UNC45A protein and the BSEP phenotype, we performed these experiments also in HepG2 cells expressing the HSP70 binding-incapable UNC45A-ΔTPR-FLAG. All treatments increased the expression of the UNC45A-ΔTPR-FLAG (Figure 17), but none of these treatments rescued the canalicular MYO5B/RAB11A/BSEP phenotype in HepG2 cells that expressed UNC45A-ΔTPR-FLAG (Figure 17). This demonstrates that the rescuing effect of these treatments on the function of MYO5B and canalicular localization of BSEP was mediated by the TPR domain of UNC45A and uncoupled from mutant UNC45A protein expression level as such.

Discussion

This study establishes the causal and molecular link between the c.292C>T (p.(Arg98Trp)) variant in *UNC45A* and intrahepatic cholestasis in O2HE syndrome. It shows that the p.(Arg98Trp) mutation disrupts the normal localization of BSEP, the key BSEP, in hepatocytes in patient liver biopsy and in genetically engineered hepatocellular cells. Mechanistically, this UNC45A variant displays weakened interaction with HSP70. This leads to perturbed MYO5B function, and failure of MYO5B-dependent RAB11A+ endosomes to reach the pericanalicular region and deliver BSEP to the canalicular surface. Hence, defective MYO5B protein quality control is responsible for impaired canalicular BSEP expression and cholestasis in patients with O2HE syndrome with the c.292C>T (p.(Arg98Trp)) variant.

The Difference Between the Loss of MYO5B Expression vs the Expression of Dysfunctional MYO5B

Complete loss of UNC45A caused a profound loss of MYO5B expression, establishing MYO5B as a UNC45A client, but complete loss of UNC45A did not cause defects in RAB11A endosomes or BSEP distribution. We previously demonstrated that the loss of MYO5B did not cause cholestasis, likely due to compensatory mechanisms.[17] In support, no patients with isolated intrahepatic cholestasis were identified with biallelic null variants in *MYO5B*.[5] Likewise, no patients with O2HE syndrome carrying biallelic null variants in *UNC45A* have been reported in the medical literature. In contrast to a loss of expression, the presence of missense MYO5B variants in hepatocytes did cause cholestatic phenotypes.[8,17] This implied that MYO5B-associated cholestasis arises from the presence of dysfunctional MYO5B variants that exert a gain-of-toxic function (ie, by aberrantly interfering with RAB11+ endosome trafficking).[8,17] Our data suggest that the HSP70 binding-disruptive UNC45A-p.(Arg98Trp) protein impaired MYO5B folding and its function as a motor protein for BSEP-carrying RAB11A+ endosomes, essentially mimicking motor-defective MYO5B variants in *MYO5B* variant-associated cholestasis.

A Zonated Bile Salt Export Pump Defect

In the patient's liver, the abnormal BSEP localization was confined to the midacinar region, a zonated defect not previously reported in other familial forms of cholestasis. Also in liver tissue from another patient with O2HE syndrome carrying compound heterozygous variants, a canalicular loss of BSEP mainly in midacinar hepatocytes was observed, whereas in liver tissue from her sibling and from another patient with other compound heterozygous variants, BSEP appeared normally distributed.[1] Another canalicular transporter, the *ABCC4*-encoded MRP2, appeared normally distributed in all patients with O2HE syndrome of which liver tissue was subjected to immunohistochemistry,[1] similar to our observations in cultured cells. The reduced midacinar expression level of the UNC45A variant in our patient, as such, does not appear to play a key role in the cholestatic phenotype as full knockout of UNC45A in the cell model was without effect. Our findings suggest that this regional phenotype in this patient results from the combination of: (1) the weakened interaction between UNC45A-p.(Arg98Trp) and HSP70; and (2) the relatively low midacinar expression of the HSP70 family member HSPA1A. Indeed, increasing HSPA1A abundance through heat shock or treatment with drugs restored the pericanalicular distribution of MYO5B+/RAB11A+ endosomes and normalized BSEP localization. These results support a scenario in which limited HSPA1A availability drives the zonated defect in BSEP trafficking and expression in the livers of cholestatic patients with O2HE syndrome carrying the UNC45A-p.(Arg98Trp) variant.

These results support a therapeutic strategy aimed at upregulating HSPA1A expression in midacinar hepatocytes

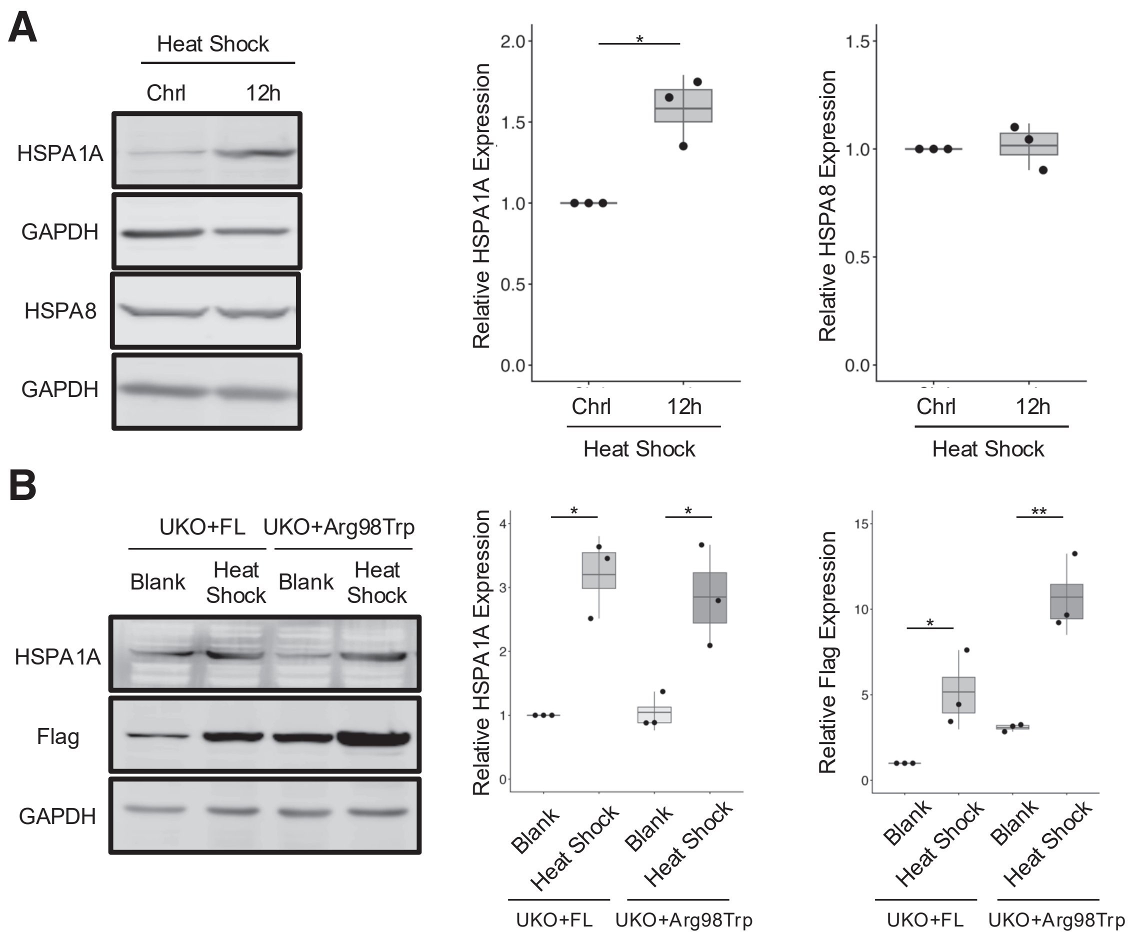

Figure 11. Effect of heat shock on expression of HSPA1A and HSPA8 proteins. (*A* and *B*) Representative WB showing the expression of HSPA1A, HSPA8, and/or the FLAG-tagged mutant in HepG2-WT (*A*), HepG2-UKO+FL (*B*), and HepG2-UKO+R98W (*B*) cells upon treatment with heat shock. *Right-side panels* show quantification of the blots depicted on the *left side* of the figure.

of patients carrying the c.292C>T (p.(Arg98Trp)) variant in *UNC45A*. Such an approach may offer an alternative to existing treatments. Treatment with inhibitors of the apical sodium-dependent bile acid transporter (ASBT; also known as the ileal bile acid transporter) have been proven successful in the treatment of various progressive familial intrahepatic cholestases,[24–28] as well as cases of O2HE syndrome,[29] including the patient mentioned in this study.[14] ASBT inhibitors reduce bile acid resorption in the small intestine, thereby lowering the bile acid load on the liver, and are expected to be effective in patients with residual bile acid secretion capacity.[26,30] However, not all patients may benefit from ASBT inhibitors,[26,30–32] and a continued search for alternative treatment options is warranted. Although treatment with APAP rescued the hepatocellular defect in the cell model, the dose used (5 mM; ∼756 μg/mL) far exceeds the therapeutic (10–20 μg/mL) as well as toxic dose (>150 μg/mL) in plasma. Furthermore, HepG2 cells unlikely accurately reflect in vivo APAP responses.[33] Because elevated HSPA1A expression in the liver in response to medication reflects liver stress/toxicity inflicted by that medication,[34,35] a therapeutic approach that directly delivers HSPA1A proteins to midacinar hepatocytes may be more appropriate.

An Emerging MYO5B-Centered View of Multiple Cases of Familial Intrahepatic Cholestasis

Our findings, along with recent studies,[5,6,8,17,36] emphasize that the presence in hepatocytes of a dysfunctional MYO5B is a central factor in defective BSEP trafficking and therewith associated cholestasis. The loss of MYO5B as such is not sufficient to cause cholestasis, presumably due to compensatory mechanisms that fail in the presence of dysfunctional MYO5B. MYO5B is a large, dynamic motor protein that requires continuous support and maintenance to function properly.[37,38] Although much of this regulatory process remains poorly understood, a myosin-targeted protein quality control system is beginning to emerge.[39] Our study shows that motor defects caused by impaired MYO5B quality control— due to dysfunctional UNC45A—disrupt BSEP trafficking in a manner similar to missense variants in the *MYO5B* gene itself.[8] In addition to the chaperones UNC45A and HSP70 (this study), recent work has identified cholestasis-

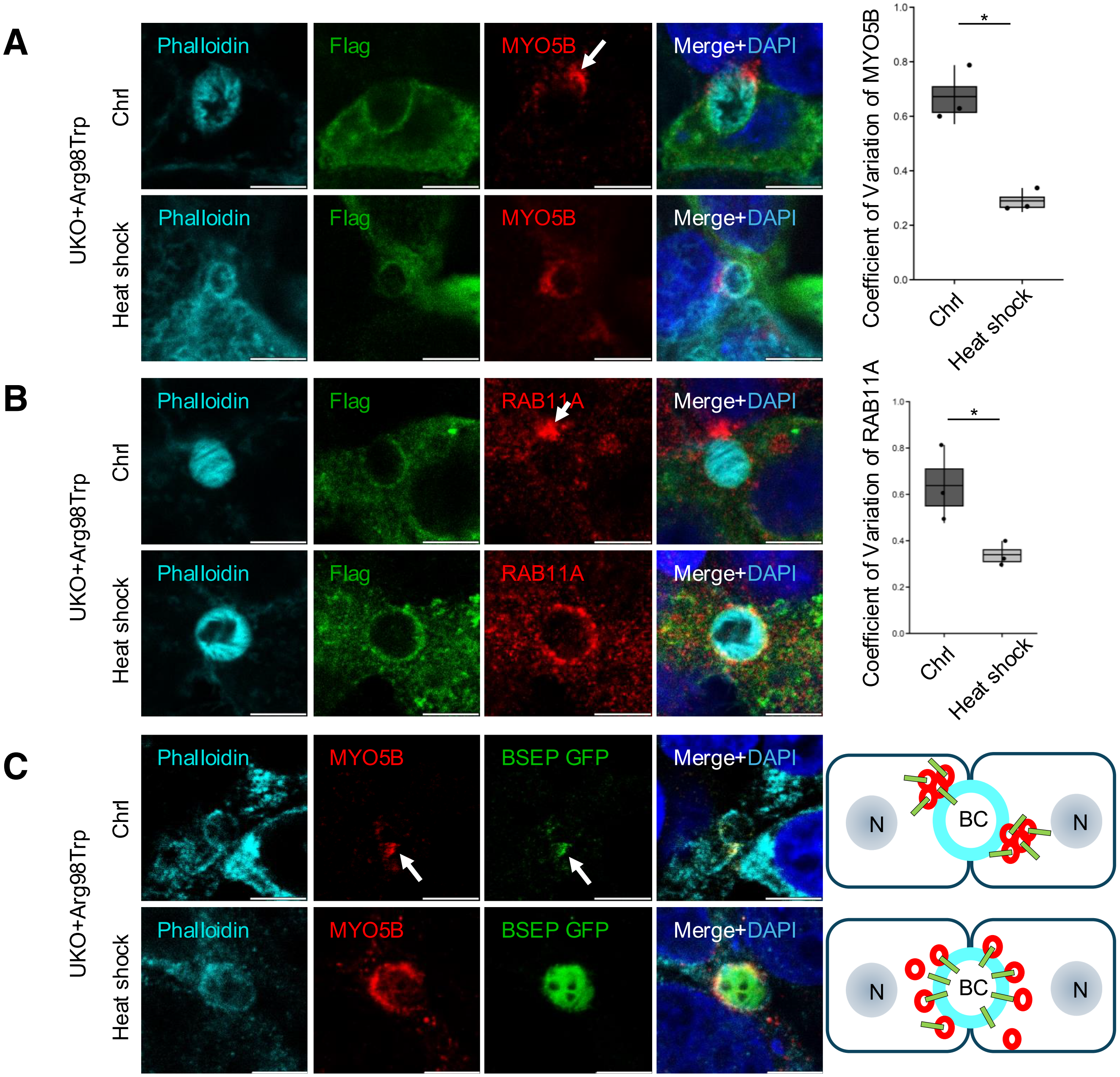

Figure 12. Effect of heat shock on MYO5B, RAB11A, and BSEP distribution in HepG2-UKO+R98W cells. (*A*–*C*) Fluorescence microscopy images showing the change in distribution of MYO5B (*A*), RAB11A (*B*), and BSEP-EGFP (*C*) upon treatment with heat shock. Phalloidin and DAPI are used to stain the BC-enriched F-actin cytoskeleton and nuclei, respectively. Quantification of the coefficients of variation of the IF images shown in (*A*–*C*) are shown on the *right side*. Scale bar, 5 μm.

associated variants of the ubiquitin-specific protease USP53 as regulators of MYO5B. These variants affect the ubiquitination status, dynamics, and function of MYO5B, ultimately impairing the trafficking of BSEP-carrying RAB11A-positive endosomes in hepatocytes.[8] Future research is needed to clarify how different components of the protein quality-control machinery—working in concert—maintain healthy MYO5B function and how their disruption contributes to impaired BSEP trafficking and cholestasis

We demonstrated that the UCS domain of UNC45A as such was sufficient to maintain expression of MYO5B, but that the TPR domain–mediated interaction of UNC45A with active HSP70 was required for a correct functioning of MYO5B on BSEP-carrying RAB11A-positive endosomes. This indicates that multiple domains in UNC45A jointly contribute to MYO5B protein homeostasis and BSEP trafficking. We retrieved 19 reported O2HE-associated *UNC45A* variants from the MEDLINE database using the PubMed search engine and reviewed their predicted structural

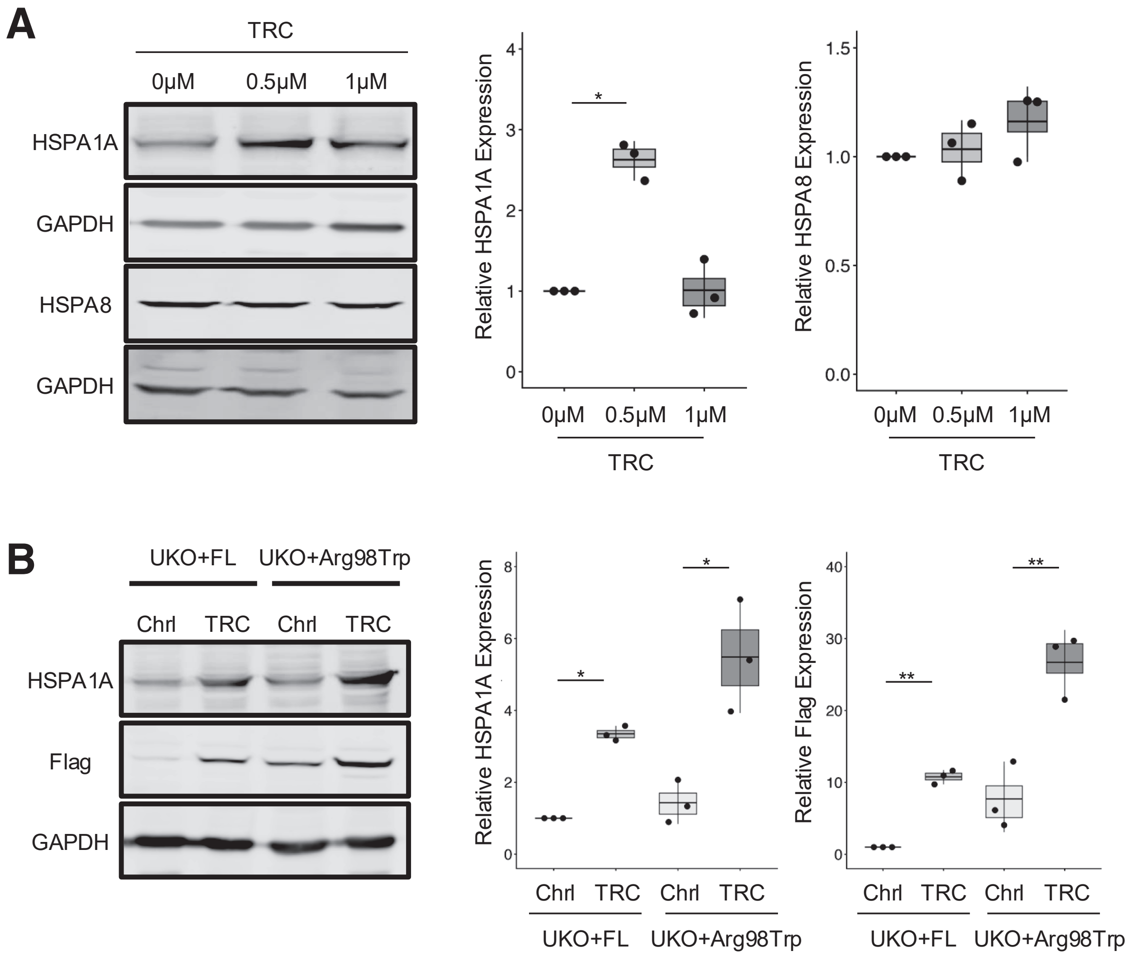

Figure 13. Effect of TRC051384 on expression of HSPA1A and HSPA8 proteins. (*A* and *B*) Representative WB showing the expression of HSPA1A, HSPA8, and/or the FLAG-tagged mutant in HepG2-WT (*A*), HepG2-UKO+FL (*B*), and HepG2-UKO+R98W (*B*) cells upon treatment with TRC051384. *Right side panels* show quantification of the blots depicted on the *left side* of the figure.

changes and interactions and their experimentally demonstrated functional consequences (Supplementary Table 1). Twelve of the 19 variants were missense variants, 5 were nonsense variants, 1 was a frameshift variant, and 1 was an in-frame deletion variant. Only 1 variant, the p.(Arg98Trp) variant studied in this paper and identified in 3 patients with O2HE syndrome,[1,2,14] is located in the HSP70-binding TPR domain. Other variants are located in the central (6 variants), neck (2 variants), or UCS domain (10 variants) that are not directly involved in binding HSP70 and are more likely to affect the stability of the UNC45A protein (eg, p.Val423Asp[9]) or its oligomerization status (eg, p.Leu237Pro[40]), although indirect effects on functional domain positioning through altered interdomain dynamics (eg, as predicted for p.(Thr230Arg)[10]) cannot be excluded. No variants in *UNC45A* identified in patients map directly onto residues previously reported to be directly involved in interaction with myosin proteins, although some are in very close proximity of these (eg, p.Leu757-Pro,[29] which is next to Thr758 and Asn759) (Supplementary Table 1). It will be of interest to discover whether the HSP70-mediated mechanism described here for the c.292C>T (p.(Arg98Trp)) variant also applies to other O2HE-associated *UNC45A* variants.

Materials and Methods

Antibodies

The following primary antibodies were used for IF microscopy and WB analyses. MYO5B was detected using a rabbit polyclonal antibody (Novus Biologicals; NBP1-87746) at a dilution of 1:200 for IF and 1:1000 for WB. RAB11A was detected with a rabbit monoclonal antibody (BD Biosciences; 610,656) used at 1:200 for IF and 1:1000 for WB. UNC45A was detected using a mouse monoclonal antibody (Enzo Life Sciences; ADI-SRA-1800) at a dilution of 1:100 for IF and 1:1000 for WB analyses.

FLAG-tagged proteins were visualized using a mouse monoclonal anti-FLAG antibody (Sigma-Aldrich; F3165), applied at 1:200 for IF and 1:1000 for WB. BSEP was detected using a mouse monoclonal antibody (Santa Cruz Biotechnology; sc-74500) at a dilution of 1:100 for IF staining.

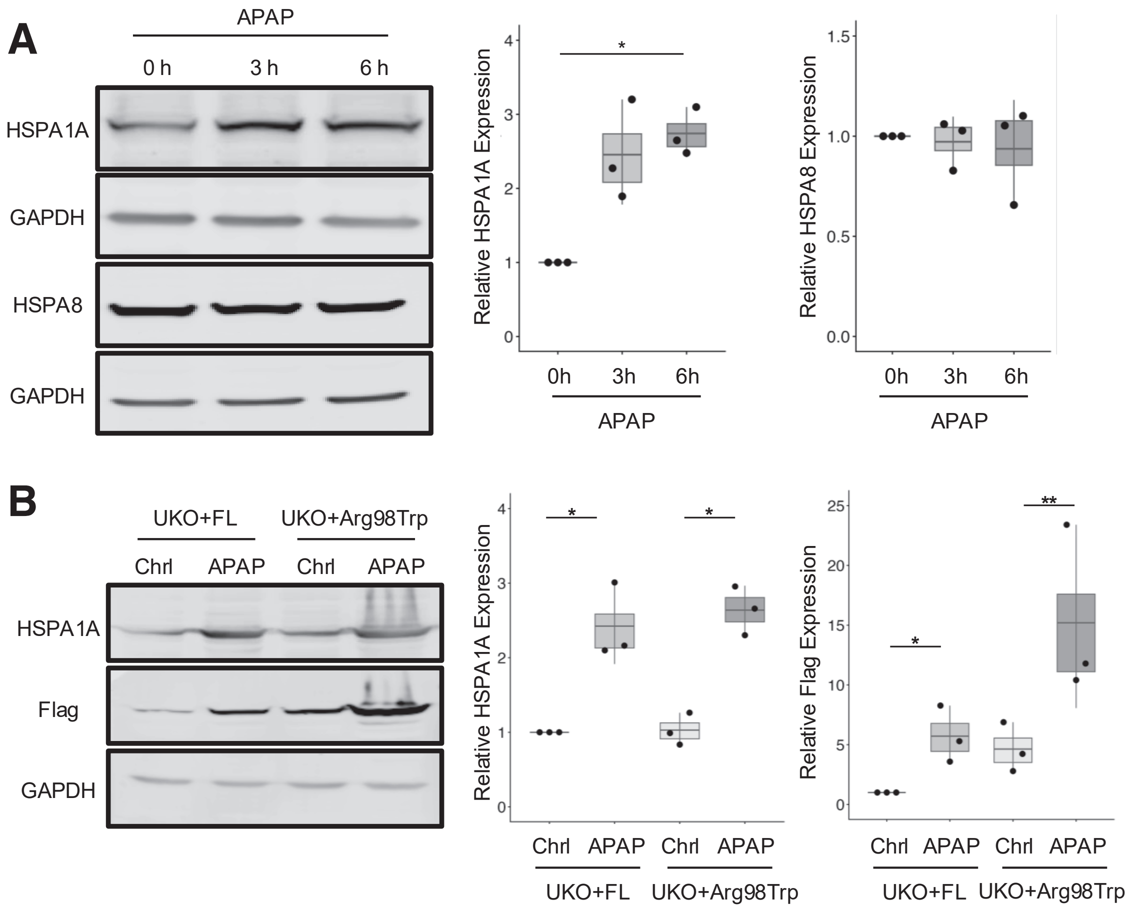

Figure 14. Effect of APAP on expression of HSPA1A and HSPA8 proteins. (*A* and *B*) Representative WB showing the expression of HSPA1A and HSPA8 in HepG2-WT (*A*), HepG2-UKO+FL (*B*), and HepG2-UKO+R98W (*B*) cells upon treatment with APAP. *Right-side panels* show quantification of the blots depicted on the *left side* of the figure.

For loading controls in WB analyses, β-actin was detected using a mouse monoclonal antibody (Sigma-Aldrich; A5441) and glyceraldehyde-3-phosphate dehydrogenase using a mouse monoclonal antibody (Thermo Fisher Scientific; AM4300), both at a dilution of 1:10,000. Heat shock 70 kDa protein 1 (HSPA1A) was detected with a rabbit polyclonal antibody (Enzo Life Sciences; ADI-SPA-812) and HSPA8 with a rat monoclonal antibody (Enzo Life Sciences; ADI-SPA-815); both antibodies were used at a dilution of 1:1000 for WB.

For visualization of filamentous actin in IF experiments, Alexa Fluor 633-conjugated phalloidin (Invitrogen; A22284) was used at a dilution of 1:1000.

Cell Culture

HepG2 Cells (ATCC-HB8065) were maintained in high-glucose Dulbecco's Modified Eagle Medium (Thermo Scientific Fisher; 11,965,084) with 10% heat-inactivated fetal calf serum (Invitrogen, Altham) and 1% penicillin–streptomycin (Thermo Scientific Fisher; 15,140,122) at 37 °C in a humidified 5% CO_2 incubator and used between passages 3 and 15. HepG2 cells in which *UNC45A* was inactivated by CRISPR-CRISPR–associated protein 9 technology (referred to as HepG2-UKO cells) were generated previously.[9] Cells were passaged at 80% to 90% confluence using 0.25% trypsin (Invitrogen; 27,250-018).

For plasmid transfection, cells were seeded at 60% to 70% confluence in 24-well plates. Plasmids were transfected into cells using Fugene (Promega; E3211) according to the manufacturer's instructions. Cells were harvested 48 hours post-transfection.

For pharmacologic inhibition of HSP70 activity, cells were treated with VER-155008 (Sigma-Aldrich; SML0271) (40 μM for 4, 6, or 8 hours). Acetaminophen (APAP; Sigma-Aldrich, A7085) was applied at 5 mM for 3 or 6 hours. TRC051384 (Selleckchem; S8305) was used at 0.5 μM and 1 μM for 6 hours. For heat shock treatment, cells were transferred to a prewarmed incubator at 43 °C for 1 hour and returned to 37 °C for a 12-hour recovery period. Vehicle control cells received equivalent volumes of dimethyl sulfoxide or phosphate-buffered saline (PBS).

UNC45A–FLAG Rescue and Site-Directed Mutagenesis

Full-length human *UNC45A* complementary DNA was amplified from pCMV6-Entry-*UNC45A* (Origene; RC206953)

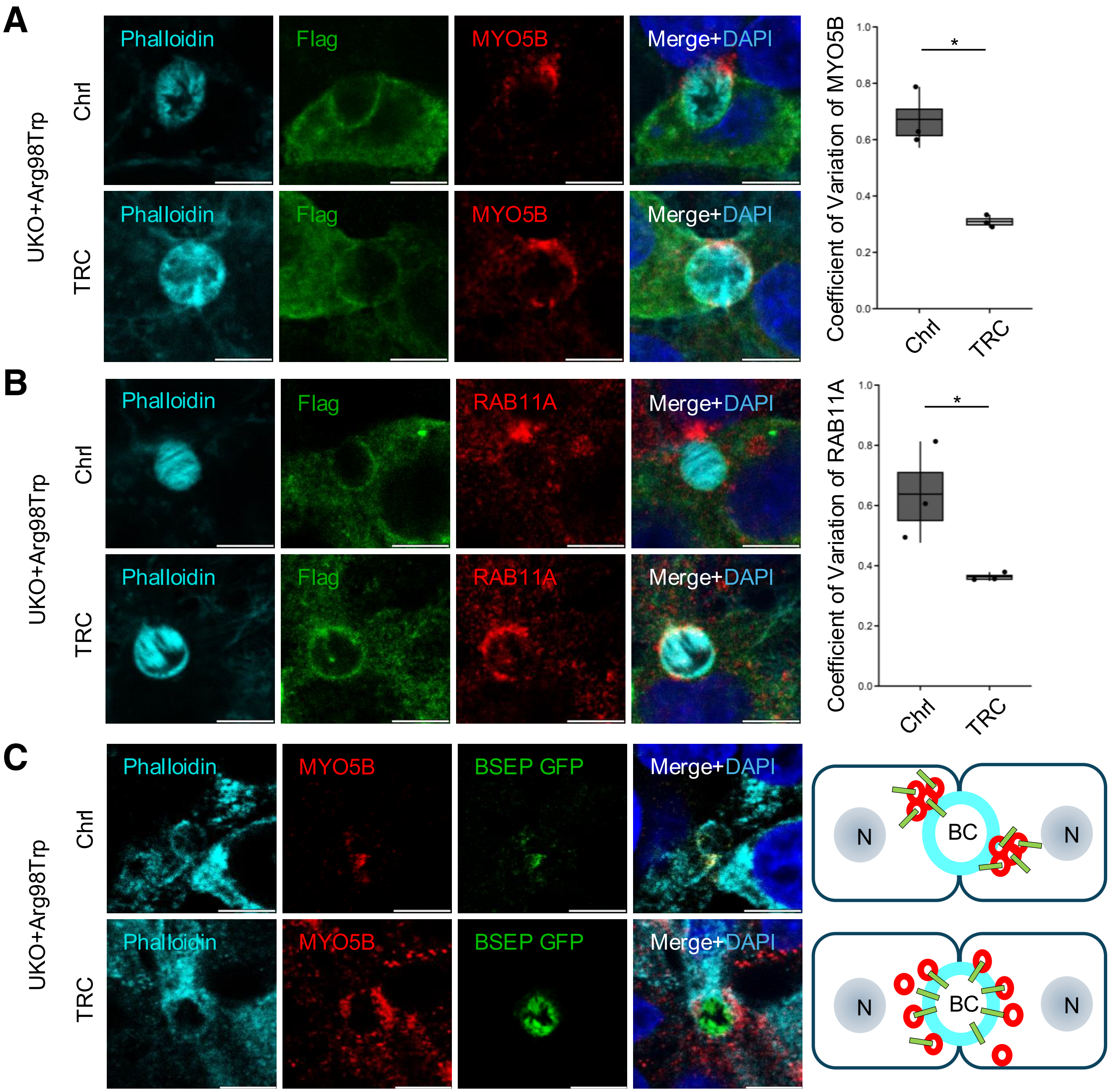

Figure 15. Effect of TRC051384 on MYO5B, RAB11A, and BSEP distribution in HepG2-UKO+R98W cells. (*A*–*C*) Fluorescence microscopy images showing the change in distribution of MYO5B (*A*) RAB11A (*B*), and BSEP-EGFP (*C*) upon treatment with TRC051384. Phalloidin and DAPI are used to stain the BC-enriched F-actin cytoskeleton and nuclei, respectively. Quantification of the CVs of the IF images shown in (*A*–*C*) are shown on the *right side*. Scale bar, 5 μm.

and subcloned into pENTR1a. The *MYC* tag was deleted using the Q5 Site-Directed Mutagenesis Kit (New England Biolabs; E0554S) with primers designed using the NEBaseChanger tool. Patient-derived point mutations were introduced on the *MYC*-deleted *UNC45A* construct by site-directed mutagenesis. WT and mutant *UNC45A-FLAG* constructs were cloned into lentiviral expression vectors. Lentiviral particles were produced in HEK293T (ATCC-CRL-1573) cells by cotransfecting 1200 ng lentiCRISPR v2, 1000 ng pCMVdR8.1, and 400 ng pVSV-G using FuGENE Transfection Reagent. Viral supernatants were collected 48 hours post-transfection, filtered through a 0.45-μm polyvinylidene difluoride (PVDF) filter, and used to transduce HepG2 cells in the presence of 8 μg/mL polybrene. Following overnight incubation, transduced cells were selected with appropriate antibiotics for at least 7 days. Single clones were expanded, and knockout was verified by Sanger sequencing and immunoblotting. Lentiviral particles were used to transduce UKO HepG2 cells. Stable cell lines were established by selection with appropriate antibiotics.

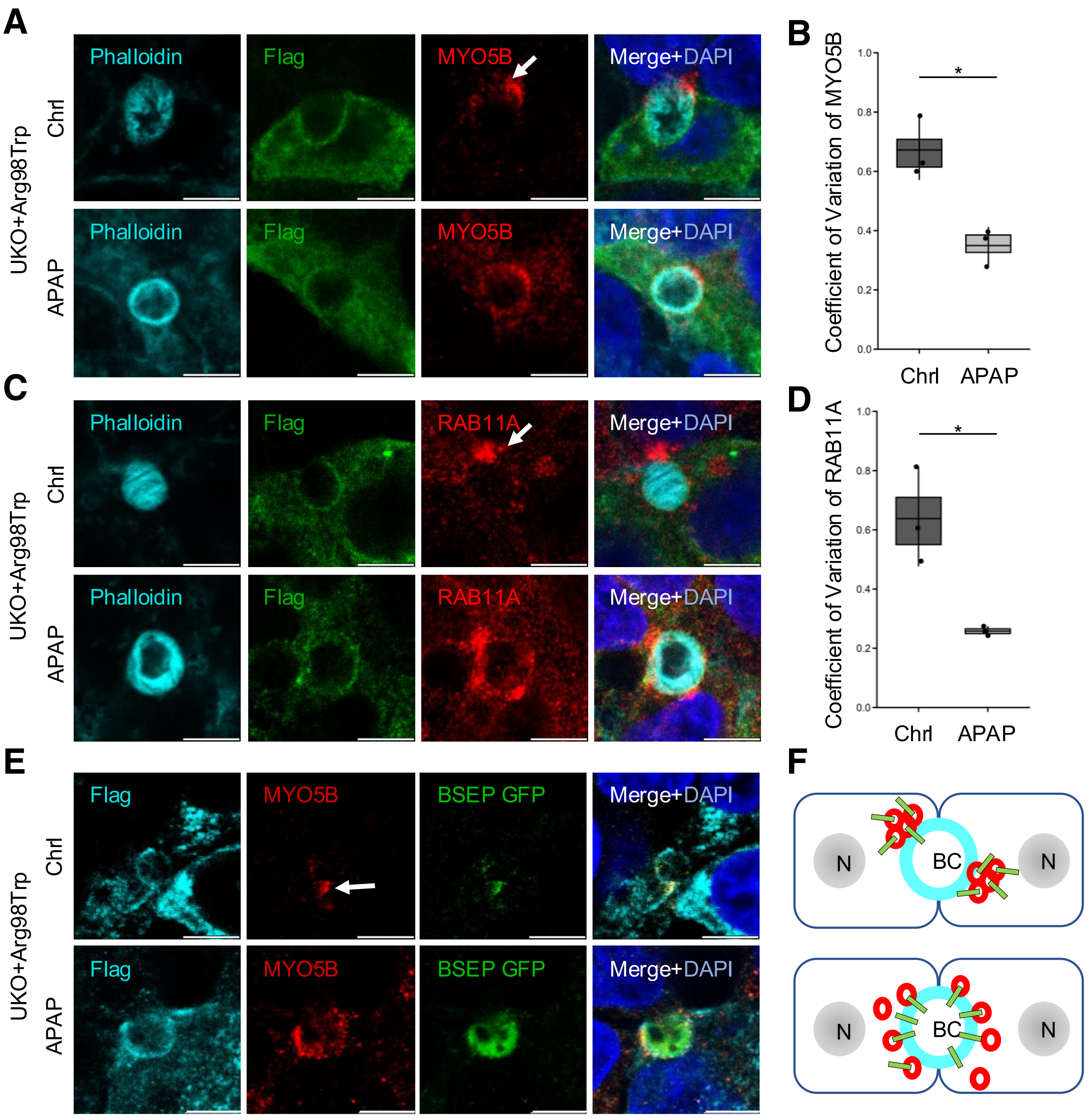

Figure 16. Effect of APAP on MYO5B, RAB11A, and BSEP distribution in HepG2-UKO+R98W cells. (*A, C,* and *E*) fluorescence microscopy images showing the change in distribution of MYO5B (*A*), RAB11A (*C*), and BSEP-EGFP (*E*) upon treatment with APAP. Phalloidin and DAPI are used to stain the BC-enriched F-actin cytoskeleton and nuclei, respectively. (*B* and *D*) Quantification of the CVs of the IF images shown in (*A, C,* and *E*). (*F*) Shows cartoon of distribution of Rab11a+ endodome (*red*) with BSEP (*green*) with respect to the bile canalicular membrane (*turqoise*). Scale bar, 5 μm.

For the ΔTPR construct, the forward primer was *AAAGTTT TCCAGGAGGCC*, and the reverse primer was *TGAGCTGGCAGT-CATGGA*. For UCS, the forward primer was *GCTGGAGGGACT-GACTTC*, and the reverse primer was *GGCAGTCATGGATCCAGT*.

For the K93E mutation, the forward primer was *TGGGGATGTCGAAGCACTCTACCG*, and the reverse primer was *CCATCCTTTTCAATGGCTTTGGATGC*. For the R98W mutation, the forward primer was *CACTCTACTGGCGGAGCCAA*, and the reverse primer was *CTTTGACATCCCCACCATCCT*.

Sodium Dodecyl Sulfate-Polyacrylamide Gel Electrophoresis and Western Blotting

Cell lysates were prepared in ice-cold radio-immunoprecipitation assay buffer (50 mM Tris-HCl pH 7.5,

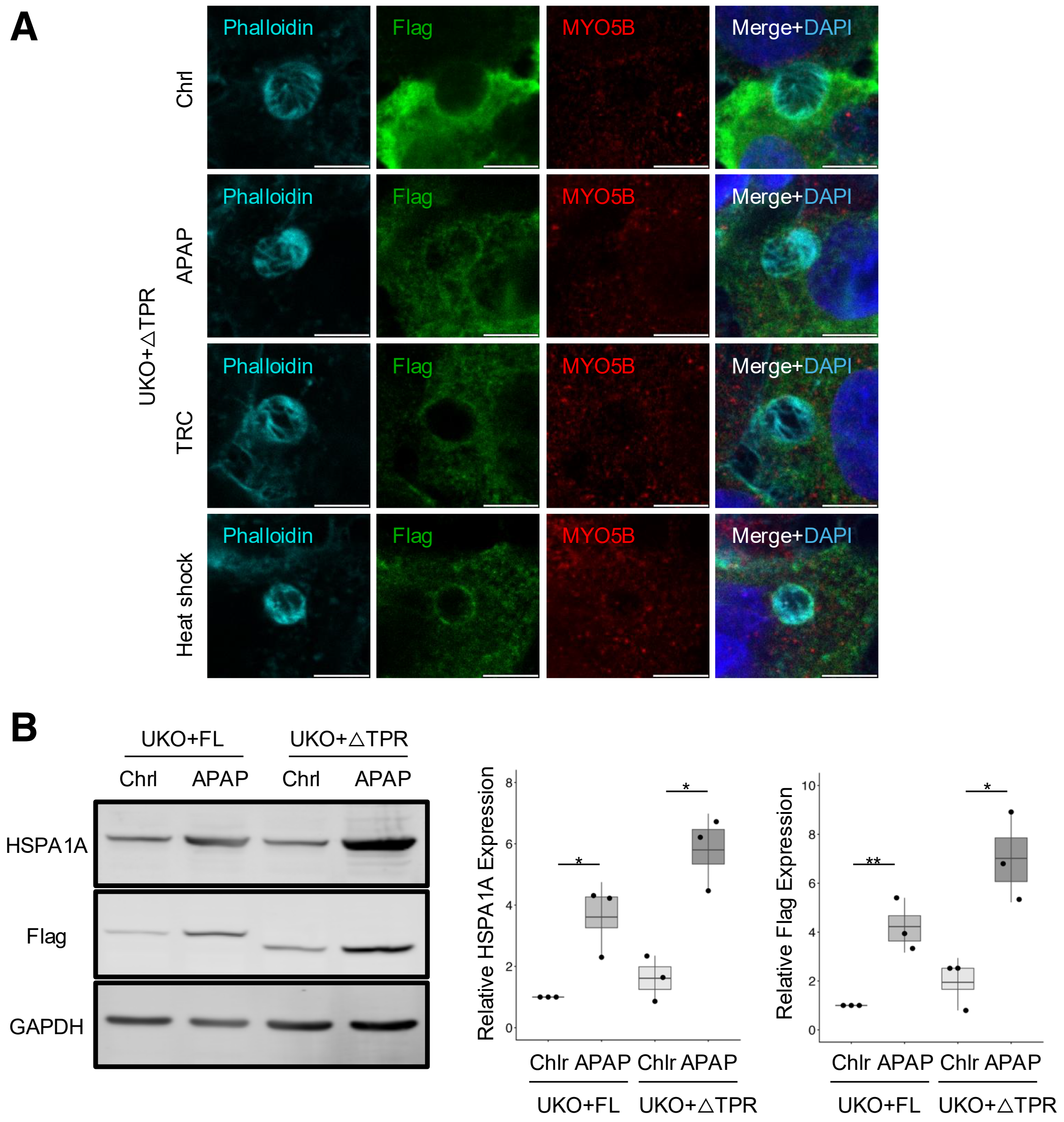

Figure 17. Effect of APAP or heat shock on MYO5B, RAB11A, and BSEP distribution in HepG2-UKO + ΔTPR cells. (*A*) Fluorescence microscopy images showing the change in distribution of Myo5b upon treatment with APAP or heat-shock. Phalloidin and DAPI are used to stain the BC-enriched F-actin cytoskeleton and nuclei, respectively. Scale bar, 5 μm. (*B*) Representative WB showing the expression of HSPA1A and FLAG-tagged mutant in HepG2-UKO+FL and HepG2-UKO+ΔTPR cells upon treatment with APAP. *Right-side panels* show quantification of the blots depicted on the *left side* of the figure.

150 mM NaCl, 1% Triton X-100, 0.5% sodium deoxycholate, 0.1% sodium dodecyl sulfate [SDS]) supplemented with protease inhibitors cocktail (Roche; 11,836,153,001). Protein concentration was determined by bicinchoninic acid. Equal amounts of protein were denatured in 2× Laemmli buffer (Bio-Rad; #1610737EDU) supplemented with 5% (vol/vol) 2-mercaptoethanol (Sigma-Aldrich; M6250) at 95 °C for 5 minutes, and resolved by SDS–polyacrylamide gel electrophoresis (PAGE) using a 4% stacking gel and an 7.5% to 12% resolving gel. Proteins were transferred to PVDF membranes (Millipore; IPVH85R) using wet transfer (120 V; 75 minutes; 4 °C). Membranes were blocked in blocking buffer (LICORbio; 927-70001) for 1 hour at room temperature, incubated with primary antibodies overnight at 4 °C, washed (TBST, 3 × 10 min), and probed with IRDye 680/800-conjugated secondary antibodies at room temperature for 1 hour. Fluorescent signals were visualized using a LI-COR imaging system. Bands were normalized to β-actin/glyceraldehyde-3-phosphate dehydrogenase and quantified in LI-COR Image Studio.

Coimmunoprecipitation

Cells were harvested after 72 hours of culturing and lysed on ice in lysis buffer (10 mM Tris-HCl pH 7.5, 150 mM NaCl, 0.5 mM EDTA, 0.5% Nonidet P40 substitute) supplemented with protease inhibitors. Lysates were diluted 1:1 with the kit Dilution buffer. Diluted lysates were incubated with DYKDDDDK Fab-Trap agarose beads (Chromotek; ffak) at 4 °C with end-over-end rotation for 1 hour. Beads were washed 3 times with wash buffer, and bound proteins were eluted in Laemmli buffer with 5% 2-mercaptoethanol. Cell lysis and immunoprecipitates were analyzed by SDS-PAGE and PVDF immunoblotting with the indicated antibodies

Quantification of Coimmunoprecipitation

Background-subtracted band intensities were quantified (LI-COR Image Studio). For each immunoprecipitation (IP) lane, a co-IP/FLAG ratio was computed as the signal of the coprecipitated target divided by the signal of FLAG-UNC45A in the same lane: $\text{co-IP/FLAG ratio} = \frac{S_{\text{IP}}^{\text{target (HSPA1A/HSPA8/MYO5B)}}}{S_{\text{IP}}^{\text{FLAG-UNC45A}}}$. Ratios on each membrane were scaled to the mean of the full-length (full-length UNC45A) control on that membrane, defined as 1 (100%): $\text{Interaction (\% of full-length)} = 100 \times \frac{(\text{co-IP/FLAG ratio})_{\text{sample}}}{(\text{co-IP/FLAG ratio})_{\text{FL (same membrane)}}}$.[41]

Immunofluorescence Microscopy

Liver specimens from a patient with *UNC45A*-associated O2HE syndrome and healthy control were fixed in 4% paraformaldehyde, embedded in paraffin, and sectioned at 4 μm. Heat-induced epitope retrieval was performed in citrate buffer (10 mM, pH 6.0) for 20 minutes at ∼95 °C to 98 °C (microwave), followed by 10 minutes cooling, and blocked with 5% bovine serum albumin (Sigma; A7906) for 1 hour. Sections were then incubated overnight at 4 °C with a primary antibody. After PBS washes, sections were incubated with Alexa Fluor–conjugated secondary antibody for 1 hour at 37 °C in the dark. Slides were mounted with an antifade mounting medium.

For IF experiments, HepG2 cells were plated on poly-L-lysine (Sigma; P-2636)–coated coverslips and fixed with 4% paraformaldehyde at room temperature for 30 minutes, followed by permeabilization with 0.1% Triton X-100 in PBS for 10 minutes. After washing, nonspecific binding sites were blocked with 5% bovine serum albumin in PBS at 37 °C for 1 hour. Coverslips were then incubated overnight at 4 °C with primary antibody. After washes, cells were incubated with Alexa Fluor–conjugated secondary antibody at 37 °C in the dark for 1 hour, and coverslips were mounted onto glass slides using an antifade mounting medium. Fluorescence images were acquired using a Leica SP8X under identical exposure settings across all groups.

Quantification of Angular Heterogeneity of Pericanalicular MYO5B/RAB11A Intensity

IF images were acquired under identical exposure conditions to ensure comparability between samples. Bile canaliculi (BCs) were identified and segmented from the canalicular marker (fluorescently labeled phalloidin). A ring-shaped peri-canalicular region of interest (ROI) was generated to capture the cortical MYO5B/RAB11A signal, while excluding the luminal area and distant cytoplasmic background. Each peri-canalicular ring ROI was divided into 10 equal angular sectors (36° each). Using *ImageJ*, the mean fluorescence intensity of each sector k ($k = 1, 2, \ldots, 10$) (I_k) and the mean intensity across the entire ring ROI ($\bar{I}$) were obtained. From these data, the standard deviation $= \sqrt{\frac{1}{n-1} \sum_{k=1}^{n} (I_k - \bar{I})^2}$ and the angular coefficient of variation $(CV(\%)) = \frac{SD}{\bar{I}} \times 100$ was calculated. The parameter of the CV reflects the degree of circumferential heterogeneity of MYO5B/RAB11A distribution around the BC. A low CV indicates a uniform signal, whereas a high CV reflects irregular distribution. For each BC, one CV value was obtained. Multiple BCs were analyzed per field, and the field-average CV was used as 1 biological replicate for statistical comparisons. For each experimental group, CV values from multiple fields were pooled for statistical analysis. Data are presented as mean ± standard error of the mean.

Statistical Analysis

GraphPad Prism 10 and R were used for data analysis. Data are presented as mean ± standard error of the mean. Student's t test was used for comparisons between 2 groups. A P value $< .05$ was considered statistically significant. Significance levels are indicated as *$P < .05$, **$P < .01$, *** $P < .001$, and ****$P < .0001$.

Declaration of Generative AI and AI-Assisted Technologies in the Writing Process

During the preparation of this work the authors used Microsoft Copilot (M365 Copilot, GPT-5–based model; Microsoft Corporation) in order to assist with grammar correction, wording clarification, and stylistic polishing of selected sections of this manuscript during the time of manuscript preparation. The AI tool was not used to generate scientific content, conceptual interpretations, or conclusions. After using this tool/service, the authors reviewed and edited the content as needed and take full responsibility for the content of the publication.

Supplementary Material

Note: To access the supplementary material accompanying this article, visit the full text version at https://doi.org/10.1016/j.jcmgh.2026.101805.

References

1. Esteve C, Francescatto L, Tan PL, et al. Loss-of-function mutations in UNC45A cause a syndrome associating cholestasis, diarrhea, impaired hearing, and bone fragility. Am J Hum Genet 2018;102:364–374.

2. Wang R, Wang Y, Yu R, et al. Case report: osteo-oto-hepato-enteric syndrome caused by UNC45A deficiency. Front Genet 2022;13:1079481.
3. Almaas R, Atneosen-Åsegg M, Ytre-Arne ME, et al. Aagenaes syndrome/lymphedema cholestasis syndrome 1 is caused by a founder variant in the 5'-untranslated region of UNC45A. J Hepatol 2023;79:945–954.
4. Wakabayashi Y, Kipp H, Arias IM. Transporters on demand: intracellular reservoirs and cycling of bile canalicular ABC transporters. J Biol Chem 2006;281:27669–27673.
5. Qiu YL, Gong JY, Feng JY, et al. Defects in myosin VB are associated with a spectrum of previously undiagnosed low γ-glutamyltransferase cholestasis. Hepatology 2017;65:1655–1669.
6. Gonzales E, Taylor SA, Davit-Spraul A, et al. MYO5B mutations cause cholestasis with normal serum gamma-glutamyl transferase activity in children without microvillous inclusion disease. Hepatology 2017;65:164–173.
7. Stieger B, Meier Y, Meier PJ. The bile salt export pump. Pflugers Arch 2007;453:611–620.
8. Overeem AW, Li Q, Qiu YL, et al. A molecular mechanism underlying genotype-specific intrahepatic cholestasis resulting from MYO5B mutations. Hepatology 2020;72:213–229.
9. Li Q, Zhou Z, Sun Y, et al. A functional relationship between UNC45A and MYO5B connects two rare diseases with shared enteropathy. Cell Mol Gastroenterol Hepatol 2022;14:295–310.
10. Duclaux-Loras R, Lebreton C, Berthelet J, et al. UNC45A deficiency causes microvillus inclusion disease-like phenotype by impairing myosin VB-dependent apical trafficking. J Clin Invest 2022;132:e154997.
11. Lee CF, Melkani GC, Bernstein SI. The UNC-45 myosin chaperone: from worms to flies to vertebrates. Int Rev Cell Mol Biol 2014;313:103–144.
12. Piper PW, Scott JE, Millson SH. UCS chaperone folding of the myosin head: a function that evolved before animals and fungi diverged from a common ancestor more than a billion years ago. Biomolecules 2022;12:1028.
13. Bolder U, Jeschke MG, Landmann L, et al. Heat stress enhances recovery of hepatocyte bile acid and organic anion transporters in endotoxemic rats by multiple mechanisms. Cell Stress Chaperones 2006;11:89–100.
14. Imhann F, Zhou Z, Malcolm CFS; 'Support and Supervision Team'. Odevixibat treatment for recurrent cholestasis in a patient with biallelic UNC45A variants causing osteo-oto-hepato-enteric syndrome. JHEP Rep Innov Hepatol 2025;8:101710.
15. Murphy WA, Diehl AM, Loop MS, et al. Alterations in zonal distribution and plasma membrane localization of hepatocyte bile acid transporters in patients with NAFLD. Hepatol Commun 2024;8:e0377.
16. Wakabayashi Y, Dutt P, Lippincott-Schwartz J, Arias IM. Rab11a and myosin Vb are required for bile canalicular formation in WIF-B9 cells. Proc Natl Acad Sci U S A 2005;102:15087–15092.
17. She HY, Qiu YL, Feng JY, et al. A liver-specific mouse model for MYO5B-associated cholestasis reveals a toxic gain-of-function as underlying disease mechanism. Biochem Biophys Res Commun 2025;758:151669.
18. Gazda L, Pokrzywa W, Hellerschmied D, et al. The myosin chaperone UNC-45 is organized in tandem modules to support myofilament formation in C. elegans. Cell 2013;152:183–195.
19. Sun M, Pylypenko O, Zhou Z, et al. Uncovering the relationship between genes and phenotypes beyond the gut in microvillus inclusion disease. Cell Mol Gastroenterol Hepatol 2024;17:983–1005.
20. Hellerschmied D, Lehner A, Franicevic N, et al. Molecular features of the UNC-45 chaperone critical for binding and folding muscle myosin. Nat Commun 2019;10:4781.
21. Williamson DS, Borgognoni J, Clay A, et al. Novel adenosine-derived inhibitors of 70 kDa heat shock protein, discovered through structure-based design. J Med Chem 2009;52:1510–1513.
22. Mohanan A, Deshpande S, Jamadarkhana PG, et al. Delayed intervention in experimental stroke with TRC051384–a small molecule HSP70 inducer. Neuropharmacology 2011;60:991–999.
23. Salminen WF, Voellmy R, Roberts SM. Effect of N-acetylcysteine on heat shock protein induction by acetaminophen in mouse liver. J Pharmacol Exp Ther 1998;286:519–524.
24. Roquelaure B, Sciveres M, Grammatikopoulos T, et al. Odevixibat therapy in progressive familial intrahepatic cholestasis with MYO5B variants: a retrospective case series. Orphanet J Rare Dis 2025;20:227.
25. Thompson RJ, Artan R, Baumann U, et al. Interim results from an ongoing, open-label, single-arm trial of odevixibat in progressive familial intrahepatic cholestasis. JHEP Rep Innov Hepatol 2023;5:100782.
26. Di Giorgio A, Sciveres M, Fuoti M, et al. Real-world experience with odevixibat in children with progressive familial intrahepatic cholestasis. JHEP Rep 2024;7:101309.
27. Ala AS, Sayar T, Islek A, et al. Managing PFIC 7 with odevixibat: alleviation of pruritus and biochemical response. JHEP Rep Innov Hepatol 2026;8:101593.
28. Bedoyan SM, Lovell OT, Horslen SP, Squires JE. Odevixibat: a promising new treatment for progressive familial intrahepatic cholestasis. Expert Opin Pharmacother 2022;23:1771–1779.
29. Fiedler A, Brittan K, Manatsathit W. Microvillous inclusion disease: an exceedingly rare condition with a new treatment. ACG Case Rep J 2024;11:e01537.
30. Nomden M, Kuipers F, Lexmond WS, et al. Pretreatment serum bile acid composition and predictability of subsequent response to odevixibat in patients with bile salt export pump (BSEP) deficiency. Hepatology 2026;83:708–720.
31. Herta T, Dröge C, Herber A, et al. Odevixibat treatment in an adult patient with advanced icteric progressive cholestatic liver disease. JHEP Rep 2023;6:100978.
32. Sutton H, Sokol RJ, Kamath BM. IBAT inhibitors in pediatric cholestatic liver diseases: transformation on the horizon? Hepatology 2025;82:985–995.
33. Lőrincz T, Deák V, Makk-Merczel K, et al. The performance of HepG2 and HepaRG systems through the glass of acetaminophen-induced toxicity. Life 2021;11:856.
34. Tsuji D, Akagi R. Species-specific differences in acetaminophen hepatotoxicity depend on HSP70 expression level. J Biochem (Tokyo) 2025;177:133–139.

35. Levada K, Guldiken N, Zhang X, et al. Hsp72 protects against liver injury via attenuation of hepatocellular death, oxidative stress, and JNK signaling. J Hepatol 2018;68:996–1005.
36. Ding J, She HY, Cheng Y, et al. A novel mechanism involving USP53-regulated BSEP trafficking underlies low-GGT intrahepatic cholestasis. Hepatology 2026;83:1383–1397.
37. Trybus KM. Myosin V from head to tail. Cell Mol Life Sci 2008;65:1378–1389.
38. Zhang N, Yao LL, Li XD. Regulation of class V myosin. Cell Mol Life Sci 2018;75:261–273.
39. Vogel A, Arnese R, Gudino Carrillo RM, et al. UNC-45 assisted myosin folding depends on a conserved FX3HY motif implicated in Freeman Sheldon Syndrome. Nat Commun 2024;15:6272.
40. Waich S, Kreidl K, Vodopiutz J, et al. Altered chaperone-nonmuscle myosin II interactions drive pathogenicity of the UNC45A c.710T>C variant in osteo-oto-hepato-enteric syndrome. JCI Insight 2025;10:e185508.
41. Burckhardt CJ, Minna JD, Danuser G. Co-immunoprecipitation and semi-quantitative immunoblotting for the analysis of protein-protein interactions. STAR Protoc 2021;2:100644.

Received February 10, 2026. Accepted May 1, 2026.

Correspondence

Address correspondence to: Sven C.D. van IJzendoorn, PhD, University Medical Centre Groningen, Antonius Deusinglaan 1, Groningen 9713AV, the Netherlands. e-mail: s.c.d.van.ijzendoorn@umcg.nl; or Qinghong Li, PhD, Department of Gastroenterology and Hepatology, Tytgat Institute for Liver and Intestinal Research, Amsterdam Gastroenterology Endocrinology Metabolism (AGEM), Amsterdam UMC, University of Amsterdam, Meibergdreef 9, 1105 AZ, Amsterdam, the Netherlands. e-mail: q.li@amsterdamumc.nl.

Acknowledgments

RAB11A-EGFP and BSEP-EGFP expression constructs used in this study were provided by R.E. Pagano (Mayo Clinic) and J.S. Wang (The Center for Pediatric Liver Diseases, Children's Hospital of Fudan University, National Children's Medical Center, Shanghai, China), respectively. The anti-HSPA8 antibody was a gift from Dr M. Mauthe (University Medical Center Groningen, the Netherlands). We are grateful to Dr N. Grzeschik, Dr R. Gómez Sánchez, Dr K. Sjollema, E. Armenaki, C. Usanase, E. Bakker, and J. de Leeuw for technical assistance. Part of the work was performed in the University Medical Center Groningen Imaging Center.

CRediT Authorship Contributions

Yuan Lin (Writing – review & editing, Methodology, Investigation, Formal analysis, Conceptualization)

Zhe Zhou (Writing – review & editing, Visualization, Methodology, Investigation, Formal analysis, Data curation, Conceptualization)

Henkjan verkade (Writing – review & editing, Supervision, Investigation)

floris immhan (Writing – review & editing, Methodology, Investigation)

Sven van IJzendoorn (Writing – original draft, Supervision, Project administration, Methodology, Investigation, Data curation, Conceptualization)

Qinghong Li (Writing – review & editing, Supervision, Resources, Methodology, Investigation, Conceptualization)

Conflicts of interest

The authors disclose no conflicts.

Funding

Zhe Zhou, Yuan Lin, and Qinghong Li were supported by scholarships from the Chinese Scholarship Counsel (CSC).

Data Availability

Data, analytic methods, and study materials will be made available to other researchers upon request.

Cellular and Molecular Gastroenterology and Hepatology, Volume *20*

Supplemental information

A Molecular Mechanism of Intrahepatic Cholestasis in Osteo-Oto-Hepato-Enteric Syndrome

Zhe Zhou, Yuan Lin, Floris Imhann, Henkjan J. Verkade, Qinghong Li, and Sven C.D. van IJzendoorn

Supplementary table 1. Overview of all O2HE-associated UNC45A variants, predicted effects on amino acid changes, predicted consequences on protein structure and function and experimentally demonstrated consequences. Figure shows only missense and deletion variants.

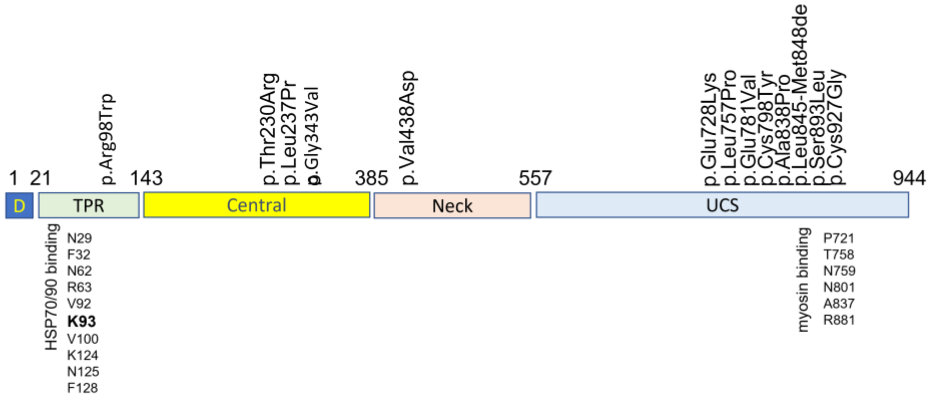

Figure on the left shows missense variants only.

HSP70/90-binding residues are inferred from:

Gazda L, Pokrzywa W, Hellerschmied D, et al. The myosin chaperone UNC-45 is organized in tandem modules to support myofilament formation in C. elegans. Cell. 2013 Jan 17;152(1-2):183-95. doi: 10.1016/j.cell.2012.12.025.

Myosin-binding residues are inferred from:

Vogel A, Arnese R, Gudino Carrillo RM, et al., UNC-45 assisted myosin folding depends on a conserved FX3HY motif implicated in Freeman Sheldon Syndrome. Nat Commun. 2024 Jul 25;15(1):6272. doi: 10.1038/s41467-024-50442-6.

Reference PUBMED ID	Patient ID	Gene variant	Protein prediction	Domain	Predicted consequences on protein structure and function#	Experimentally demonstrated consequences
29429573	P7[1]	c.247C > T	p.Arg83Trp[1]	TPR	See footnote [1]	See footnote [1]
36699472/<br>41861679/<br>29429573[1]	P3/<br>P1/<br>P7[1]	c.292C > T	p.Arg98Trp	TPR	The bulky Trp side chain is not compatible with the neighboring helices positioned within the TPR domain, it might affect the HSP90/70 binding site within the TPR domain	This study:<br>- Reduced expression in patient liver tissue (with additional Gln198∗ variant)<br>- Reduced interaction with HSP70<br>- Defective myosin Vb function.<br>- Defective Rab11a-mediated BSEP trafficking<br>- Aberrant BSEP IHC in patient liver tissue (with additional Gln198∗ variant)

41861679	P1	c.592C>T	Gln198*	Central	Protein truncated at the central domain, protein expression is inhibited	- No protein expression.
35575086	P4	c.689C > G	p.Thr230Arg	Central	Mapped in the helix 1 of the 3rd ARM repeat (3H1) of the central domain, introducing the long side chain of an Arg at the position of the Thr might result in steric hindrance with surrounding residues destabilizing the protein. May alter the positioning of the TRP domain and thus protein conformation.	PMID 35575086:<br>- No effect on protein expression when expressed in HEK cells
36587802/<br>35575086/<br>35575086/<br>35575086	P2/<br>P1/<br>P5/<br>P6	c.710 T > C	p.Leu237Pro	Central	Mapped in the helix 1 of the 3rd ARM repeat (3H1) of the central domain, compatible with the structure; however, may result in loss of a key backbone H-bond and in helix destabilization and a local structural rearrangement. May alter the positioning of the TRP domain and thus protein conformation.	PMID 36587802:<br>- protein expression of the UNC45A-Leu237Pro group decreased by 27.4% when expressed in HEK cells<br><br>PMID 35575086:<br>- No effect on protein expression when expressed in HEK cells<br><br>PMID 40125554:<br>-in T lymphoblastic cells and in fibroblasts isolated from skin biopsies from two patients, protein levels are comparable to those of control cells.<br>- Disrupts chaperone-myosin interaction.<br>- Retained chaperone activity.<br>- Prevented myosin aggregation.<br>- Supported NMII filament formation in patient fibroblasts and U2OS cells.<br>- Formed atypically stable oligomers.

						- Prevented chaperone-myosin complex dissociation.<br>- Inhibited NMII functions.<br>- Impaired intracellular trafficking.<br>- considerably lowered the thermal resistance of UNC45A
35575086	P2	c.721C > T	p.Arg241*	Central	Protein truncated within the central domain is predicted to inhibit expression.	PMID 35575086:<br>- No protein expression when expressed in cultured cells
29429573	P4	c.784C > T	p.Arg262*[2]<br>p.Arg277*	Central	The truncation generates a protein with a short, unstable central domain and lacking both the neck and UCS domains.	- No protein expression
29429573	P7	c.983G > T	p.Gly328Val[2]<br>p.Gly343Val	Central	The residue substitution is compatible with the protein's structural integrity.	ND
29429573	P4	c.1268 T > A	p.Val423Asp[2]<br>p.Val438Asp	Neck	In the neck domain, substitution of the hydrophobic valine with the charged aspartate in the hydrophobic core is likely to destabilize the protein.	PMID 35421597:<br>- Unstable protein.<br>- Retained chaperone activity.<br>(Based on Val423Asp)
35575086	P3	c.1452delins GCA	p.Asp484Glu fs* 17	Neck	Truncation in the middle of the central domain is predicted to abolish chaperone activity, inhibit protein expression, and render the protein unstable (35575086).	PMID 35575086:<br>- No protein expression when expressed in cultured cells
35575086	P2	c.2182G > A	p.Glu728Lys	UCS	In highly conserved motifs of 2 different helices (13H2 and 15H3) within the UCS domain. This surface-residue mutation in the UCS domain is compatible with the overall protein structure. Possibly induces electrostatic repulsion and	PMID 35575086:<br>- No effect on protein expression when expressed in cells

					surface charge alterations that may affect interactions between the 2 UCS helices and induce a local structural rearrangement.	
39403551	P1	c.2225 T>C	p.Leu742Pro[2] p.Leu757Pro	UCS	The rigid pyrrolidine ring of proline may cause substantial changes in peptide chain backbone angles destabilize helices. This residue is next to T758 and N759 which are directly involved in myosin motor domain interaction.	ND
39403551	P1	c.2297 A>T	p.Glu766Val[2] p.Glu781Val	UCS	Because Glu is often found on the exterior of proteins interacting with water, and Val is usually hidden in the interior, this substitution can lead to severe protein instability or improper conformation.	ND
39403551	P1	c.2348 G>A	p.Cys783Tyr[2] p.Cys798Tyr	UCS	Because Tyr cannot form disulfide bonds, replacing a critical Cys residue often destroys existing disulfide bridges, and may cause disruptions in the protein's native tertiary structure.	ND
41081434/ 36587802	P1/ P2	c.2455C > T	p.Arg819*	UCS	The truncation results in an incomplete UCS domain, generating an unstable protein and inhibiting protein expression.	PMID 36587802:<br>- No protein expression when expressed in HEK cells
35575086	P3	c.2512G > C	p.Ala838Pro	UCS	In highly conserved motifs of 2 different helices (13H2 and 15H3) within the UCS domain. The proline residue may introduce a kink in the UCS domain helix, thereby destabilizing it.	PMID 35575086:<br>- No effect on protein expression when expressed in cells

36699472	P3	c.2534-2545del	p.Leu845-Met848del	UCS	The deletion of four residues shortens the UCS 15H-helix and also truncates the linker to the following helix, which may compromise the domain's structural integrity and alter the conformation of the myosin-binding groove.	ND
29429573/ 29429573	P5/ P6	c.2581C > T	p.Gln861*[2] p.Gln876*	UCS	Truncation at position 876 results in a short, likely unstable UCS domain, which would severely destabilize the myosin-binding groove.	- No protein expression (in patient cells carrying in addition p.Ser893Leu and p.Cys927Gly)
29429573/ 29429573	P5/ P6	c.2633C > T	p.Ser878Leu[2] p.Ser893Leu	UCS	The structural consequences are mild; mutation of the polar surface serine to the hydrophobic leucine eliminates some stabilizing interactions and alters the surface properties, rendering it less polar.	- No protein expression (in patient cells carrying in addition p.Gln876* and p.Cys927Gly)
29429573/ 29429573	P5/ P6	c.2734 T > G	p.Cys912Gly[2] p.Cys927Gly	UCS	The substitution of cysteine by glycine introduces increased main-chain flexibility in the helix but remains compatible with the overall protein structure.	- No protein expression (in patient cells carrying in addition p.Gln876* and p.Ser893Leu)

ND: not determined

[#] The predicted consequences on protein structure/function were inferred from structural analysis of the protein residues localization and involvement in intramolecular interactions or in interactions with partner proteins, and have been reported before by: Sun M, Pylypenko O, Zhou Z, Xu M, Li Q, Houdusse A, van IJzendoorn SCD. Uncovering the Relationship Between Genes and Phenotypes Beyond the Gut in Microvillus Inclusion Disease. Cell Mol Gastroenterol Hepatol. 2024;17(6):983-1005. doi: 10.1016/j.jcmgh.2024.01.015, and by: Duclaux-Loras R, Lebreton C, Berthelet J, Charbit-Henrion F, Nicolle O, Revenu des Courtils C, Waich S, Valovka T, Khiat A, Rabant M, Racine C, Guerrera IC, Baptista J, Mahe MM, Hess MW, Durel B, Lefort N, Banal C, Parisot M, Talbotec C, Lacaille F, Ecochard-Dugelay E, Demir AM, Vogel GF, Faivre L, Rodrigues A, Fowler D, Janecke AR, Müller T, Huber LA, Rodrigues-Lima F, Ruemmele FM, Uhlig

HH, Del Bene F, Michaux G, Cerf-Bensussan N, Parlato M. UNC45A deficiency causes microvillus inclusion disease-like phenotype by impairing myosin VB-dependent apical trafficking. J Clin Invest. 2022 May 16;132(10):e154997. doi: 10.1172/JCI154997.

[1]. This variant was originally reported using the amino acid numbering of UNC45A isoform 2 (Q9H3U1-2; NP_001310548.1), which lacks a 15-amino acid segment at the N-terminus compared to the canonical sequence. To ensure consistency with the canonical reference (Q9H3U1-1; NP_061141.2) and the authors' cited reference transcript ENST00000394275.6 (944 aa), we have remapped the position to c.292C>T (p.Arg98Trp). All subsequent mentions of this variant follow the canonical numbering.

[2]. Variants marked with an uppercase [2] and displayed in grey were originally reported in PMID references 29429573 and 39403551 using the amino acid numbering of the shorter isoform Q9H3U1-2 (929 aa). For consistency within this table, positions have been corrected to match the authors' cited reference transcript ENST00000394275.6 (944 aa), resulting in a +15 amino acid offset from the original report.

Zhe et al. Figure 2 A

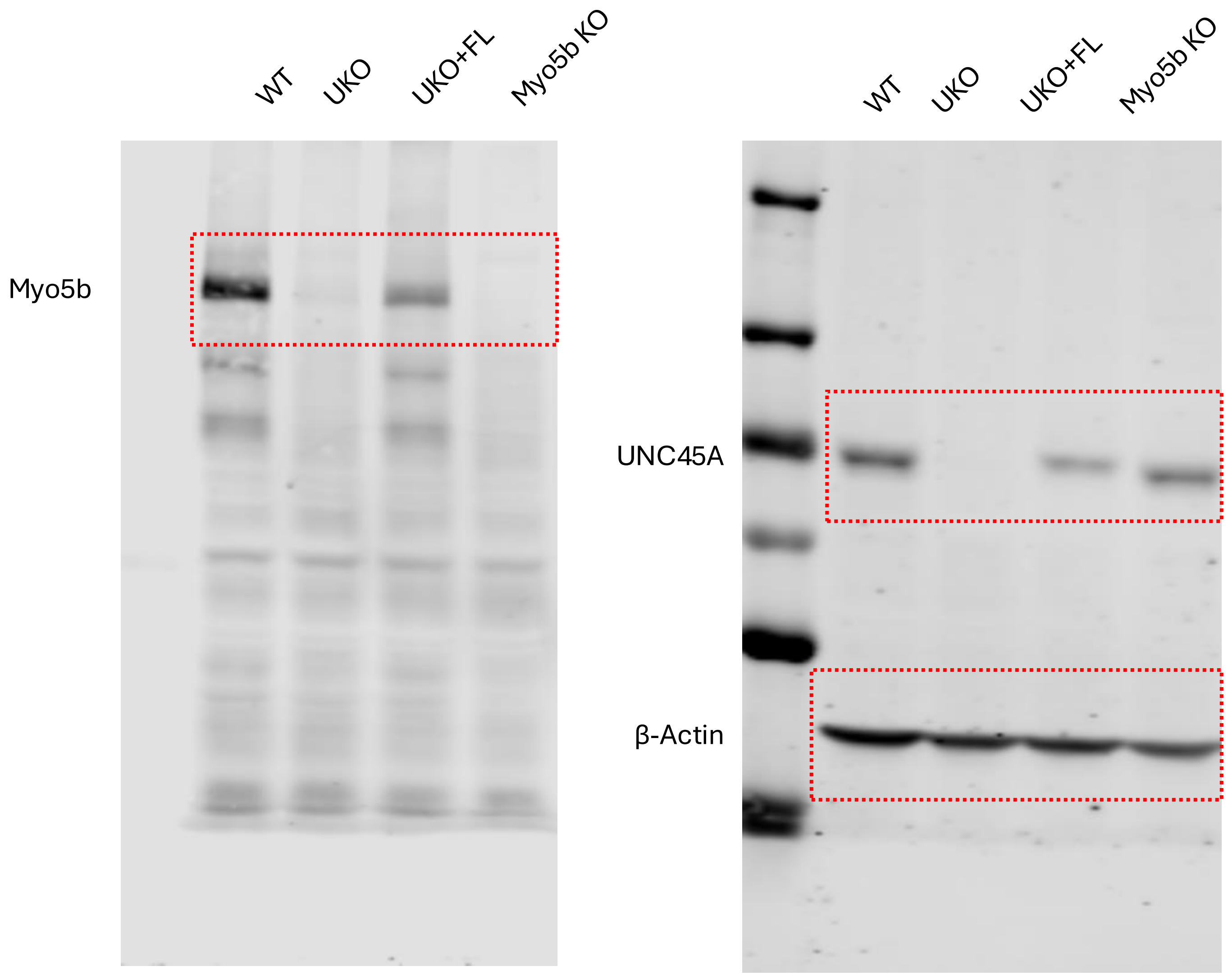

Zhe et al. Figure 4 B

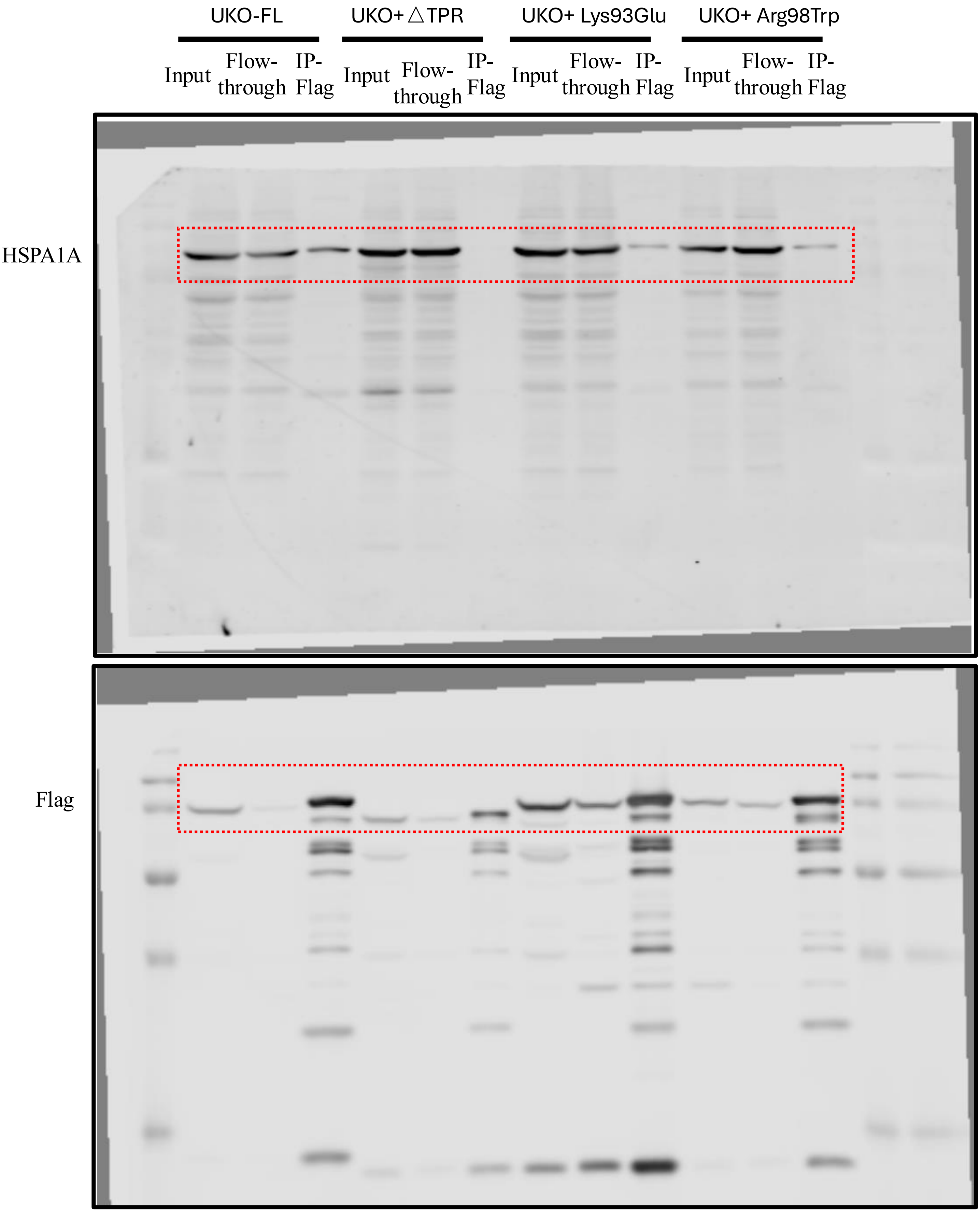

Zhe et al. Figure 4 B

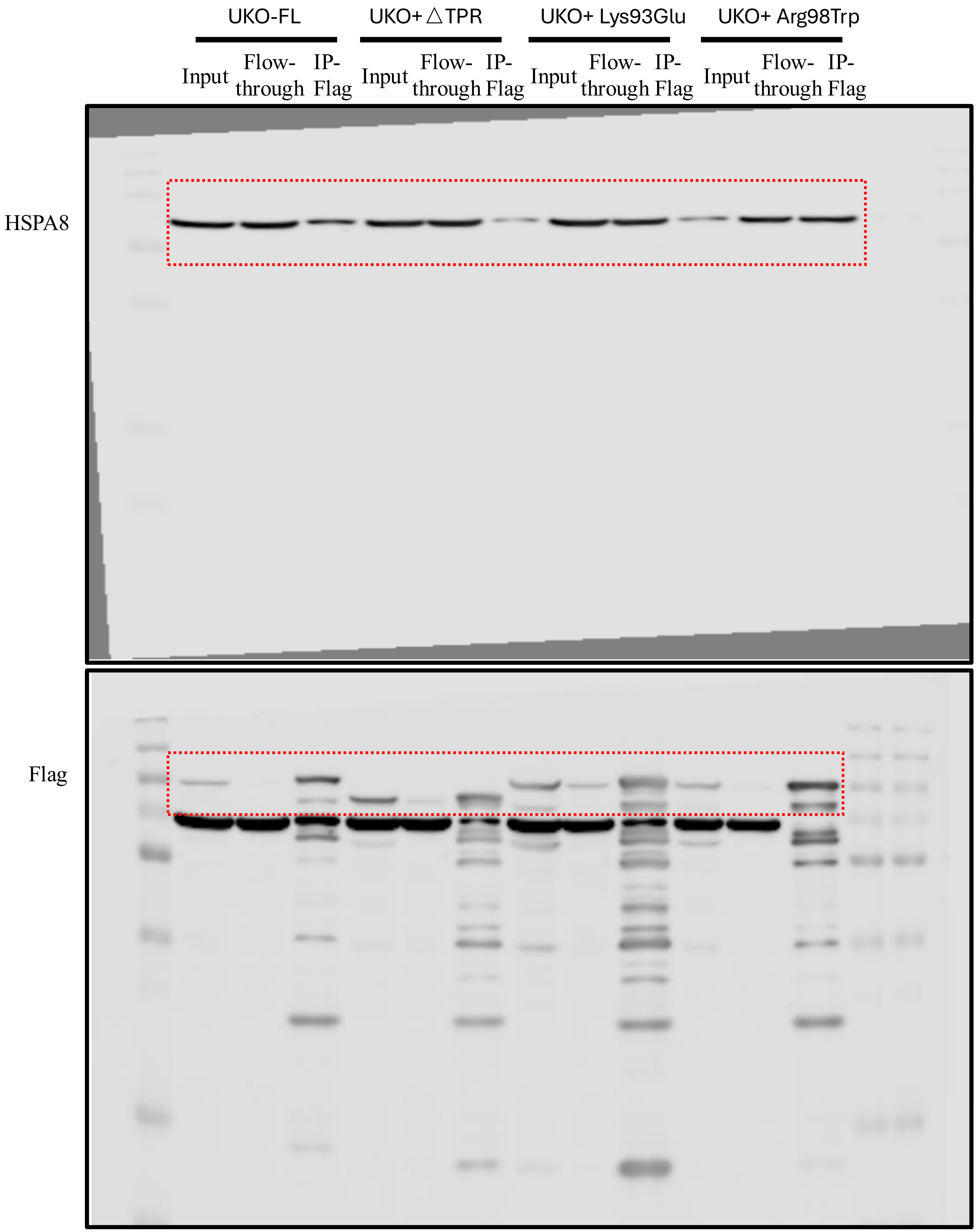

Zhe et al. Figure 4 B

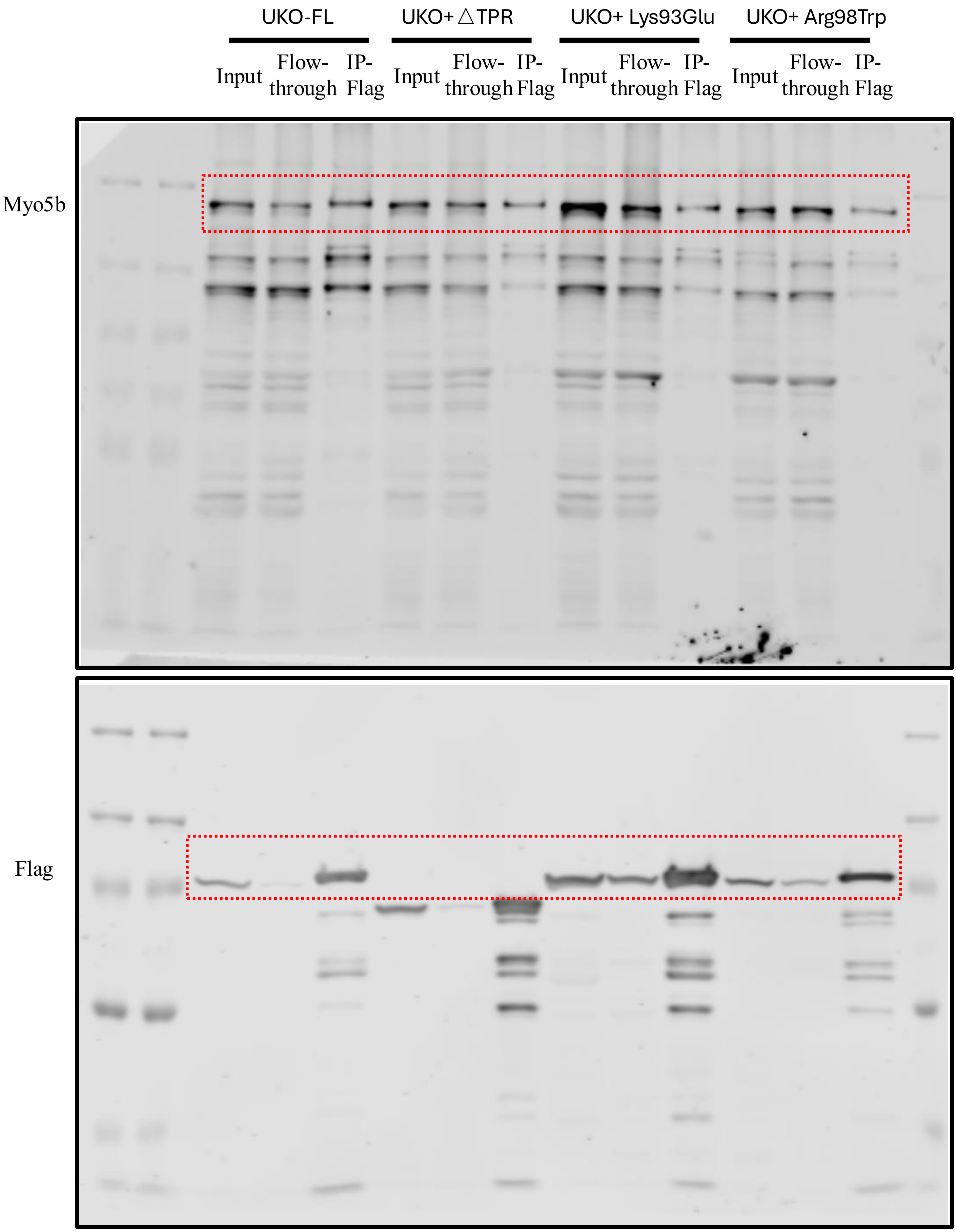

Zhe et al. Figure 6 A

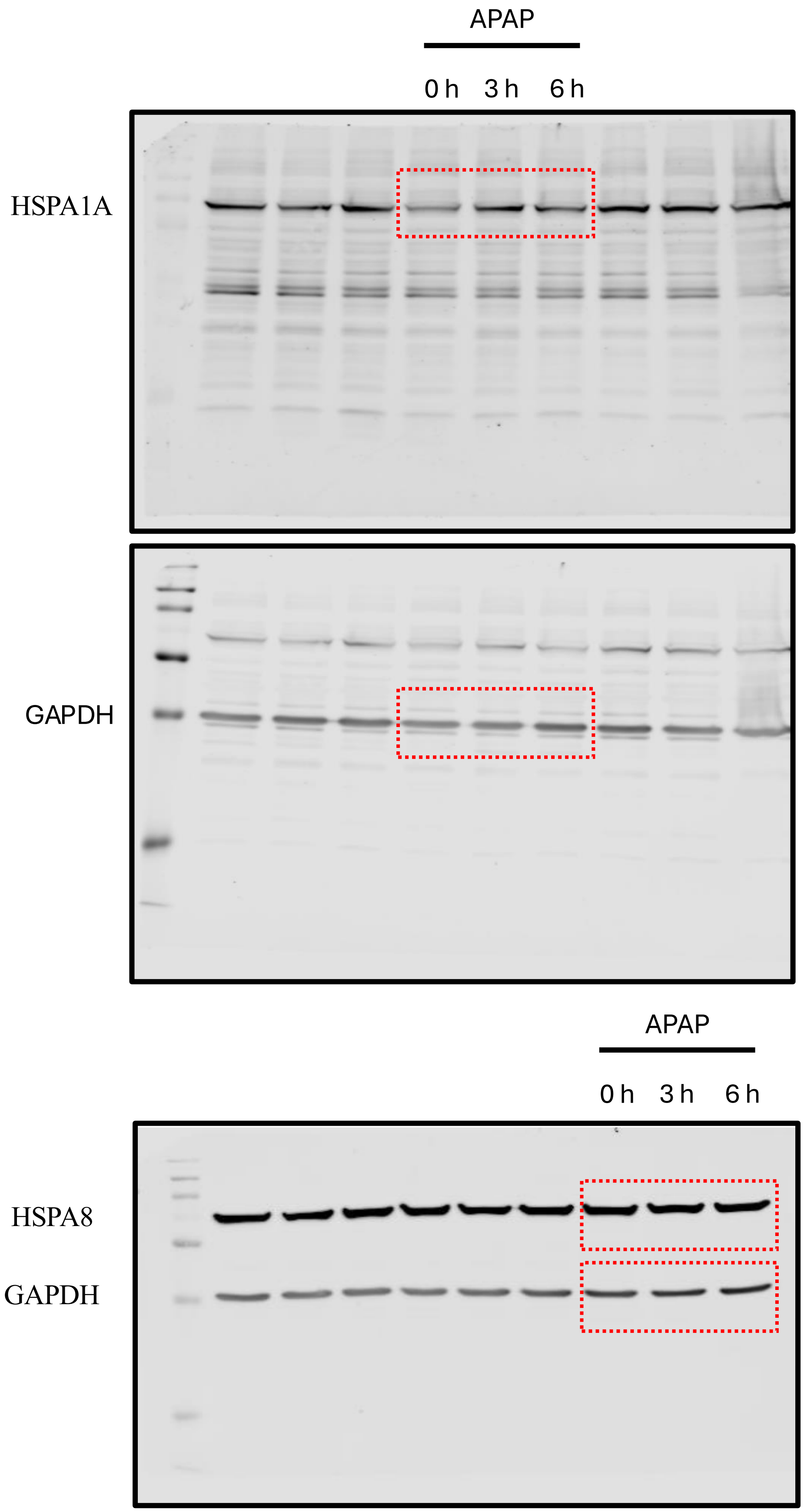

Zhe et al. Figure 6 B

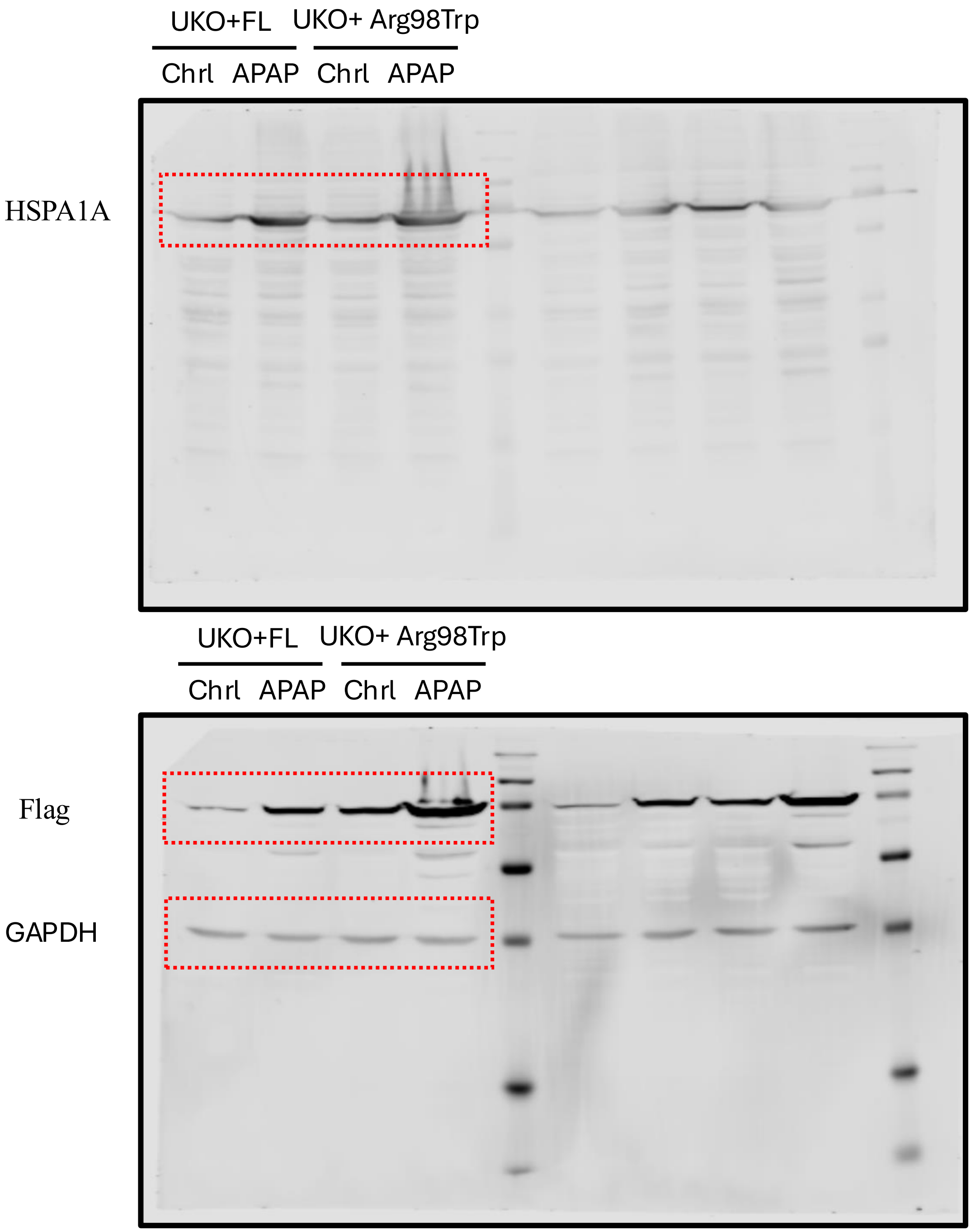

Zhe et al. Supplementary Figure S1 A

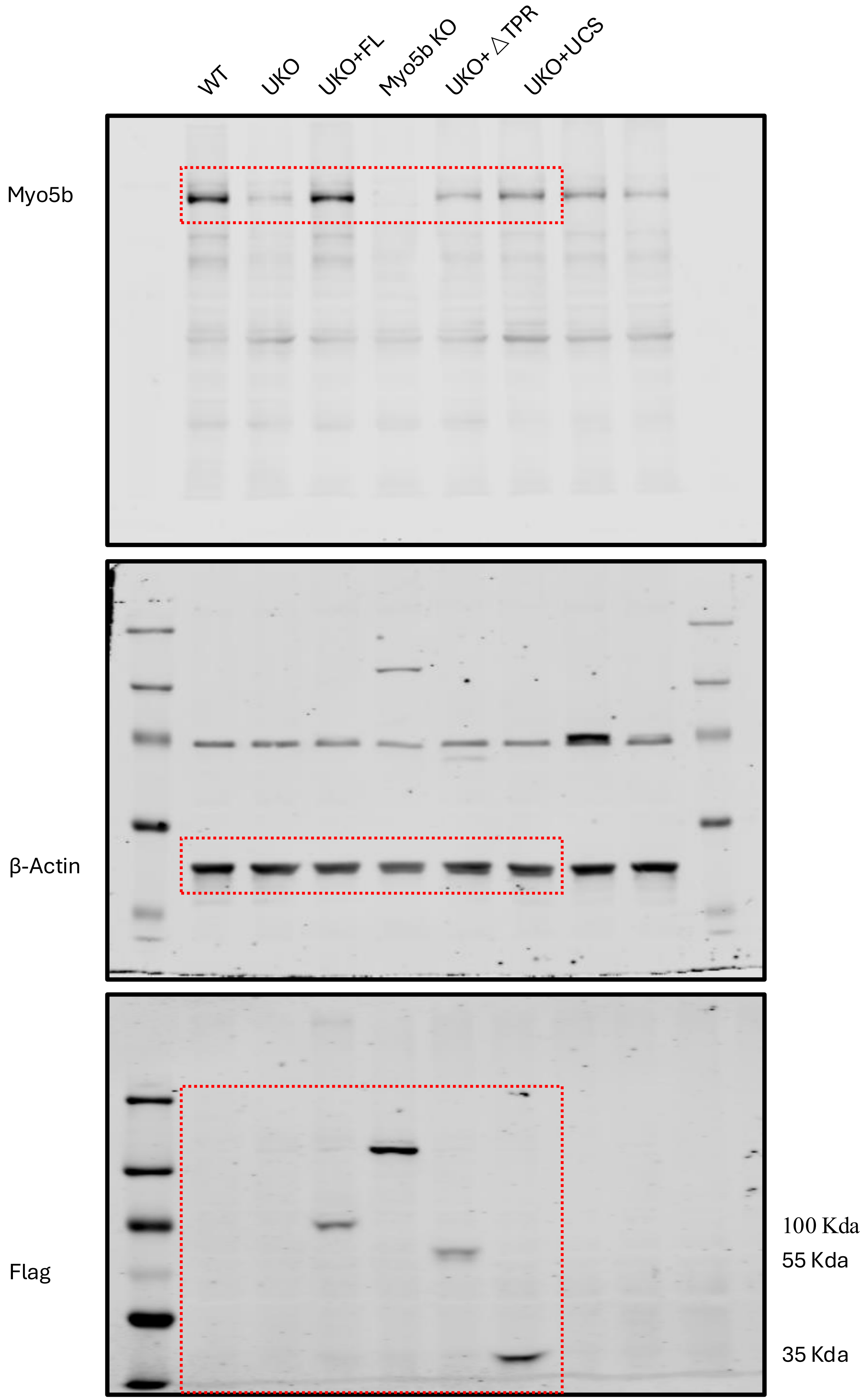

Zhe et al. Supplementary Figure S3 A

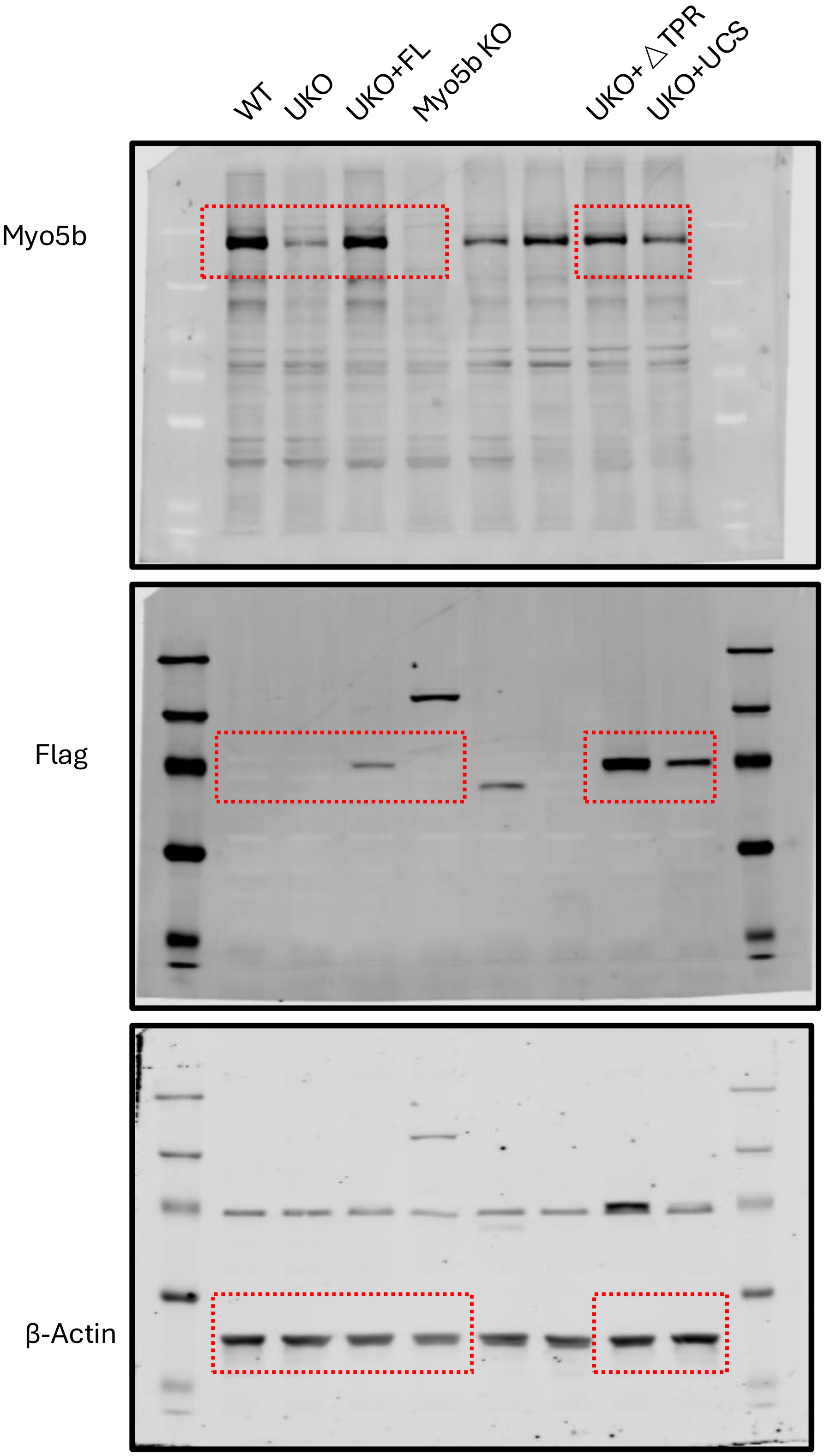

Zhe et al. Supplementary Figure S5 A

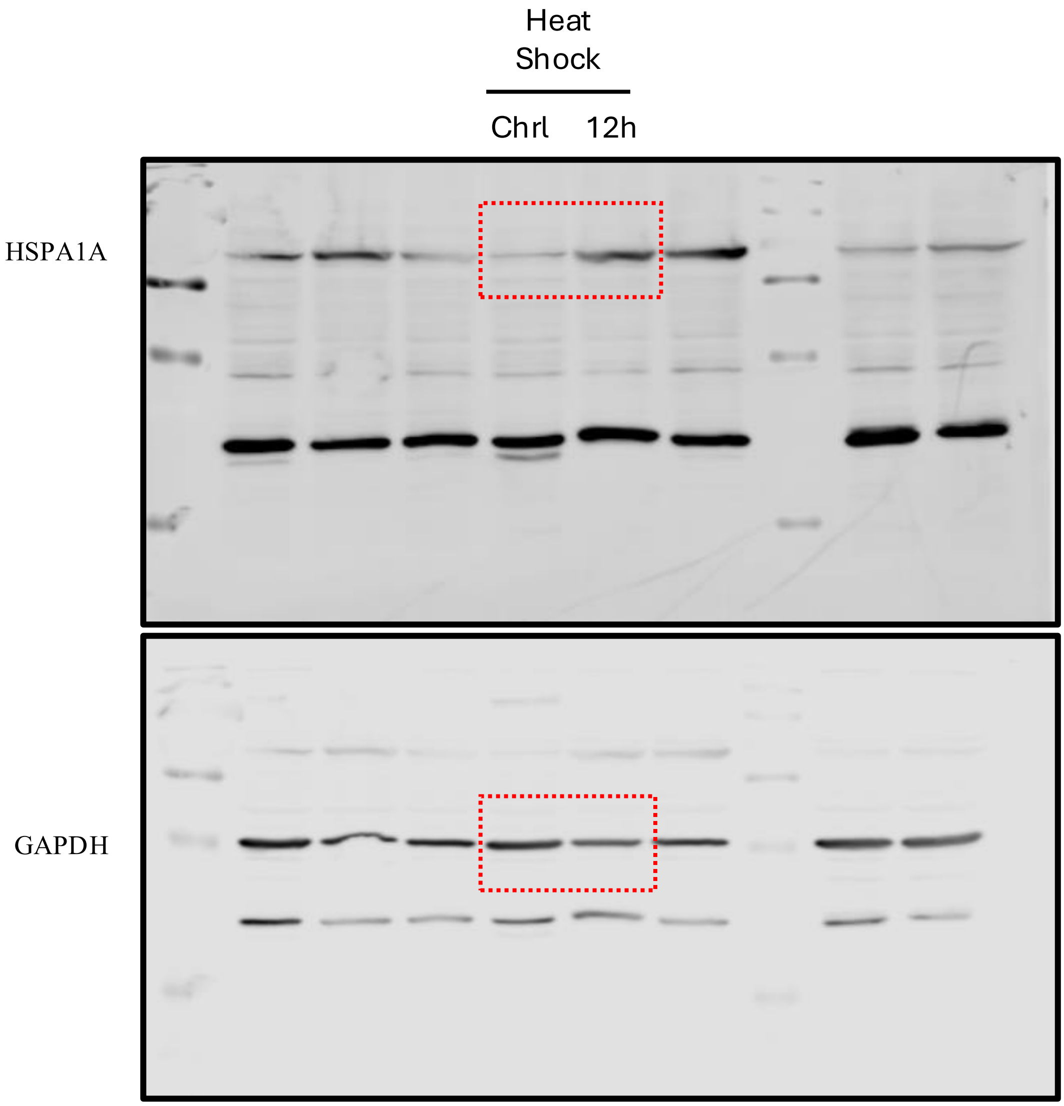

Zhe et al. Supplementary Figure S5 A

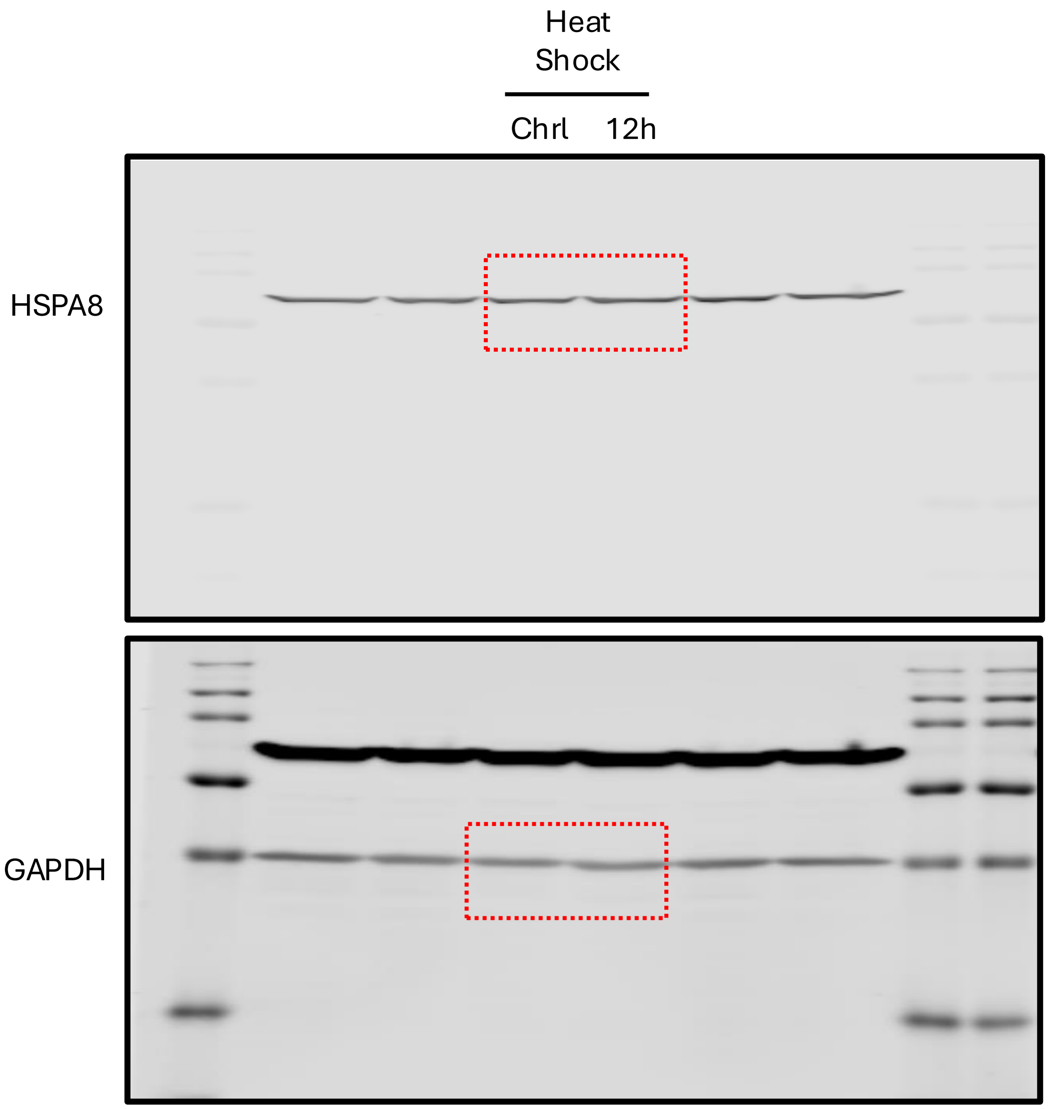

Zhe et al. Supplementary Figure S5 B

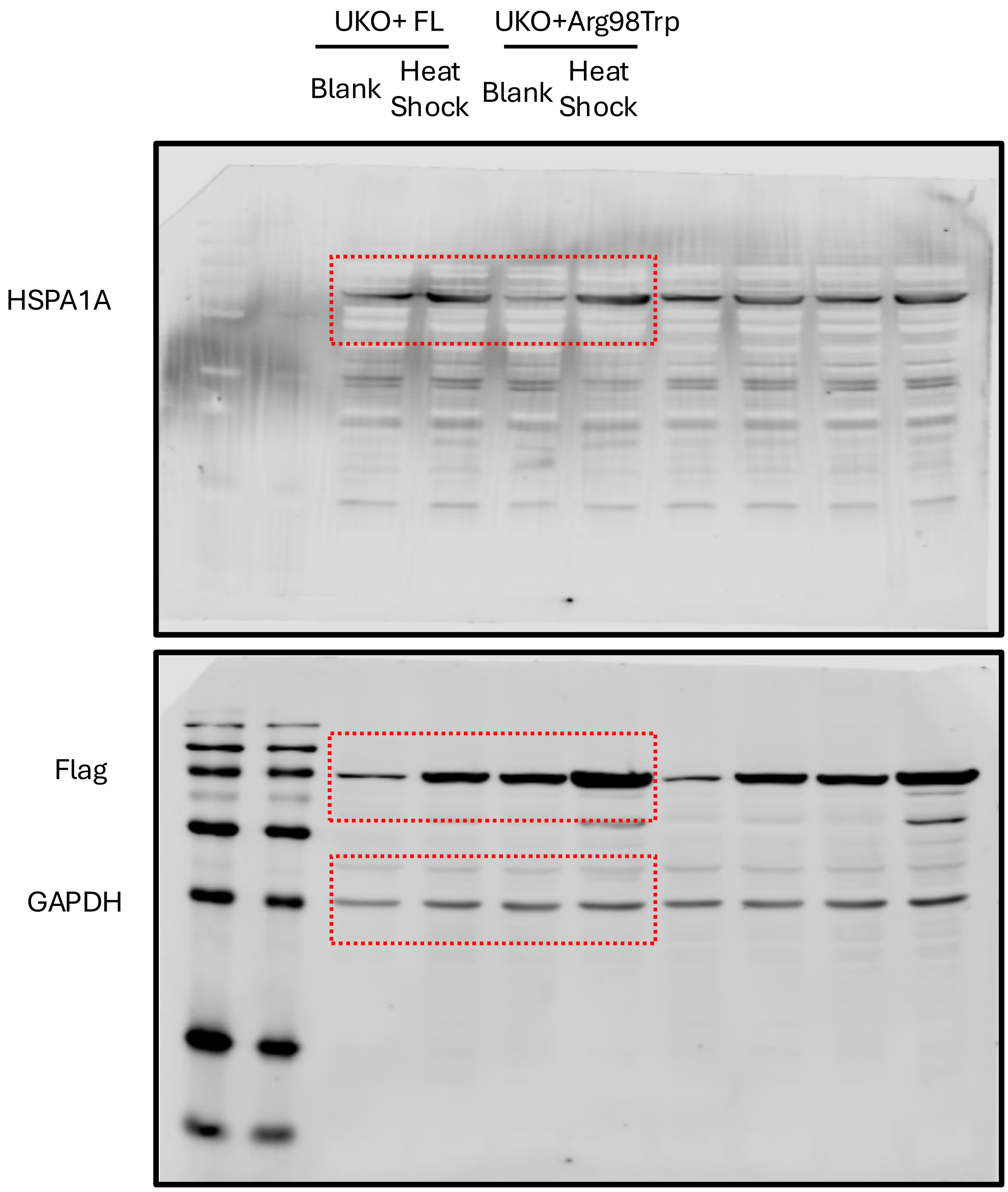

Zhe et al. Supplementary Figure S7 A

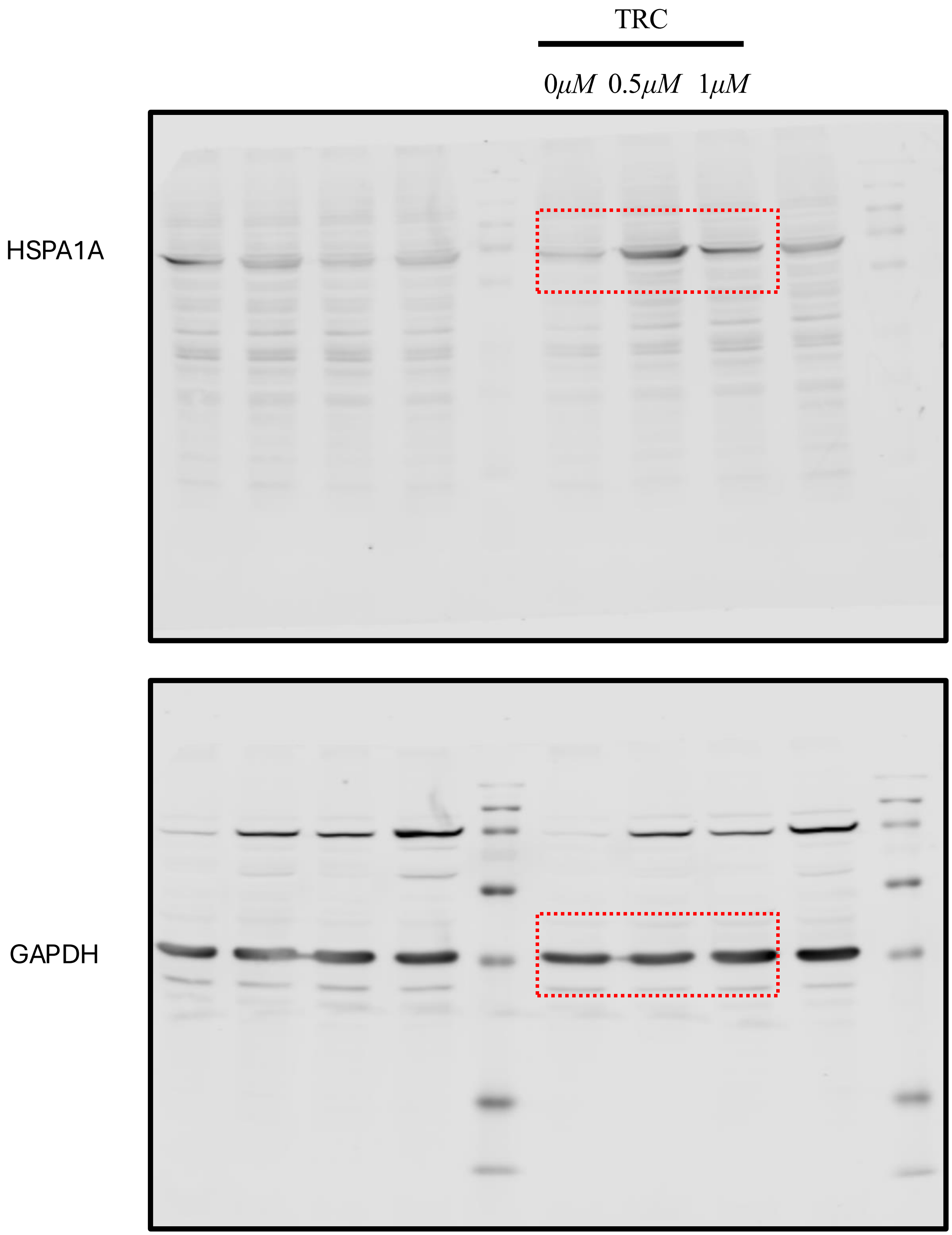

Zhe et al. Supplementary Figure S7 A

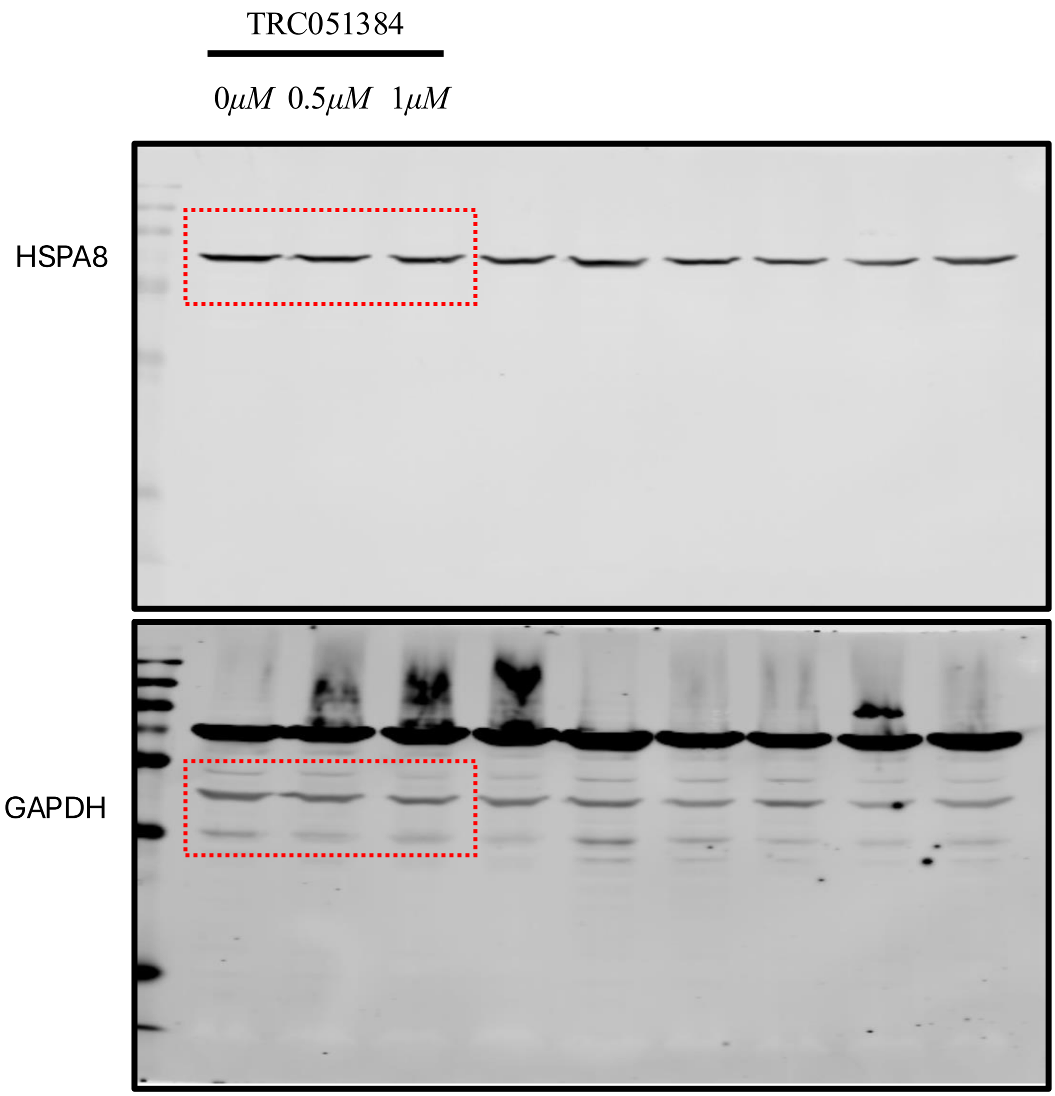

Zhe et al. Supplementary Figure S7 B

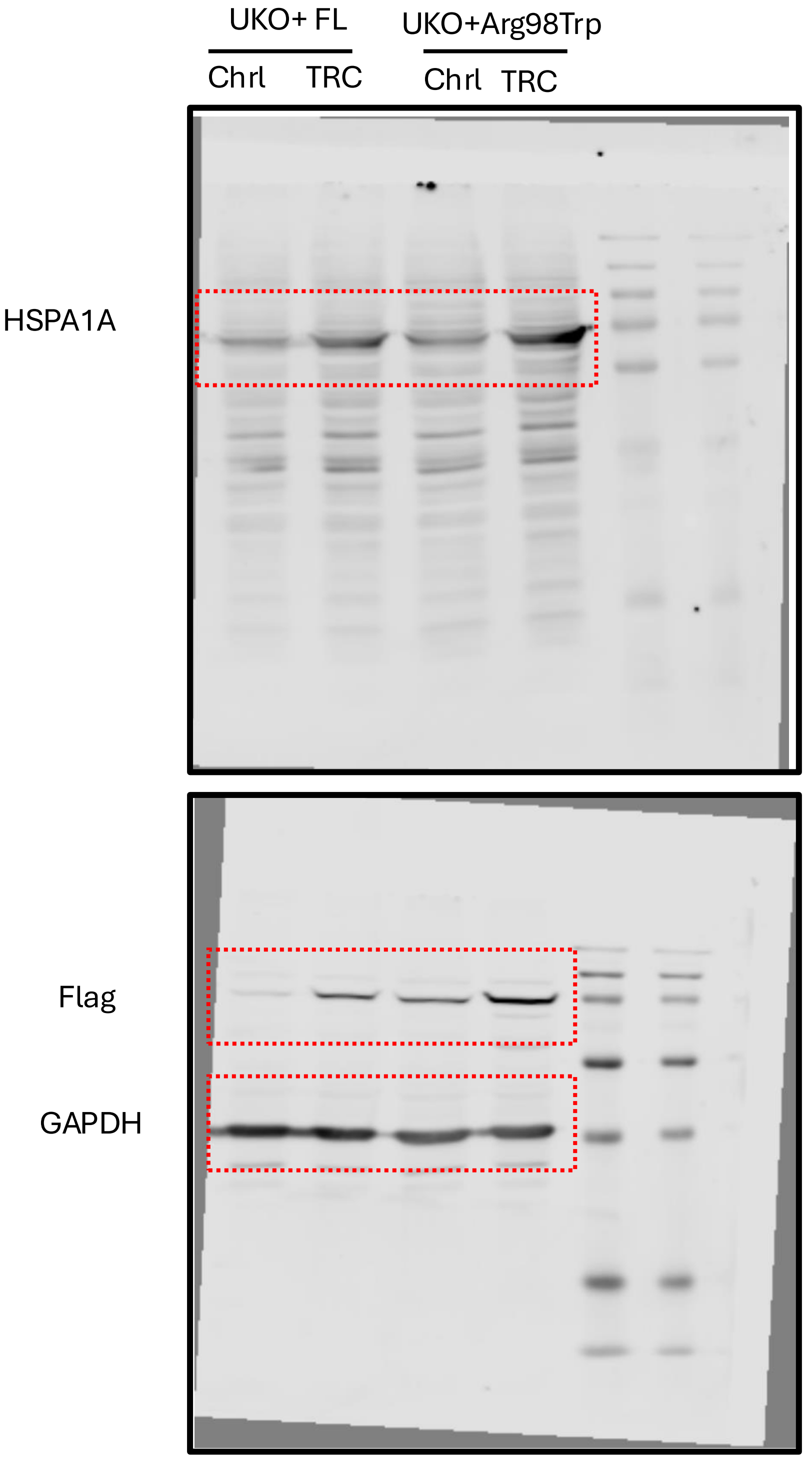

Zhe et al. Supplementary Figure S9 B

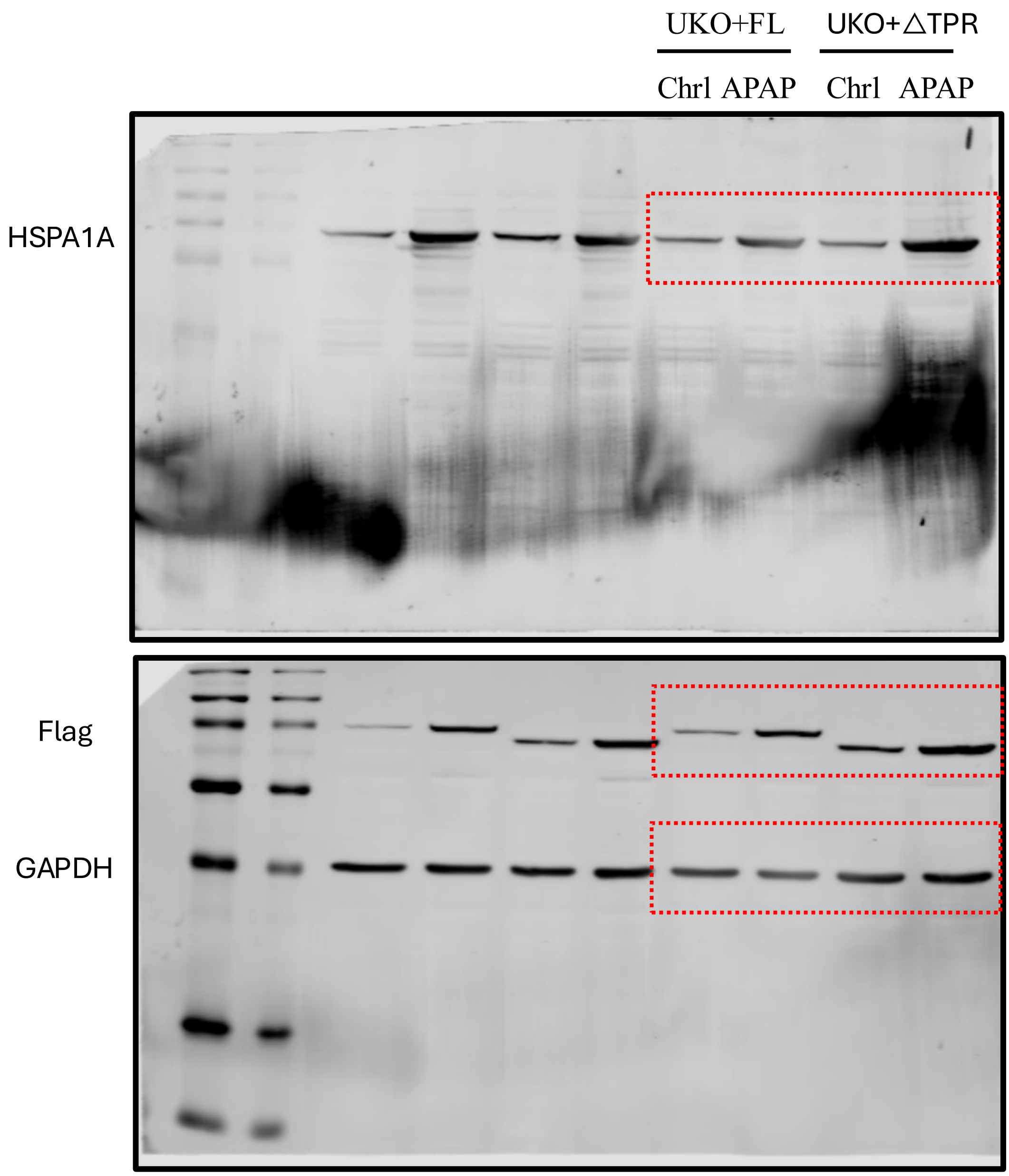

Supplement: Extended PDF [file mmc3.pdf]
